# Venom proteomics and antivenom neutralization for the Chinese eastern Russell’s viper, *Daboia siamensis* from Guangxi and Taiwan

**DOI:** 10.1038/s41598-018-25955-y

**Published:** 2018-06-04

**Authors:** Kae Yi Tan, Nget Hong Tan, Choo Hock Tan

**Affiliations:** 1 Department of Molecular Medicine, Faculty of Medicine, University of Malaya, Kuala Lumpur, Malaysia; 2 Department of Pharmacology, Faculty of Medicine, University of Malaya, Kuala Lumpur, Malaysia

## Abstract

The eastern Russell’s viper (*Daboia siamensis*) causes primarily hemotoxic envenomation. Applying shotgun proteomic approach, the present study unveiled the protein complexity and geographical variation of eastern *D*. *siamensis* venoms originated from Guangxi and Taiwan. The snake venoms from the two geographical locales shared comparable expression of major proteins notwithstanding variability in their toxin proteoforms. More than 90% of total venom proteins belong to the toxin families of Kunitz-type serine protease inhibitor, phospholipase A_2_, C-type lectin/lectin-like protein, serine protease and metalloproteinase. *Daboia* *siamensis* Monovalent Antivenom produced in Taiwan (DsMAV-Taiwan) was immunoreactive toward the Guangxi *D*. *siamensis* venom, and effectively neutralized the venom lethality at a potency of 1.41 mg venom per ml antivenom. This was corroborated by the antivenom effective neutralization against the venom procoagulant (ED = 0.044 ± 0.002 µl, 2.03 ± 0.12 mg/ml) and hemorrhagic (ED_50_ = 0.871 ± 0.159 µl, 7.85 ± 3.70 mg/ml) effects. The hetero-specific Chinese pit viper antivenoms i.e. *Deinagkistrodon acutus* Monovalent Antivenom and *Gloydius brevicaudus* Monovalent Antivenom showed negligible immunoreactivity and poor neutralization against the Guangxi *D*. *siamensis* venom. The findings suggest the need for improving treatment of *D*. *siamensis* envenomation in the region through the production and the use of appropriate antivenom.

## Introduction

*Daboia* is a genus of the Viperinae subfamily (family: Viperidae), comprising a group of vipers commonly known as Russell’s viper native to the Old World^1^. The Russell’s viper was previously recognised as monotypic *Daboia russelii* or *Vipera russelii* with at least seven subspecies following an extremely disjunct distribution over a large area of Asian countries, from Pakistan, India, Sri Lanka, Myanmar, Thailand, Cambodia, Java and islands of Lesser Sunda in Indonesia, to South China (Guangdong and Guangxi) and the insular Taiwan. Based on mitochondrial DNA and multivariate morphological analyses, Thorpe *et al*.^2^ suggested that the Russell’s viper complex diverged approximately 7–11 mybp (million years before present) into the eastern and the western clades, separated by a narrow range of mountains in northwest Burma to the north of the Bay of Bengal. This led to the revision of the entire Russell’s viper complex systematics, sinking several subspecies into synonyms that followed biogeographical distribution while elevating *Daboia russelii russelii* and *Daboia russelii siamensis* to their respective full species status. Currently, *Daboia russelii* represents the Western Russell’s viper that is indigenous to South Asia, while the Eastern Russell’s viper (*Daboia siamensis*) distributes in Southeast and East Asia, comprising the former subspecies *limitis*, *sublimitis* and *formosensis*^2–4^.

The differences between the two species of Russell’s vipers are, in fact, not limited to their morphology and molecular phylogenies. Differences in the envenoming effects of the Russell’s viper have been reported, attributable to the plasticity of snake venom as an adaptive polygenic trait of venomous snakes^5,6^. The observed variations of the envenomation, however, did not conform to the phylogenetics and systematics^7–9^. This implies that within each *Daboia* species, venom variation is common and the investigation of the venom composition should be directed toward detailed venom characterization based on the distinctive species and the geographical locale from where the venom originates. Indeed, the pathogenesis of snakebite envenomation correlates with venom composition, and it is well established that even within the same species of *D*. *russelii*, the venom composition can vary across different locales^10–13^. With the recent advent of proteomic technologies, the compositions of *D*. *russelii* venoms of different regions in South Asia (Pakistan, western India, southern India and Sri Lanka) have been unravelled to great details, improving our understanding of the clinicopathological correlation and effectiveness of antivenom treatment^12–16^. For instance, the Sri Lankan *D*. *russelii* venom contain substantial neurotoxic phospholipases A_2_ that correlated with the neurotoxic activity of the venom in animal experiment and clinical envenomation^8,15^. In contrast, the proteomic characterization of *D*. *siamensis* venom received less attention although envenoming by this species remains prevalent in many parts of the world including the southern mainland of China^17–20^, insular Taiwan^21^, Indonesia^22^, Thailand^23,24^ and Myanmar^25–28^. Several toxins had been isolated previously from *D*. *siamensis* venom, including Kunitz-type serine protease inhibitors^29,30^, phospholipases A_2_^31^, snaclecs^32^, snake venom serine proteases^33^ and snake venom metalloproteinases^34–36^. The venom proteome of the Myanmese *D*. *siamensis* has also been reported^37^; however, the knowledge on the quantitative details and geographical variability of *D*. *siamensis* venom proteins from different locales remain unclear. In particular, the venom proteomes of *D*. *siamensis* of the far eastern lineage, namely those from the mainland of China and insular Taiwan may be geographically varied. The knowledge is much needed for comparative study of Russell’s viper venoms to better understand the clinicopathological correlation of envenomation and the efficacy of antivenom treatment.

*D*. *siamensis* envenomation can cause painful local effect with systemic bleeding disorders, typically manifested as venom-induced consumptive coagulopathy^38^ which may be accompanied with complications such as hypopituitarism and renal failure^25,39–41^. In particular, acute or chronic hypopituitarism is associated more commonly with clinical cases from Myanmar^39,42^, although this effect has also been noted recently in a few cases from Sri Lanka (*D*. *russelii* envenomation)^9,26^. The envenomation by *D*. *siamensis*, however, unlike envenoming by the Sri Lankan *D*. *russelii*, rarely produces neuromuscular paralysis in the envenomed patients^21^. *D*. *siamensis* is locally known as “round-spot viper” () in the China mainland and “chain snake/viper” () in Taiwan Island. The incidence of snakebite and antivenom treatment of *D*. *siamensis* envenomation, however, differ across the Strait. In general, *D*. *siamensis* envenomation affects the agricultural populations and people engaging in field activities; nonetheless, in areas where venomous snakes are bred or sought for local delicacy and health supplement, the snake farmers, traders and handlers including cooks also bear the risk of envenomation. Literature on Chinese *D*. *siamensis* envenomation is, however, scarce and less accessible as most clinical reports were lodged in the Chinese depository^17–20^. Where antivenom treatment is concerned in the two geographical areas, the specific antivenom indicated for *D*. *siamensis* envenoming, herewith known as *D*. *siamensis* Monovalent Antivenom (DsMAV-Taiwan) is only available in Taiwan, despite the fact that *D*. *siamensis* is also distributed across the southern part of the mainland of China. The unavailability of *D*. *siamensis* antivenom led to the non-specific use of hetero-specific “viperid” Chinese antivenoms i.e. the *Gloydius brevicaudus* (short-tailed Chinese mamushi) Monovalent Antivenom (GbMAV) and *Deinagkistrodon acutus* (sharp-nosed pit viper) Monovalent Antivenom (DaMAV), either singly or combined to treat *D*. *siamensis* envenoming clinically. Failure of treatment including death outcome has been reported anecdotally following the administration of these inappropriate antivenoms.

Worldwide, there are at least two major antivenom manufacturers that produce specific antivenom against the eastern Russell’s viper: (1) In Taiwan, the Centers for Disease Control (CDC) produces the Taiwanese *D*. *siamensis* Monovalent Antivenom (DsMAV-Taiwan); (2) In Thailand, the Queen Saovabha Memorial Institute (QSMI) produces the Thai *D*. *siamensis* Monovalent Antivenom (DsMAV-Thai) and Hemato Polyvalent Antivenom (a polyvalent antivenom raised against three Viperidae snakes of Thai origin). This study aimed to investigate and compare the venom proteomes of *D*. *siamensis* from Guangxi and Taiwan in correlation with the toxicity of the venoms. The immunoreactivity of different antivenoms and neutralization of the venoms were also investigated.

## Result

### SDS-PAGE and proteomes of *Daboia siamensis* venoms

**Figure 1:**
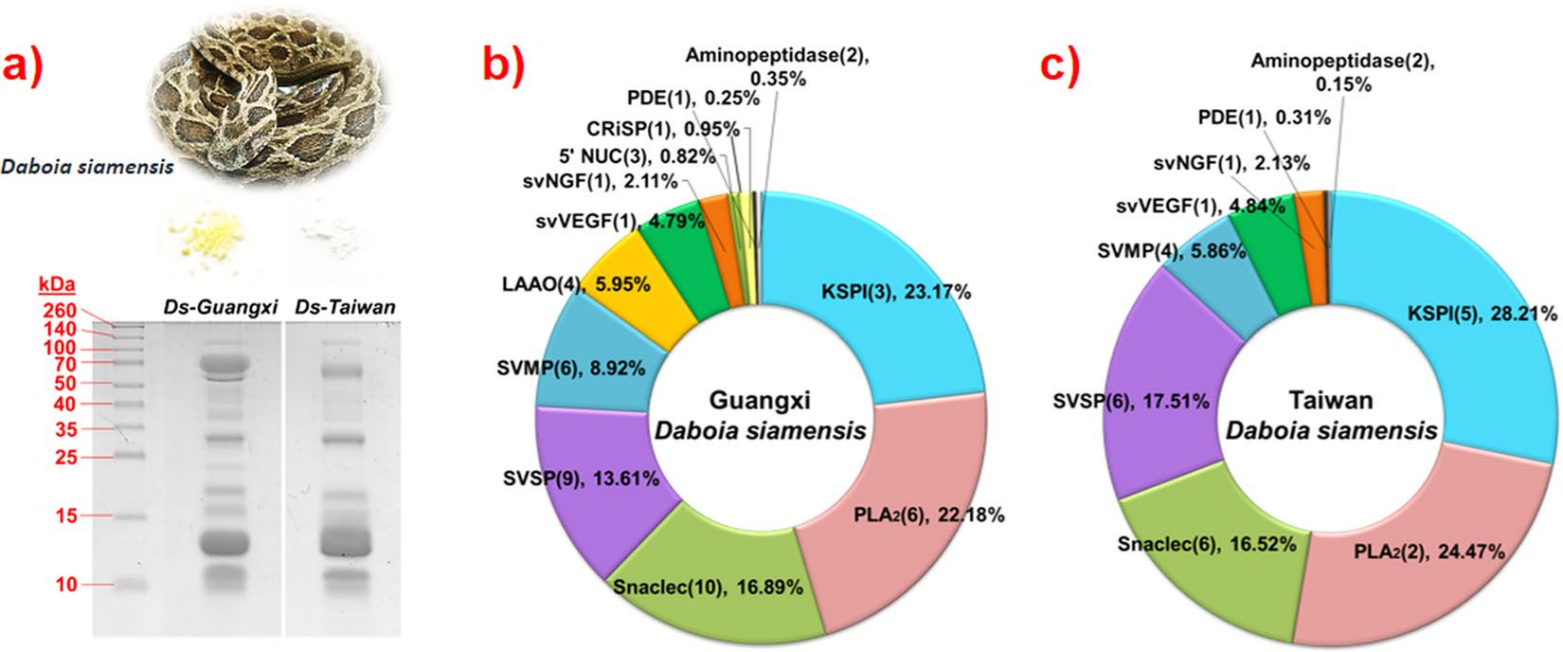
SDS-PAGE and proteomes of *Daboia siamensis* venoms. (**a**) *D. siamensis* and lyophilized venoms (top) and protein separation of Ds-Guangxi and Ds-Taiwan venoms on 15% SDS-PAGE under reducing conditions (bottom). (**b**) Venom proteome of Ds-Guangxi. (**c**) Venom proteome of Ds-Taiwan. The number of proteoforms of each protein family is in parentheses. Abbreviations: Ds-Guangxi: *D*. *siamensis* of Guangxi (mainland); Ds-Taiwan, *D. siamensis* of Taiwan (island); KSPI, Kunitz-type serine protease inhibitor; PLA_2_, phospholipase A_2_; Snaclec, snake venom C-type lectin/lectin-like protein; SVSP, snake venom serine protease; SVMP, snake venom metalloproteinase; LAAO, L-amino acid oxidase; svVEGF, snake venom vascular endothelial growth factor; svNGF, snake venom nerve growth factor; 5′NUC, 5′-nucleotidase; CRiSP, cysteine-rich secretory protein; PDE, phosphodiesterase. Note: SDS-PAGE of Ds-Guangxi and Ds-Taiwan venoms were cropped from the same gel for display purpose. The full-length gel is provided in the Supplementary Information File S1.

The venoms of *D. siamensis* from Guangxi (Ds-Guangxi) and Taiwan (Ds-Taiwan) were separated by 15% SDS-PAGE under reducing conditions. Electrophoretic bands corresponding to proteins with molecular weights ranging from below 10 to 140 kDa were observed as shown in Fig. 1(a). The proteins in both the venoms shared a similar pattern of band distribution, while differences in the gel band density were noted in the proteins of 13–15 kDa (more intense in Ds-Taiwan) and 70–140 kDa (more intense in Ds-Guangxi). Nano-ESI-LCMS/MS analyses revealed that there were a total of 47 proteins constituting 12 protein families in the Ds-Guangxi venom and 28 proteins constituting 9 protein families in the Ds-Taiwan venom (Table 1). The majority of venom protein families were shared between Ds-Guangxi and Ds-Taiwan venoms whereas L-amino acid oxidase (LAAO), 5′-nucleotidase (5′NUC) and cysteine-rich secretory protein (CRiSP) were only detected in the Ds-Guangxi venom.

**Table 1:** Proteomes of *Daboia siamensis* venoms from Guangxi and Taiwan profiled using nano-ESI-LCMS/MS.

Protein Name	Database Accession	Species	*D*. *siamensis* (Guangxi)		*D*. *siamensis* (Taiwan)	
			%	Proteoform	%	Proteoform
**Kunitz-type serine protease inhibitor (KSPI)**			**23**.**17**	**3**	**28**.**21**	**5**
Kunitz-type serine protease inhibitor C1	A8Y7N4	*D*. *siamensis*	—	—	12.18	1
Kunitz-type serine protease inhibitor C4	A8Y7N7	*D*. *siamensis*	—	—	3.51	2
Kunitz-type serine protease inhibitor B4	A8Y7P4	*D*. *siamensis*	6.53	1	3.43	3
Kunitz-type serine protease inhibitor B5	A8Y7P5	*D*. *siamensis*	4.91	2	—	—
Kunitz-type serine protease inhibitor B6	A8Y7P6	*D*. *siamensis*	—	—	1.77	4
Kunitz-type serine protease inhibitor 2	P00990	*D*. *siamensis*	11.72	3	7.31	5
**Phospholipase A**_**2**_ **(PLA**_**2**_ **)**			**22**.**18**	**6**	**24**.**47**	**2**
Acidic phospholipase A_2_ RV-7	P31100	*D*. *siamensis*	—	—	10.87	1
Basic phospholipase A_2_ RV-4	Q02471	*D*. *siamensis*	—	—	13.61	2
Acidic phospholipase A_2_ daboiatoxin A chain	Q7T2R1	*D*. *siamensis*	5.30	1	—	—
Acidic phospholipase A_2_ daboiatoxin B chain	Q7T3T5	*D*. *siamensis*	4.29	2	—	—
Basic phospholipase A_2_ DsM-b1	A8CG82	*D*. *siamensis*	2.38	3	—	—
Acidic phospholipase A_2_ DsM-a2	A8CG78	*D*. *siamensis*	4.21	4	—	—
Basic phospholipase A_2_ Drk-b1	A8CG89	*D*. *russelii*	5.05	5	—	—
phospholipase A_2_-I	Q7ZZQ1	*D*. *siamensis*	0.96	6	—	—
**Snake venom C-type lectin/lectin-like protein (snaclec)**			**16**.**89**	**10**	**16**.**52**	**6**
Snaclec dabocetin subunit alpha	Q38L02	*D*. *siamensis*	1.35	1	—	—
Snaclec A12	B4XSY7	*M*. *lebetina*	0.64	2	—	—
C-type lectin A12	Unigene30367_DrSL*	*D*. *russelii*	0.28	3	—	—
Snaclec 7	Q4PRC6	*D*. *siamensis*	2.93	4	—	—
Snaclec 5	Q4PRC8	*D*. *siamensis*	1.02	5	—	—
Snaclec 4	Q4PRC9	*D*. *siamensis*	2.01	6	1.43	1
Snaclec 3	Q4PRD0	*D*. *siamensis*	1.48	7	1.03	2
P31 alpha subunit	K9JBU9	*D*. *siamensis*	0.48	8	—	—
P68 alpha subunit	K9JBV0	*D*. *siamensis*	5.86	9	5.95	3
Snaclec coagulation factor X-activating enzyme light chain 1	Q4PRD1	*D*. *siamensis*	—	—	4.77	4
Snaclec coagulation factor X-activating enzyme light chain 2	Q4PRD2	*D*. *siamensis*	0.84	10	2.07	5
Factor X activator light chain 2	K9JDJ1	*D*. *siamensis*	—	—	1.28	6
**Snake venom serine protease (SVSP)**			**13**.**61**	**9**	**17**.**51**	**6**
Alpha-fibrinogenase-like	E5L0E3	*D*. *siamensis*	0.77	1	1.01	1
Beta-fibrinogenase	E0Y419	*M*. *lebetina*	1.57	2	0.74	2
Beta-fibrinogenase-like	E5L0E4	*D*. *siamensis*	2.50	3	—	—
serine beta-fibrinogenase-like protein	CL2958.contig11_DrSL*	*D*. *russelii*	0.71	4	2.38	3
Factor V activator RVV-V gamma	P18965	*D*. *siamensis*	2.79	5	8.11	4
Vipera russelli proteinase RVV-V homolog 2	P86531	*D*. *russelii*	0.59	6	—	—
RVV-V gamma-like protein	CL31.contig2_Nn*	*N*. *naja*	1.14	7	2.19	5
Venom serine proteinase-like protein 2	Q9PT40	*M*. *lebetina*	0.30	8	—	—
Serine protease VLSP-1	CL2958.contig6_DrSL*	*D*. *russelii*	3.25	9	3.07	6
**Snake venom metalloproteinase (SVMP)**			**8**.**92**	**6**	**5**.**86**	**4**
Zinc metalloproteinase-disintegrin-like daborhagin-K	B8K1W0	*D*. *russelii*	4.44	1	—	—
Zinc metalloproteinase-disintegrin-like VLAIP-A	Q4VM08	*M*. *lebetina*	0.36	2	1.34	1
Zinc metalloproteinase-disintegrin VLAIP-A	CL3662.contig2_DrSL*	*D*. *russelii*	1.02	3	1.62	2
Zinc metalloproteinase-disintegrin VLAIP-A	Unigene31385_Nn*	*N*. *naja*	0.83	4	0.44	3
Coagulation factor X-activating enzyme heavy chain	Q7LZ61	*D*. *siamensis*	—	—	2.45	4
factor X activator heavy chain	K9JAW0	*D*. *russelii*	1.03	5	—	—
factor X activator heavy chain	Unigene32626_DrSL*	*D*. *russelii*	1.25	6	—	—
**L-amino acid oxidase (LAAO)**			**5**.**95**	**4**	—	—
L-amino-acid oxidase	G8XQX1	*D*. *russelii*	1.29	1	—	—
L-amino-acid oxidase	P0C2D7	*V*. *berus berus*	1.84	2	—	—
L-amino-acid oxidase	P81382	*C*. *rhodostoma*	0.35	3	—	—
L-amino-acid oxidase	Q4F867	*D*. *siamensis*	2.47	4	—	—
**Snake venom vascular endothelial growth factor (svVEGF)**			**4**.**79**	**1**	**4**.**84**	**1**
Snake venom vascular endothelial growth factor toxin VR-1	P0DL42	*D*. *siamensis*	4.79	1	4.84	1
**Snake venom nerve growth factor (svNGF)**			**2**.**11**	**1**	**2**.**13**	**1**
Venom nerve growth factor	P30894	*D*. *russelii*	2.11	1	2.13	1
**Snake venom 5′-nucleotidase (5′NUC)**			**0**.**82**	**3**	—	—
Snake venom 5′-nucleotidase	F8S0Z7	*C*. *adamanteus*	0.16	1	—	—
5′-nucleotidase	U3T7C6	*O*. *okinavensis*	0.17	2	—	—
Snake venom 5′-nucleotidase	CL3322.contig1_DrSL*	*D*. *russelii*	0.49	3	—	—
**Cysteine-rich secretory protein (CRiSP)**			**0**.**95**	**1**	—	—
Cysteine-rich venom protein ablomin	Q8JI40	*G*. *blomhoffii*	0.95	1	—	—
**Phosphodiesterase (PDE)**			**0.25**	**1**	**0.31**	**1**
phosphodiesterase 1	CL3655.contig2_DrSL*	*D*. *russelii*	0.25	1	0.31	1
**Aminopeptidase**			**0**.**35**	**2**	**0**.**15**	**2**
Xaa-Pro aminopeptidase 2	A0A0B8RNS9	*B*. *irregularis*	0.20	1	0.13	1
Xaa-Pro aminopeptidase 2-like	Unigene32033_DrSL*	*D*. *russelii*	0.15	2	0.02	2

* indicate venom protein identified based on tryptic peptides matched to sequence from in-house transcripts database. Mass spectrometric data and peptide sequences are available in Supplementary Information Files S2A and B.

*D*. *russelii*, *Daboia russelii; D*. *siamensis*, *Daboia siamensis; M*. *lebetina*, *Macrovipera lebetina; N*. *naja*, *Naja naja; Vipera berus berus*, *V*. *berus berus; C*. *rhodostoma*, *Calloselasma rhodostoma; C*. *adamanteus*, *Crotalus adamanteus; O*. *okinavensis*, *Ovophis okinavensis; G*. *blomhoffii*, *Gloydius blomhoffii; B*. *irregularis*, *Boiga irregularis*.

Kunitz-type serine protease inhibitors (KSPI) and phospholipases A_2_ (PLA_2_) are the two most abundantly expressed toxin families in the venoms. These two protein families together constitute 45–50% of the total venom proteins (Fig. 1). The toxin proteoforms detected, nonetheless, varied between the two venoms (Table 1). A total of 5 KSPI forms were identified in the Ds-Taiwan venom, while in the Ds-Guangxi venom there were only 3 KSPI forms. The proteoforms of PLA_2_ identified also varied between the two: in the Ds-Guangxi venom, the 6 PLA_2_ forms detected were distinct from 2 PLA_2_ proteoforms (RV-4 and RV-7) present in the Ds-Taiwan venom. Snaclec (snake venom C-type lectin/lectin-like protein) made up about 17% of protein bulk in both venoms. Snake venom serine proteases (SVSP) were slightly more abundant in the Ds-Taiwan venom (17.51%) compared with the Ds-Guangxi venom (13.61%), while the Ds-Guangxi venom showed a higher abundance of snake venom metalloproteinases (SVMP, 8.92%) than that of the Ds-Taiwan venom (5.86%). Besides, both venoms contained comparable abundances of 4 minor protein families (svVEGF, ~5%; svNGF, ~2%; PDE, ~0.3%; putative toxin aminopeptidase, ~0.3%) (Table 1). The peptide sequences and data of mass spectrometry are available in Supplementary Information files S2A and S2B.

### Antivenom protein concentrations

Table 2 shows the protein concentrations of four antivenoms determined using the bicinchoninic acid (BCA) protein assay kit, with bovine serum albumin as the standard for protein calibration.

**Table 2:** Protein concentrations of the four antivenoms used.

Antivenom	Protein concentration (mg/ml)
*Daboia siamensis* Monovalent Antivenom, Taiwan (DsMAV-Taiwan)	19.3 ± 0.5
*Daboia siamensis* Monovalent Antivenom, Thailand (DsMAV-Thai)	40.8 ± 0.3
*Gloydius brevicaudus* Monovalent Antivenom (GbMAV)	168.5 ± 0.7
*Deinagkistrodon acutus* Monovalent Antivenom (DaMAV)	181.1 ± 6.4

### Immunoreactivity of antivenoms to *D. siamensis* venoms

**Figure 2:**
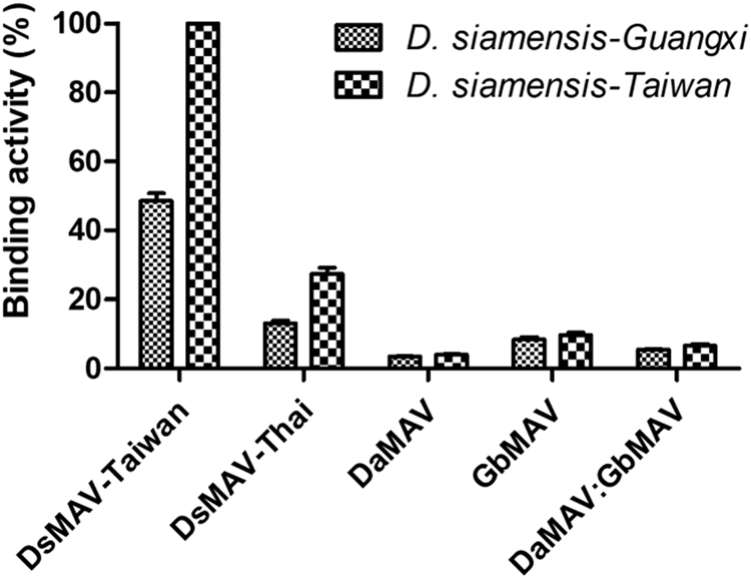
Immunological binding activity of antivenoms (DsMAV-Taiwan, DsMAV-Thai, DaMAV, GbMAV and a 1:1 mixture of DaMAV:GbMAV) toward the venom antigens of *Daboia siamensis* from Guangxi and Taiwan.

The four antivenoms (DsMAV-Taiwan, DsMAV-Thai, DaMAV and GbMAV) and an additional combination of DaMAV and GbMAV in a ratio of 1:1 were tested for their immunological binding activity toward Ds-Guangxi and Ds-Taiwan venoms (Fig. 2). The highest immunoreactivity (reflecting antigen binding activity) was observed in the reaction of DsMAV-Taiwan with Ds-Taiwan venom (Fig. 2). The Ds-Guangxi venom showed a lower immunoreactivity (approximately 50% lower) when reacting with the homologous antivenom from Taiwan. Both venoms also showed immunoreactivity for DsMAV-Thai but the relative magnitude of reactivity was apparently lower, found in the range of 15% (Ds-Guangxi) to 30% (Ds-Taiwan). The heterologous antivenoms (DaMAV, GbMAV and a combination of DaMAV:GbMAV in a ratio of 1:1) were generally low in immunoreactivity (<10%) to both Ds-Guangxi and Ds-Taiwan venoms.

### Procoagulant activity of *D. siamensis* venoms and antivenom neutralization

**Figure 3:**
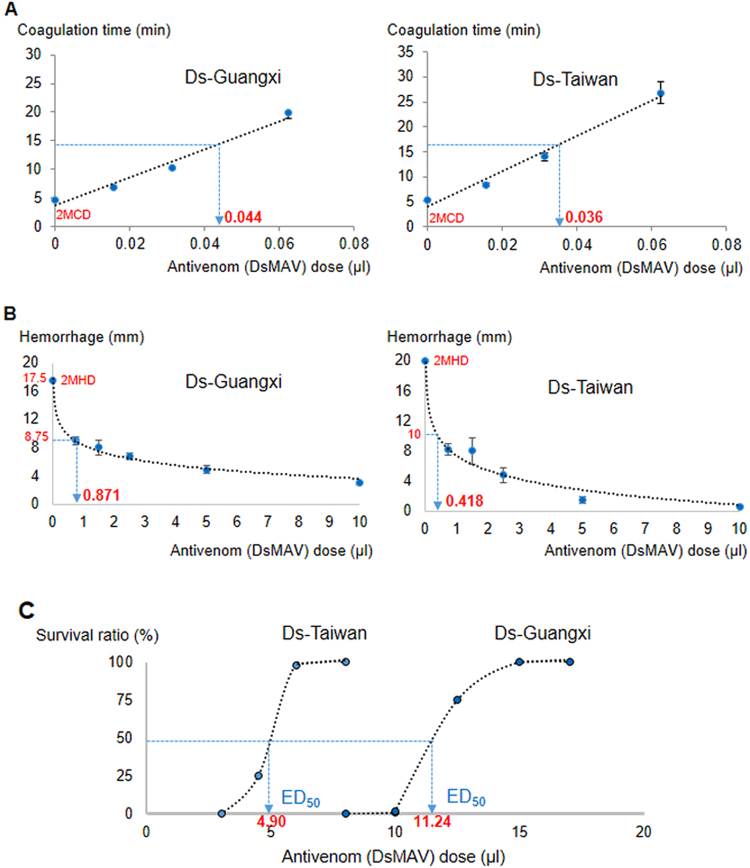
Efficacy of the Taiwan *Daboia siamensis* Monovalent Antivenom (DsMAV-Taiwan) in neutralizing the toxic effects of *D*. *siamensis* venoms from Guangxi and Taiwan. (**A**) Procoagulant effect; (**B**) Hemorrhagic effect. (**C**) Lethal effect.

Both *D*. *siamensis* venoms exhibited potent procoagulant effect with minimal coagulation dose (MCD) of 0.23 ± 0.06 µg/ml and 0.15 ± 0.04 µg/ml for Ds-Guangxi and Ds-Taiwan venoms, respectively (Table 3). DsMAV-Taiwan neutralized the procoagulant effect of Ds-Guangxi and Ds-Taiwan venoms in a dose-dependent manner (Fig. 3A), and the efficacy values of neutralization (defined as ED, effective dose) were comparable for both venoms (ED = ~1.4 to 2.0 mg venom neutralized per millilitre of antivenom, Table 3). The neutralization by GbMAV was extremely poor, at least 30-fold less effective compared with DsMAV-Taiwan. On the other hand, DaMAV was totally ineffective in neutralizing the procoagulant effect of Ds-Guangxi and Ds-Taiwan venoms. For comparison, the effective doses of the antivenoms were normalized by the respective protein concentrations (Table 3). The normalized effective dose (n-ED) for procoagulant effect of DsMAV-Taiwan (in mg/g, milligram of venom neutralized per gram of antivenom protein) was at least 250-fold higher than those of GbMAV and DaMAV.

**Table 3:** Procoagulant effect of *Daboia siamensis* venoms sourced from Guangxi and Taiwan and its neutralization by antivenoms.

*D*. *siamensis* venom	MCD^a^ (µg/ml)	Challenge dose (2MCD) (µg/ml)	DsMAV-Taiwan		GbMAV		DaMAV	
			ED^b^ (µl, mg/ml)	Normalized ED, n-ED^c^ (mg/g)	ED^b^ (µl, mg/ml)	Normalized ED, n-ED^c^ (mg/g)	ED^b^ (µl, mg/ml)	Normalized ED, n-ED^c^ (mg/g)
Guangxi	0.23 ± 0.06	0.46	0.044 ± 0.002, 2.03 ± 0.12	105.2	1.183 ± 0.017, 0.07 ± 0.00	0.4	>10, <0.01	<0.05
Taiwan	0.15 ± 0.04	0.30	0.036 ± 0.003, 1.41 ± 0.13	73.1	2.633 ± 0.088, 0.02 ± 0.00	0.1	>10, <0.01	<0.05

MCD: Minimal clotting dose; ED: Effective dose.

^a^Minimal clotting dose was defined as the dose of venom (µg/ml) required to cause clotting in 5 minutes.

^b^Effective dose was defined as the dose of antivenom capable of prolonging the clotting time of challenge dose to 3 times that of the control. ED was expressed in units of antivenom volume (µl) and venom amount per unit volume of antivenom (mg/ml).

^c^Normalized ED was derived from ED (mg/ml) by normalizing the antivenom volume by antivenom protein concentration.

### Hemorrhagic activity of *Daboia siamensis* venoms and antivenom neutralization

The Ds-Guangxi venom showed a lower minimal hemorrhagic dose (MHD) of 3.42 ± 0.12 µg/mouse compared with the Ds-Taiwan venom (MHD = 8.21 ± 0.31 µg/mouse). DsMAV-Taiwan neutralized the hemorrhagic effect of the venoms dose-dependently (Fig. 3B), but the neutralization was much more effective against the hemorrhagic effect of Ds-Taiwan venom than that of Ds-Guangxi venom based on the normalized median effective doses (n-ED_50_) (Table 4). In comparison with DsMAV-Taiwan, GbMAV and DaMAV were extremely weak in neutralizing the hemorrhagic effect induced by the *D*. *siamensis* venoms. DsMAV-Taiwan was at least 70–300 folds more effective in neutralizing the hemorrhagic effect.

**Table 4:** Hemorhagic effect of *Daboia siamensis* venoms sourced from Guangxi and Taiwan and its neutralization by antivenoms.

*D*. *siamensis* venom	MHD^a^ (µg/mouse)	Challenge dose (2MHD) (µg/mouse)	DsMAV-Taiwan		GbMAV		DaMAV	
			ED_50_^b^ (µl, mg/ml)	Normalized ED_50_, n-ED_50_^c^ (mg/g)	ED_50_^b^ (µl, mg/ml)	Normalized ED_50_, n-ED_50_^c^ (mg/g)	ED_50_^b^ (µl, mg/ml)	Normalized ED_50_, n-ED_50_^c^ (mg/g)
Guangxi	3.42 ± 0.12	6.84	0.871 ± 0.159, 7.85 ± 3.70	406.74	>5, <1	<6	>5, <1	<6
Taiwan	8.21 ± 0.31	16.42	0.418 ± 0.082, 39.28 ± 7.17	2035.35	>5, <1	<6	>5, <1	<6

MHD: Minimal hemorrhagic dose; ED_50_: Median effective dose.

^a^Minimal hemorrhagic dose was defined as the amount of venom (µg) required to induce a skin hemorrhagic lesion of 10 mm diameter.

^b^Median effective dose was defined as the dose of antivenom capable of reducing the venom hemorrhagic activity of 2MHD by 50%. ED_50_ was expressed in units of antivenom volume (µl) and venom amount per unit volume of antivenom (mg/ml).

^c^Normalized ED_50_ was derived from ED_50_ (mg/ml) by normalizing the antivenom volume by its protein concentration.

### Lethality of *D. siamensis* venoms and *in vivo* neutralization in mice

When administrated intravenously, Ds-Guangxi and Ds-Taiwan venoms had median lethal doses (LD_50_) of 0.18 µg/g and 0.09 µg/g, respectively (Table 5). DsMAV-Taiwan showed dose-dependent neutralization effects against the lethality of the venoms (constituting 5 LD_50_) (Fig. 3C). As the two venoms were different in their LD_50_ and the total amount of venom injected, the lethality neutralization was expressed in terms of neutralization potency (P) (Table 5). The neutralization of DsMAV-Taiwan was slightly more potent on Ds-Taiwan venom (P = 1.62 mg/ml, or n-P = 83.9 mg/g) than on Ds-Guangxi venom (P = 1.41 mg/ml, or normalized potency, n-P = 73.1 mg/g). On the other hand, GbMAV had a much lower potency of cross-neutralization against the venom lethality (P = 0.17 mg/ml or n-P = 1.0 mg/g), at least 70-fold lesser when comparing with the n-P values of DsMAV-Taiwan. DaMAV was totally ineffective to cross-neutralize the venom lethality at the maximal dose of antivenom (200 µl) administered intravenously into the mice.

**Table 5:** Lethality of *Daboia siamensis* venoms sourced from Guangxi and Taiwan and the efficacy of antivenoms in neutralizing the lethal effect.

*D*. *siamensis* venom	Challenge dose	*i*.*v*. LD_50_ (µg/g)^a^	ED_50_ (µl)^b^	ER_50_ (mg/ml)^c^	Potency, P (mg/ml)^d^	AV protein concentration (mg/ml)	Normalized P, n-P (mg/g)^e^
			**DsMAV-Taiwan**				
Guangxi	5	0.18 (0.12–0.27)	11.24	1.76 (1.17–2.64)	1.41	19.3 ± 0.5	73.1
Taiwan	5	0.09 (0.06–0.14)	4.90	2.02 (1.35–3.14)	1.62	19.3 ± 0.5	83.9
			**GbMAV**				
Guangxi	5	0.18 (0.12–0.27)	91.24	0.22 (0.14–0.33)	0.17	168.5 ± 0.7	1.0
			**DaMAV**				
Guangxi	5	0.18 (0.12–0.27)	N.E.	N.E.	N.E.	181.1 ± 6.4	N.E.

LD_50_: Median lethal dose; ED_50_: Median effective dose; ER_50_: Median effective ratio; MCD: Minimal clotting dose; ED: Effective dose; N.E.: Non-effective.

^a^Median lethal dose was defined as the dose of venom (µg/ml) at which 50% of mice were dead.

^b^Median effective dose was defined as the dose of antivenom (µl) at which 50% of mice survived.

^c^Median effective ratio was defined as the ratio of venom (mg) to the volume dose of antivenom (ml) at which 50% of mice survived.

^d^Potency, P was defined as the amount of venom (mg) completely neutralized by one ml of antivenom (ml).

^e^Normalized P, n-P was defined as the neutralization potency of the antivenom in mg venom/g antivenom protein.

## Discussion

Thorpe *et al*.^2^ suggested that the eastern Russell’s viper underwent an almost simultaneous rapid divergence 2–3 mybp. The Myanmese or Cambodian specimen is basal in the phylogenetic tree, while the Javan branch is sister to the geographically distant Chinese and Taiwanese branches^2^. This was likely to be at a time of mainland range expansion that enabled rapid overland colonization of the snake into the Taiwan once physically joined to the mainland of China over the Pleistocene^43^. Nonetheless, the clinical manifestations of snakebite envenoming often show marked geographical variations that are broadly unrelated to the phylogeny, and this phenomenon has been elucidated by venom proteomic and transcriptomic studies of a number of snake species^6,10,11,14,44^. Hence, the observed incongruence between the envenoming effects and the primary phylogenetic division of the Russell’s viper complex does not allow the prediction of Russell’s viper envenoming effects and antivenom responses in areas where these have not been studied directly^2,10,12,14^. In this context, *D*. *siamensis* venoms from the southern China and Taiwan were reported to cause hemotoxic envenomation^19–21^; however, knowledge on the venom composition was largely limited to isolated toxins of specimens from either the insular Taiwan^31,32,34,45^ or mainland China^46–48^. Using a comparative approach, this present work successfully unveiled the proteomes and toxic properties of the venom sourced from two locales (Guangxi and Taiwan). The findings revealed a greater number of protein families in the proteomes of the two *D*. *siamensis* venoms than that of Myanmese *D*. *siamensis* reported previously where only six protein families were identified (i.e. phospholipase A_2_, PLA_2_; C-type lectin/lectin-like protein, snaclecs; L-amino acid oxidase. LAAO; snake venom serine protease, SVSP; snake venom metalloproteinase, SVMP and snake venom vascular endothelial growth factor; svVEGF)^37^. The discrepancy observed could be probably due to different techniques used, as the current approach (integrating whole venom in-solution shotgun proteomics with recent database mining) might be a more sensitive venomic tool for studying the complexity and diversity of venom proteins^49–53^. The major protein families (Kunitz-type serine protease inhibitor or KSPI, PLA_2_, snaclec, SVMP and SVSP) expressed in both Ds-Guangxi and Ds-Taiwan venoms were essentially the same, suggesting that these paralogous proteins from specimens of the two locales were highly conserved with evolutionary significance. The variations in the proteoforms probably implied further molecular adaptation to different ecological niches.

The predominance of KSPI in both Ds-Guangxi and Ds-Taiwan venoms is in agreement with the high abundance of this non-enzymatic protein reported in the venom proteomes of the Russell’s viper from Pakistan (28.4%)^12^ and western India (32.5%)^14^. KSPI however was not reported in the venom proteome of the Myanmese *D*. *siamensis*^37^, although more recently this protein has been isolated from the Myanmese and Chinese *D*. *siamensis* venoms^29,30^. The KSPI proteoforms detected in the present study showed sequences matched to those reported previously for *D*. *siamensis* of Myanmar^30^, China^29^ and an unreported locale^54^. In general, KSPI are protease inhibitors with approximately 60–66 amino acids (~7 kDa) and are homologous to the conserved Kunitz motif present in the bovine pancreatic trypsin inhibitor^55^. Besides its serine protease inhibitory activity^29,30^ which could be important for venom protein storage in the venom glands, KSPI of Russell’s viper venom has also been shown to exhibit anticoagulant effect^56,57^. The pathophysiological role of KSPI in Ds-Guangxi and Ds-Taiwan venoms hence may be coagulopathy-related but the properties of the purified toxin await further investigation.

The high abundance of PLA_2_ in both *D*. *siamensis* venoms from Guangxi and Taiwan is in line with the finding of high content of PLA_2_ (~35%) in the Myanmese *D*. *siamensis* venom^31^. PLA_2_ is a dominant venom protein family in virtually all reported *D*. *russelii* venom proteomes, including those from Pakistan (32.8–63.8%)^12,13^, Western India (32.5%)^14^ and Sri Lanka (35%)^15^. However, the activities of the different PLA_2_ subtypes are diverse, and the effects can be ranging from no toxicity to high lethality^31,58,59^. The PLA_2_ detected in Ds-Guangxi and Ds-Taiwan venoms were apparently of different subtypes, implying that the PLA_2_ toxic activities could be diverse. In Ds-Taiwan venom, the presence of PLA_2_ RV-4 and RV-7 (in a ratio of 1:1) is consistent with the PLA_2_ isolated from the Taiwanese Russell’s viper venom reported earlier, where the PLA_2_ formed a heterodimeric complex that induced presynaptic neurotoxicity^60^. Putative neurotoxic PLA_2_ were also detected in Ds-Guangxi venom (daboiatoxin A, B chain and DsM-b1)^61,62^. However, neurotoxicity is mainly reported in envenomation by *D*. *russelii* in Sri Lanka and some parts of southern India^8,26,63,64^; it is not a commonly observed clinical feature in envenomation by the eastern Russell’s vipers in Southeast Asia^26^, Taiwan^21^ or China^17,65^. The neurotoxicity induced by *D*. *siamensis* PLA_2_ in laboratory animals probably reflects the complex interactions between toxins and the neurons of different specificity in animals^60^, where the natural prey such as rodents appear to be more susceptible to the PLA_2_-induced neurotoxicity than human beings are.

*D*. *siamensis*-envenomed patients in China and Taiwan often developed coagulopathy and bleeding diathesis with or without renal complication^17–21^. The hemotoxicity of *D*. *siamensis* venom is collectively caused by a number of toxins. Viperid PLA_2_, including a neutral PLA_2_ purified from *D*. *russelii* venom, were known to exhibit anticoagulant activity^66,67^. Other venom proteins detected in substantial amounts in Ds-Guangxi and Ds-Taiwan venoms were mainly hemotoxins that can induce consumptive coagulopathy and hemorrhage, such as snaclec, SVSP and SVMP.

Snaclecs (comprising C-type lectins and C-type lectin-like proteins of snake venom) are non-enzymatic toxins that can modulate thrombosis and hemostasis^68–70^. Various proteoforms of snaclec were detected in both venom proteomes, including dabocetin, a heterodimer consisting alpha and beta subunits that inhibit ristocetin-induced platelet aggregation^32^. Two RVV-X light chains which are homologous to snaclecs were also detected in the venoms. As part of the RVV-X metalloproteinase, these C-type lectin-like proteins were suggested to play a regulatory role in the calcium-dependant activation of factor X, probably through the recognition of specific sites of the zymogen factor X^68,71^.

Snake venom serine protease (SVSP) is another important protein family of viperid venoms that can cause venom-induced consumptive coagulopathy^38,72,73^. Several SVSP proteoforms were detected in the *D*. *siamensis* venom proteomes, one of which is Factor V activating enzyme (RVV-V), a serine protease that specifically activates Factor V (through cleavage at Arg1545-Ser1546 bond) to induce prothrombinase complex in a calcium-dependent manner^74^. The presence of alpha and beta fibrinogenases in the venoms also suggests that fibrinogenolytic activity may contribute to systemic coagulopathy in envenomation. Besides, the SVMP VLAIP-A detected in the Ds-Guangxi and Ds-Taiwan venoms is another hemotoxin known to induce fibrinogenolysis and coagulopathy^75^. More importantly, the Factor X activating enzyme (RVV-X), present in both *D*. *siamensis* venoms, is a potent procoagulant enzyme unique to Russell’s viper. RVV-X is a heterotrimeric metalloproteinase (93 kDa) composed of a heavy chain (from PIII-SVMP containing metalloproteinase, disintegrin-like and cysteine rich domains, 58 kDa) and two light chains of snaclecs (beta-chain, ~19 kDa and gamma-chain, ~16 kDa)^68,71,76^. In this study, the higher abundance of RVV-X in the Ds-Taiwan venom corroborated the stronger procoagulant effect of the venom on human citrated plasma. On the other hand, the hemorrhagic PIII-SVMP daborhagin-K was detected only in Ds-Guangxi venom; this PIII-SVMP was similar to the potent hemorrhagin SVMP purified from the Myanmese *D*. *siamensis* venom^34^. The substantial amount of PIII-SVMP detected in the venom proteomes hence supported the clinical presentation of hemorrhages in *D*. *siamensis* envenomation. Nevertheless, the distinctive presence of daborhagin-K in Ds-Guangxi venom correlated with the more potent hemorrhagic effect of the venom (shown in this study), and this venom property might be associated with the prominent local and systemic bleeding reported in Chinese *D*. *siamensis* envenoming^20^. Clinically, the potential renal complication (acute kidney injury) of Russell’s viper envenoming could be due to the nephrotoxic effect of SVMP and PLA_2_ mediated through cytotoxic activity or secondary to renal hypoperfusion in severe bleeding^26,77,78^.

In the current study, LAAO was detected only in Ds-Guangxi venom, consistent with the observation of a more intense protein band around 60 kDa on the SDS−PAGE under reducing conditions. The absence of LAAO in the proteome of Ds-Taiwan venom is puzzling as this enzyme is present in the venoms of most Viperidae and Elapidae^11,79–81^, including the Russell’s vipers of South Asia (Sri Lanka, India, Pakistan)^12–16^ and Myanmar^37^. In fact, the colors of the two venoms studied in the present study exhibited marked differences: the yellow coloration of Ds-Guangxi venom was likely due to the presence of flavin-containing LAAO, while the lyophilized Ds-Taiwan venom was white in color, implying the lack of this enzyme in the venom. The finding indicated that the amount of LAAO in Ds-Taiwan venom was low or negligible, a characteristic perhaps influenced by ecology and the condition of diet. LAAO may exhibit cytotoxicity and anti-microbial activity to facilitate prey digestion^82^, but its pathophysiological role in *D*. *siamensis* envenoming remains unclear.

The minor venom proteins (<10% of total venom proteins) i.e. snake venom vascular endothelial growth factor (svVEGF), nerve growth factor (svNGF), 5′nucleotidase (5′NUC), cysteine-rich secretory protein (CRiSP) and phosphodiesterase (PDE) may play a role in the predatory or digestive function of the venoms. Some of these venom components were known to induce hypotensive or proinflammatory effects, thereby facilitating the subduing of prey. For instance, svVEGF has been shown to increase capillary permeability^83^ and induces hypotensive effect^84^. Meanwhile, svNGF through the release of nitric oxide and/or histamine, 5′NUC through the release of purines (adenosine) and PDE through the alteration of extracellular levels of adenosine and other purines^85^, may contribute to the venom-induced hypotensive effect or venom spread in the prey. Furthermore, the release of adenosine may cause platelet aggregation inhibition, thereby worsening the hemostatic derangement in Russell’s viper envenomation^85^. Also, it has been shown that PDE could strongly inhibit ADP-induced platelet aggregation in human plasma, hence potentiating the hemotoxic effect of the venom^86^. On the other hand, CRiSP (detected in Ds-Guangxi venom) was similar to ablomin of *Gloydius blomhoffii*, a minor venom protein that targets L-type voltage-gated calcium channels (Ca_v_) and blocks smooth muscle contraction^87^. The toxicological property of this protein in *D*. *siamensis* venom remains to be further studied.

In mainland China, there are two widely distributed medically important pit vipers namely *D*. *acutus* (five-pace snake, also known as sharp-nosed pit viper) and *G*. *brevicaudus* (short-tail pit viper/Chinese mamushi). Specific antivenoms effective against these two pit vipers have been developed for clinical treatment^88–90^. These antivenoms for pit vipers (DaMAV for *D*. *acutus* and GbMAV for *G*. *brevicaudus*) were anecdotally reported to have been used as alternative antidote to treat *D*. *siamensis* envenomation in mainland China in the absence of the specific *D*. *siamensis* antivenom. In this study, the weak immunoreactivity of DaMAV and GbMAV against the *D*. *siamensis* venoms indicated that the two heterologous antivenoms had very limited binding activity toward the venom antigens of *D*. *siamensis*. This is likely due to the substantial differences in the compositions and antigenicity of venom proteins between *D*. *siamensis* and the two Chinese pit vipers. For instance, the PLA_2_ subtypes reported from the venoms of *G*. *brevicaudus* and *D*. *acutus* have amino acid sequences varied from those of *D*. *siamensis* in this study^91,92^. KSPI which formed the major component of *D*. *siamensis* venom have not been reported from the two pit viper venoms. Furthermore, the PIII-SVMP subtype was the main SVMP form expressed in both Ds-Guangxi and Ds-Taiwan venoms. However, in the Chinese *G*. *brevicaudus* venom, close to 65% of the total venom proteins were made up of a mixture of PII and PIII-SVMP^91^, while approximately only 10% of PI and PIII-SVMP was reported in the Taiwanese *D*. *acutus* venom^93^. The variability of protein families and proteoforms in these venoms imply that the toxins are likely diverse in their antigenicity. Compared with Ds-Taiwan venom, Ds-Guangxi venom was slightly less immunoreactive toward DsMAV-Taiwan, indicating that the antigenicity of some venom proteins varied between the two *D*. *siamensis*. On the other hand, the weak binding activity of DsMAV-Thai toward both Ds-Guangxi and Ds-Taiwan venom proteins implied that the Thai *D*. *siamensis* venom could be more diverse antigenically from the venoms of the geographically distant Ds-Guangxi and Ds-Taiwan.

The potent procoagulant and hemorrhagic effects of *D*. *siamensis* venoms correlated with the hemotoxic syndrome of *D*. *siamensis* envenomation in the region. In China, *D*. *siamensis* envenoming has been reported to cause severe bleeding including cerebral hemorrhage^20^; hence, it is essential to ensure that the antivenom used clinically is able to neutralize this hemorrhagic effect of the venom, besides neutralizing the procoagulant and lethal effects. The present study demonstrated that the heterologous DaMAV and GbMAV were rather ineffective in cross-neutralizing the hemotoxic (procoagulant and hemorrhagic) effects of Ds-Guangxi venom, even though GbMAV showed weak cross-neutralizing capability against the venom lethality when given at a very high dosage, judging from its low potency. The finding in this study therefore suggests that both DaMAV and GbMAV may not be the appropriate treatment of *D*. *siamensis* envenomation. On the other hand, Ds-Guangxi and Ds-Taiwan venoms shared conserved antigenicity of key toxins, thus enabling DsMAV-Taiwan to neutralize the procoagulant, hemorrhagic and lethal effects of Ds-Guangxi venom effectively.

## Conclusion

Shotgun proteomics showed that the principal toxins in the venoms of *D*. *siamensis* from Guangxi and Taiwan were comparable. The venom proteins within each protein family, however, varied between the two *D*. *siamensis* venoms. The subproteomic variation between the two could be reflective of ecological adaptation to diet since the mainland and the insular populations are physically long separated by the Taiwan Strait. However, the venom divergence could also be the result of random fixation of neutral alleles, with sequence differences that relate to neither dietary nor ecological factors on fitness. Both *D. siamensis* venoms exhibited potent hemotoxicity and lethality. Ds-Guangxi venom was comparatively more potent in hemorrhagic effect while Ds-Taiwan venom was a stronger procoagulant. Immunoreactivity and neutralization studies further revealed that the antigenicity of the major toxins of *D*. *siamensis* were relatively well conserved across the Strait. The study further showed that the heterologous DaMAV and GbMAV did not confer effective cross-neutralization against the venom hemotoxicity and lethality of Ds-Guangxi.

## Materials and Methods

### Chemicals and materials

All chemicals and reagents used in the study were of analytical grade. Ammonium bicarbonate, dithiothreitol (DTT) and iodoacetamide were purchased from Sigma-Aldrich (USA). Mass spectrometry sequencing grade of trypsin proteases, and HPLC grade solvents used in the studies were purchased from Thermo Scientific™ Pierce™ (USA). Millipore ZipTip^®^ C_18_ Pipette Tips were purchased from Merck (USA).

### Venoms and antivenoms

The venom of *Daboia siamensis* of the mainland of China was a pooled sample milked from several adult specimens (n > 10, average venom yield 30–59 mg) captured in Guangxi. The venom of *D*. *siamensis* from the insular Taiwan was a gift from Professor Inn-Ho Tsai from the National Taiwan University. The venoms were stored as lyophilized samples at −20 °C until use. Four different antivenoms were used in the present study: (a) Taiwanese *D*. *siamensis* Monovalent Snake Antivenom (DsMAV-Taiwan, neutralization efficacy not indicated in product sheet, lyophilized; batch no. FR10301; expiry date: Oct 31^st^, 2019, product of Taiwan Central for Disease Control in Taipei); (b) Thai *D*. *siamensis* Monovalent Snake Antivenom (DsMAV-Thai, 0.6 mg venom neutralized/ml of antivenom, lyophilized; batch no. WR00212; expiry date: Nov 19^th^, 2017, product of Queen Saovabha Memorial Institute in Bangkok). Both antivenoms (a) and (b) are purified F(ab)’_2_ obtained from sera of horses hyperimmunized against the venom of *D*. *siamensis* of the respective geographical origin. (c) *Gloydius brevicaudus* (short-tailed mamushi) Monovalent Snake Antivenom (GbMAV, contains 6000 IU/vial of 10 ml, lyophilized; batch no. 20141001; expiry date: Oct 30^th^, 2017); (d) *Deinagkistrodon acutus* (sharp-nosed pit viper) Monovalent Snake Antivenom (DaMAV, contains 2000 IU/vial of 10 ml, lyophilized; batch no. 20140501; expiry date: May 26^th^, 2017). Both (c) and (d) are products from Shanghai Serum Bio-Technology Co., Ltd.in Shanghai, and are purified F(ab)’_2_ obtained from sera of horses hyperimmunized against the venom of *G*. *brevicaudus* and *D*. *acutus* respectively. All antivenoms were used before expiry.

### Animals

Albino mice (ICR strain, 20–30 g) were supplied by the Animal Experimental Unit (AEU), Faculty of Medicine, University of Malaya. The animals were handled according to the Council for International Organization of Medical Sciences (CIOMS) guideline on animal experimentation^94^. All methods were carried out in accordance with the guidelines and regulations approved by the Institutional Animal Care and Use Committee (IACUC) of University of Malaya (Protocol approval number: 2014-09-11/PHAR/R/TCH).

### Estimation of antivenom protein concentration

Protein concentrations of antivenoms (DsMAV-Taiwan, DsMAV-Thai, GbMAV and DaMAV) were determined using Thermo Scientific™ Pierce™ BCA (bicinchoninic acid) protein assay kit with bovine serum albumin as protein standard calibration. The protein concentrations were expressed as means ± S.E.M. of triplicates.

### Whole venom in-solution tryptic digestion and protein identification by tandem mass spectrometry (nano-ESI-LCMS/MS)

Whole venom in-solution tryptic digestion was carried out in three technical replicates for each of the venom. Twenty micrograms for each sample of *D*. *siamensis* venoms (Ds-Guangxi and Ds-Taiwan) were subjected to reduction with DTT, alkylation with iodoacetamide, and digested in-solution with mass-spectrometry grade trypsin proteases as described previously^49^. Briefly, the digested peptides eluates were reconstituted in 7 µl of 0.1% formic acid in water. Peptides separation were performed by 1260 Infinity Nanoflow LC system (Agilent, Santa Clara, CA, USA) connected to Accurate-Mass Q-TOF 6550 series with a nano electrospray ionization source. The eluate was subjected to HPLC Large-Capacity Chip Column Zorbax 300-SB-C18 (160 nl enrichment column, 75 µm × 150 mm analytical column and 5 µm particles) (Agilent, Santa Clara, CA, USA). Injection volume was adjusted to 1 µl per sample, using a flow rate of 0.4 µl/min, with linear gradient of 5–70% of solvent B (0.1% formic acid in 100% acetonitrile). Drying gas flow was 11 L/min and drying gas temperature was 290 °C. Fragmentor voltage was 175 V and the capillary voltage was set to 1800 V. Mass spectra was acquired using Mass Hunter acquisition software (Agilent, Santa Clara, CA, USA) in a MS/MS mode with an MS scan range of 200–3000 m/z and MS/MS scan range of 50–3200 m/z. Data were extracted with MH+ mass range between 50 and 3200 Da and processed with Agilent Spectrum Mill MS Proteomics Workbench software packages version B.04.00 against merged database incorporating both non-redundant NCBI databases of Serpentes (taxid: 8570) and in-house transcripts database. Carbamidomethylation was specified as a fixed modification and oxidized methionine as a variable modification. The identified proteins or peptides were validated with the following filters: protein score > 20, peptide score > 10 and scored peak intensity (SPI) >70%. Identified proteins were filtered to achieve False discovery rate (FDR) <1% for the peptide-spectrum matches. The proteins identified were classified as toxins or non-toxins according to their putative functions. The abundance of individual venom toxin was estimated based on its mean spectral intensity (MSI) relative to the total MSI of all proteins identified through the in-solution mass spectrometry^49^.

### Sodium dodecyl sulphate-polyacrylamide gel electrophoresis (SDS-PAGE)

SDS-polyacrylamide gel electrophoresis (SDS-PAGE) was conducted according to method of Laemmli^95^ calibrated with the Thermo Scientific Spectra Multicolor Broad Range Protein Ladder (10–260 kDa). The venoms of both *D*. *siamensis* (Ds-Guangxi and Ds-Taiwan) was loaded onto a 15% gel and the electrophoresis was performed under reducing condition at 80 V for 2.5 h. Proteins were stained with Coomassie Brilliant Blue R-250 for gel visualization.

### Immunological binding assay

Immunological binding activities between venom antigens and antivenoms were examined with an indirect enzyme-linked immunosorbent assay (ELISA). The immunoplate wells were precoated with 10 ng of venom antigens at 4 °C overnight. The plate was then flicked dry and rinsed four times with phosphate-buffered saline with 0.5% Tween®20 (PBST). Antivenoms were prepared at a protein concentration of 10 mg/ml each, and 100 µl appropriately diluted antivenom (1:3000) was added to each antigen-coated well, followed by incubation for 1 h at room temperature. After washing the plate four times with PBST, 100 µl of appropriately diluted horseradish peroxidase-conjugated antihorse-IgG (Jackson ImmunoResearch Inc., USA) in PBST (1:8000) was added to the well and incubated for another hour at room temperature. The excess components were removed by washing four times with PBST. A hundred microliters of freshly prepared substrate solution (0.5 mg/mL o-phenylenediamine and 0.006% hydrogen peroxide in 0.1 M citrate-phosphate buffer, pH 5.0) was added per well. The enzymatic reaction was allowed to take place in the dark for 30 min at room temperature. The reaction was subsequently terminated by adding 50 µL of 12.5% sulphuric acid, and the absorbance at 492 nm was read using Tecan Infinite M1000 Pro plate reader (Tecan Laboratories, Switzerland). Immunological binding activity was expressed as percentage of relative absorbance (highest binding activity set as 100%) between two comparing antivenoms in immunological binding toward the both *D*. *siamensis* venoms (Ds-Guangxi and Ds-Taiwan)^96^. Values were means of triplicates ± S.E.M., and the significance of difference was analyzed using unpaired t-test with *p* value <0.05.

### Venom procoagulant activity and antivenom neutralization

Procoagulant effect of the venom was determined by adding 100 µl of citrated human plasma (containing 40 µl of 0.4 M CaCl_2_/ml) to 100 µl of *D*. *siamensis* venoms of various concentrations in saline at 37 °C according to a modified turbidimetric method^96,97^. The absorbance at 405 nm was monitored every 30 s over 30 min. This produced a plot of absorbance versus time (min) with an initial lag time at which absorbance began to increase drastically due to cloudiness of clot formation, followed by a hyperbolic curve to a plateau. The clotting time was determined as the time when the absorbance became 0.02 U greater than the mean of the first two absorbance measurements. The minimal clotting dose (MCD) was defined as the dose of venom that induces coagulation in 5 min.

In neutralization study, venom concentration equivalent to 2 times minimal coagulation dose (2 MCD) was pre-incubated with various doses of the antivenoms (DsMAV-Taiwan, GbMAV and DaMAV) at 37 °C for 30 min before the addition of 100 µl citrated human plasma. The determination of clotting time in the neutralization assay was performed as described above. The effective dose (ED) was defined as the dose of antivenom that prolonged the clotting time of human plasma 3 times that of the control (2 MCD, without antivenom).

The effective dose (ED) was calculated using the following formulation:

1 $${\rm{E}}{\rm{f}}{\rm{f}}{\rm{e}}{\rm{c}}{\rm{t}}{\rm{i}}{\rm{v}}{\rm{e}}\,{\rm{d}}{\rm{o}}{\rm{s}}{\rm{e}}\,({\rm{E}}{\rm{D}},{\rm{m}}{\rm{g}}/{\rm{m}}{\rm{l}})=\frac{2\,{\rm{t}}{\rm{i}}{\rm{m}}{\rm{e}}{\rm{s}}\,{\rm{m}}{\rm{i}}{\rm{n}}{\rm{i}}{\rm{m}}{\rm{a}}{\rm{l}}\,{\rm{c}}{\rm{o}}{\rm{a}}{\rm{g}}{\rm{u}}{\rm{l}}{\rm{a}}{\rm{t}}{\rm{i}}{\rm{o}}{\rm{n}}\,{\rm{d}}{\rm{o}}{\rm{s}}{\rm{e}}\,(2\,{\rm{M}}{\rm{C}}{\rm{D}},{\rm{m}}{\rm{g}})}{{\rm{D}}{\rm{o}}{\rm{s}}{\rm{e}}\,{\rm{o}}{\rm{f}}\,{\rm{a}}{\rm{n}}{\rm{t}}{\rm{i}}{\rm{v}}{\rm{e}}{\rm{n}}{\rm{o}}{\rm{m}}\,{\rm{t}}{\rm{h}}{\rm{a}}{\rm{t}}\,{\rm{p}}{\rm{r}}{\rm{o}}{\rm{l}}{\rm{o}}{\rm{n}}{\rm{g}}{\rm{e}}{\rm{d}}\,{\rm{t}}{\rm{h}}{\rm{e}}\,{\rm{c}}{\rm{l}}{\rm{o}}{\rm{t}}{\rm{t}}{\rm{i}}{\rm{n}}{\rm{g}}\,{\rm{t}}{\rm{i}}{\rm{m}}{\rm{e}}\,3\,{\rm{t}}{\rm{i}}{\rm{m}}{\rm{e}}{\rm{s}}\,{\rm{t}}{\rm{h}}{\rm{a}}{\rm{t}}\,{\rm{o}}{\rm{f}}\,{\rm{t}}{\rm{h}}{\rm{e}}\,{\rm{c}}{\rm{o}}{\rm{n}}{\rm{t}}{\rm{r}}{\rm{o}}{\rm{l}}\,({\rm{m}}{\rm{l}})}$$

For comparative purpose, ED values of antivenoms were normalized (n-ED) by their respective protein amount and expressed as milligram of venom neutralized per gram of antivenom protein (mg/g).

### Hemorrhagic activity of *D. siamensis* venom and antivenom neutralization

Hemorrhagic effect was assessed by intradermal venom injection into the dorsal skin of ICR mice (20–25 g, n = 3) as described by Gutiérrez *et al*.^98^. The animals were euthanized with urethane 90 min after venom exposure and the skins were removed. Minimal hemorrhagic dose (MHD) was defined as the amount of venom that induces a skin hemorrhagic lesion of 10 mm diameter. For neutralization assays, various doses of antivenom (DsMAV-Taiwan, GbMAV and DaMAV) were pre-incubated with a constant amount of venom challenge dose (2MHD) at 37 °C for 30 min prior to intradermal injection into the animals. The neutralization of hemorrhagic effects was expressed as median effective dose (ED_50_), defined as the amount of reconstituted antivenom in µl or the ratio of mg venom/ml reconstituted antivenom in which the venom activity was reduced by 50%.

The median effective dose (ED_50_) was calculated using the following formulation:

2 $${\rm{M}}{\rm{e}}{\rm{d}}{\rm{i}}{\rm{a}}{\rm{n}}\,{\rm{e}}{\rm{f}}{\rm{f}}{\rm{e}}{\rm{c}}{\rm{t}}{\rm{i}}{\rm{v}}{\rm{e}}\,{\rm{d}}{\rm{o}}{\rm{s}}{\rm{e}}\,({{\rm{E}}{\rm{D}}}_{50},{\rm{m}}{\rm{g}}/{\rm{m}}{\rm{l}})=\frac{2\,{\rm{t}}{\rm{i}}{\rm{m}}{\rm{e}}{\rm{s}}\,{\rm{m}}{\rm{i}}{\rm{n}}{\rm{i}}{\rm{m}}{\rm{a}}{\rm{l}}\,{\rm{h}}{\rm{e}}{\rm{m}}{\rm{o}}{\rm{r}}{\rm{h}}{\rm{a}}{\rm{g}}{\rm{i}}{\rm{c}}\,{\rm{d}}{\rm{o}}{\rm{s}}{\rm{e}}\,(2\,{\rm{M}}{\rm{H}}{\rm{D}},{\rm{m}}{\rm{g}})}{{\rm{D}}{\rm{o}}{\rm{s}}{\rm{e}}\,{\rm{o}}{\rm{f}}\,{\rm{a}}{\rm{n}}{\rm{t}}{\rm{i}}{\rm{v}}{\rm{e}}{\rm{n}}{\rm{o}}{\rm{m}}\,{\rm{i}}{\rm{n}}\,{\rm{w}}{\rm{h}}{\rm{i}}{\rm{c}}{\rm{h}}\,{\rm{t}}{\rm{h}}{\rm{e}}\,{\rm{v}}{\rm{e}}{\rm{n}}{\rm{o}}{\rm{m}}\,{\rm{a}}{\rm{c}}{\rm{t}}{\rm{i}}{\rm{v}}{\rm{i}}{\rm{t}}{\rm{y}}\,{\rm{w}}{\rm{a}}{\rm{s}}\,{\rm{r}}{\rm{e}}{\rm{d}}{\rm{u}}{\rm{c}}{\rm{e}}{\rm{d}}\,{\rm{b}}{\rm{y}}\,50{\rm{ \% }}\,({\rm{m}}{\rm{l}})}$$

For comparative purpose, ED_50_ values of antivenoms were normalized (n-ED_50_) by their respective protein amount and expressed as milligram of venom neutralized per gram of antivenom protein (mg/g).

### Determination of venom lethality and neutralization by antivenom

Median lethal doses (LD_50_) of venoms were determined by intravenous injection (via caudal veins) into ICR mice (n = 4 per dose, 20–25 g). The survival ratio was recorded after 48 h. In antivenom neutralization assay, pre-incubation of venom and antivenom was conducted as described by Tan *et al*.^11^. A challenge dose at 5 times LD_50_ of the venom dissolved in normal saline was pre-incubated with various dilutions of antivenoms (DsMAV-Taiwan, GbMAV and DaMAV) at 37 °C for 30 min and the mixture was injected intravenously into the mice (n = 4 per dose, 20–25 g). The mice were allowed free access to food and water ad libitum, and the ratio of survival was recorded at 48 h post injection. Neutralizing capacity was expressed as ED_50_, defined as the amount of reconstituted antivenom that gives 50% survival in the venom-challenged animals. These parameters were calculated according to the Probit analysis method^99^ using BioStat 2009 analysis software (AnalystSoft Inc., Canada). Neutralization capacity was also expressed in term of ‘neutralization potency’ (P, defined as the amount of venom in milligram neutralized completely by a unit volume of antivenom in millilitre, mg/ml)^96,100^. The neutralization potency (P) is a more direct indicator of antivenom neutralizing capacity, and is theoretically unaffected by the number of LD_50_ in the challenge dose. For comparative purpose, P values of antivenoms were normalized (n-P) by their respective protein amount and expressed as milligram of venom neutralized per gram of antivenom protein (mg/g).

## Electronic supplementary material

Supplementary File S1

Supplementary File S2A

Supplementary File S2B
