# Supplementary material for: Venom proteomics and antivenom neutralization for the Chinese eastern Russell’s viper, Daboia siamensis from Guangxi and Taiwan
Source: Sci Rep. 2018 Jun 4;8:8545. doi: 10.1038/s41598-018-25955-y (PMC5986800; doi:10.1038/s41598-018-25955-y)
Supplement: Supplementary file 1 — Supplementary File S1 [file 41598_2018_25955_MOESM1_ESM.pdf]

Venom proteomics and antivenom neutralization for the Chinese Eastern Russell's viper, *Daboia siamensis* from Guangxi and Taiwan

[a]Kae Yi Tan, [b*]Choo Hock Tan, [a]Nget Hong Tan

[a]Department of Molecular Medicine, Faculty of Medicine, University of Malaya, Kuala Lumpur, Malaysia.

[b]Department of Pharmacology, Faculty of Medicine, University of Malaya, Kuala Lumpur, Malaysia.

Supplementary File S1. Full gel image for the SDS PAGE of the Guangxi and Taiwan *Daboia siamensis* venoms. (Ds-Guangxi – *D. siamensis* of Guangxi; Ds-Taiwan – *D. siamensis* of Taiwan)

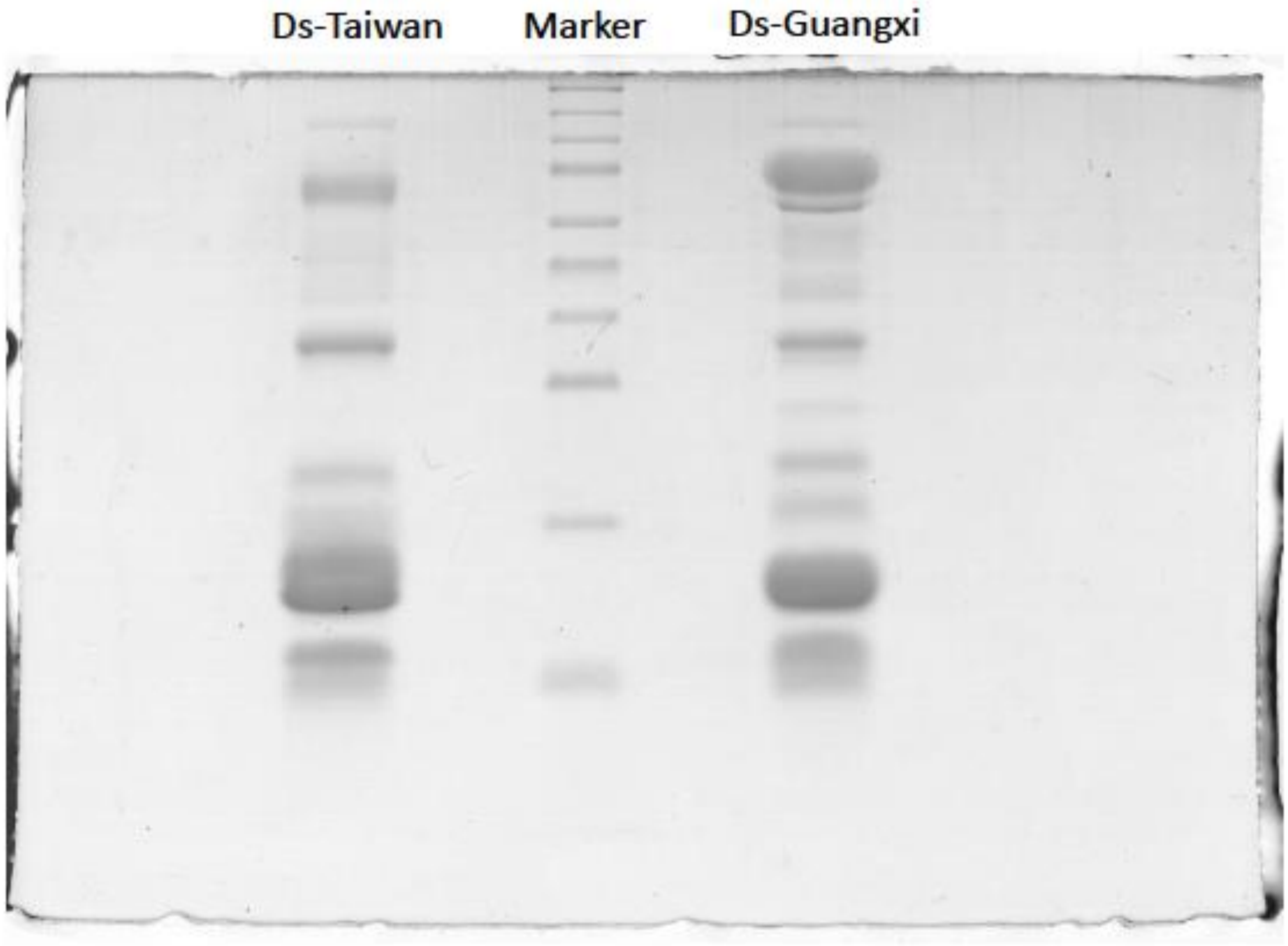

Ds-Taiwan
Marker
Ds-Guangxi
