# Supplementary material for: Venom proteomics and antivenom neutralization for the Chinese eastern Russell’s viper, Daboia siamensis from Guangxi and Taiwan
Source: Sci Rep. 2018 Jun 4;8:8545. doi: 10.1038/s41598-018-25955-y (PMC5986800; doi:10.1038/s41598-018-25955-y)
Supplement: Supplementary file 2 — Supplementary File S2A [file 41598_2018_25955_MOESM2_ESM.pdf]

Venom proteomics and antivenom neutralization for the Chinese Eastern Russell's viper, *Daboia siamensis* from Guangxi and Taiwan

[a]Kae Yi Tan, [b*]Choo Hock Tan, [a]Nget Hong Tan

[a]Department of Molecular Medicine, Faculty of Medicine, University of Malaya, Kuala Lumpur, Malaysia.

[b]Department of Pharmacology, Faculty of Medicine, University of Malaya, Kuala Lumpur, Malaysia.

Supplementary File S2A. LCMS-MS data collection for the Guangxi *Daboia siamensis* venom.

Daboia siamensis Guangxi venom 1

Group	Subgroup	Spectra	Distinct Peptides	Distinct Summed MS/MS Search Score	% AA Coverage	Mean Protein Spectral Intensity	Protein MW (Da)	Protein pI	Species	Database Accession	Protein Name	z	Score	Fwd-Rev Score	SPI (%)	Spectrum Intensity	Sequence	RT (min)	Peak Width (sec)	Averaging Chi Squared	m/z Measured (Da)	Peptide pI	Relative abundance (n=1)	Relative abundance (% overall)
1	1.1	16	12	217.59	26.0	1.40E+06	71721.7	5.95	*Daboia russelii*	B8K1W0	Zinc metalloproteinase-disintegrin-like daborhagin-K	4	14.73	14.73	72.60	3.15E+05	(R)AGTECRPARDECDKAEQCTGR(S)	3.97	2.68	0.97	617.5201	4.94	4.18%	1.39%
												2	26.05	26.05	87.80	4.49E+05	(R)GEECDCGSPENCRDPCCDAASCK(L)	4.32	0.00	0.86	1367.4733	4.02		
												3	16.66	16.66	84.80	2.85E+06	(K)LHSWVECESGK(C)	4.88	5.40	0.97	444.5418	5.40		
												2	20.12	20.12	77.30	2.66E+06	(K)LHSWVECESGK(C)	4.88	5.40	0.98	666.3088	5.40		
												2	16.41	9.19	93.40	4.66E+05	(K)YKNDLTAIR(T)	4.88	5.40	1.00	547.3046	8.59		
												4	16.11	8.40	92.70	3.24E+05	(R)RFLTEHNPECIINPPLR(T)	6.52	5.53	0.97	527.2798	6.76		
												2	20.99	10.60	95.90	6.01E+06	(R)TDIVSPPACGNELLER(G)	6.70	10.81	0.98	885.9427	4.14		
												2	23.20	17.23	98.50	2.53E+06	(R)FLTEHNPECIINPPLR(T)	6.95	10.81	1.00	975.5029	5.40		
												4	13.15	13.15	71.60	8.88E+04	(R)FLTEHNPECIINPPLR(T)	6.93	10.81	0.92	488.2542	5.40		
												2	16.17	16.17	90.80	1.91E+05	(K)VCSNGQCVDLNIAY(-)	7.73	0.00	1.00	806.8617	3.80		
												2	14.94	14.94	88.60	3.73E+06	(K)SPGNQIPCLPYYIPSDENK(G)	7.68	5.40	0.99	1096.5241	4.37		
												3	14.28	9.65	97.70	1.27E+06	(K)SPGNQIPCLPYYIPSDENK(G)	7.68	5.40	1.00	731.3513	4.37		
												3	14.06	7.89	88.00	8.27E+04	(R)VPLVGLEIWK(N)	8.90	10.81	0.99	385.2379	5.97		
												2	15.96	8.91	71.40	7.19E+04	(R)TWVFELVNTINEIFK(Y)	12.87	0.25	0.99	926.9979	4.53		
												3	15.74	5.63	78.30	1.41E+06	(R)TWVFELVNTINEIFKYLYIR(V)	14.40	16.83	0.99	854.4668	5.81		
												2	18.85	18.85	79.80	2.82E+04	(R)TWVFELVNTINEIFKYLYIR(V)	14.45	1.92	0.81	1281.1915	5.81		
2	2.1	14	10	175.41	37.1	7.58E+05	46713.1	7.90	*Daboia siamensis*	Q4F867	L-amino-acid oxidase	2	19.97	11.86	93.00	7.22E+05	(K)SAGQLYQESLGK(A)	5.47	5.40	0.98	640.8296	5.72	2.25%	0.75%
												2	14.41	14.41	85.50	3.35E+05	(K)IFLTCTK(K)	5.48	0.00	1.00	441.7423	8.75		
												3	11.27	4.22	81.30	8.90E+04	(K)KFWEDDGIQGGK(S)	5.58	0.00	0.97	460.5592	4.56		
												3	12.08	4.07	86.40	2.67E+05	(R)ITFKPPLPPK(K)	6.28	21.74	1.00	379.8994	10.00		
												2	21.27	15.53	96.10	2.19E+05	(K)DLQTFCYPSIIQK(W)	7.73	5.40	0.99	806.9071	5.83		
												2	19.48	19.48	87.10	7.48E+04	(R)FDEIVGGMDQLPTSMYR(A)	8.12	5.53	0.98	979.9551	4.03		
												3	14.71	3.93	84.60	1.02E+06	(R)IFFAGEYTANAHGWIDSTIK(S)	9.37	0.00	0.99	747.7060	5.32		
												2	22.61	14.46	97.90	6.81E+05	(K)LNEFVQETENGWYFIK(N)	9.02	10.81	0.93	1008.9905	4.25		
												3	18.87	14.06	88.70	2.28E+05	(K)LNEFVQETENGWYFIK(N)	9.02	10.81	0.94	672.9974	4.25		
												3	19.89	19.89	88.40	3.86E+05	(K)YAMGAITTFTPYQFQHFSEALTAPVGR(I)	9.37	0.00	0.99	1002.1647	6.75		
												3	14.71	4.79	82.70	9.98E+05	(R)IFFAGEYTANAHGWIDSTIK(S)	11.73	176.33	0.96	747.7052	5.32		
												3	18.07	10.90	88.90	3.41E+06	(K)NLLLETVDYVIVCTTSR(A)	11.32	15.72	1.00	666.0213	4.37		
												2	19.72	14.69	87.80	1.81E+06	(K)NLLLETVDYVIVCTTSR(A)	11.32	15.72	0.99	998.5271	4.37		
												3	15.58	10.32	79.80	3.78E+05	(K)NLLLETVDYVIVCTTSR(A)	12.38	31.19	0.94	666.0192	4.37		
2	2.2	13	10	173.61	29.1	4.32E+05	57286.2	8.82	*Daboia russelii*	G8XQX1	L-amino-acid oxidase	3	13.86	7.85	73.20	4.77E+05	(K)VTVLEASERPGGR(V)	4.87	5.40	0.98	457.5853	6.11	1.28%	0.43%
												2	17.92	11.61	93.70	1.16E+05	(K)VTVLEASERPGGR(V)	4.87	5.40	0.96	685.8749	6.11		
												2	19.97	11.86	93.00	7.22E+05	(K)SAGQLYQESLGK(A)	5.47	5.40	0.98	640.8296	5.72		
												2	14.41	14.41	85.50	3.35E+05	(K)IFLTCTK(K)	5.48	0.00	1.00	441.7423	8.75		
												3	11.27	4.22	81.30	8.90E+04	(K)KFWEDDGIQGGK(S)	5.58	0.00	0.97	460.5592	4.56		
												3	12.08	4.07	86.40	2.67E+05	(R)ITFKPPLPPK(K)	6.28	21.74	1.00	379.8994	10.00		
												2	21.27	15.53	96.10	2.19E+05	(K)DLQTFCYPSIIQK(W)	7.73	5.40	0.99	806.9071	5.83		
												2	19.48	19.48	87.10	7.48E+04	(R)FDEIVGGMDQLPTSMYR(A)	8.12	5.53	0.98	979.9551	4.03		
												3	14.71	3.93	84.60	1.02E+06	(R)IFFAGEYTANAHGWIDSTIK(S)	9.37	0.00	0.99	747.7060	5.32		
												2	22.61	14.46	97.90	6.81E+05	(K)LNEFVQETENGWYFIK(N)	9.02	10.81	0.93	1008.9905	4.25		
												3	18.87	14.06	88.70	2.28E+05	(K)LNEFVQETENGWYFIK(N)	9.02	10.81	0.94	672.9974	4.25		
												3	19.89	19.89	88.40	3.86E+05	(K)YAMGAITTFTPYQFQHFSEALTAPVGR(I)	9.37	0.00	0.99	1002.1647	6.75		
												3	14.71	4.79	82.70	9.98E+05	(R)IFFAGEYTANAHGWIDSTIK(S)	11.73	176.33	0.96	747.7052	5.32		
2	2.3	4	2	31.01	48.8	7.00E+05	10351.4	5.10	*Vipera berus berus*	P0C2D7	L-amino-acid oxidase	3	14.71	3.93	84.60	1.02E+06	(K)IFFAGEYTANAHGWIDSTIK(-)	9.37	0.00	0.99	747.7060	5.32	2.08%	0.69%
												4	11.48	11.48	90.80	4.86E+05	(-)ADDKNPLEECFREDDYEEFLEIAK(N)	8.63	10.81	0.99	744.5925	3.96		
												3	16.30	7.72	84.80	2.97E+05	(-)ADDKNPLEECFREDDYEEFLEIAK(N)	8.63	5.40	1.00	992.4515	3.96		
												3	14.71	4.79	82.70	9.98E+05	(K)IFFAGEYTANAHGWIDSTIK(-)	11.73	176.33	0.96	747.7052	5.32		
3	3.1	9	7	134.54	43.3	7.22E+05	18523.6	6.31	*Daboia siamensis*	Q4PRC6	Snaclec 7	2	17.97	10.99	77.70	1.29E+06	(K)TTDNQWLR(W)	5.40	0.00	1.00	517.2591	5.50	2.15%	0.72%
												2	19.93	15.70	97.30	6.09E+05	(R)SSEEMDFVIR(M)	6.92	10.81	1.00	606.7832	4.14		
												2	26.67	26.67	92.30	9.19E+05	(K)FCNEQVNGGYLVSFR(S)	7.28	0.00	1.00	895.4230	6.00		
												3	17.30	17.30	85.00	1.24E+05	(K)FCNEQVNGGYLVSFR(S)	7.28	0.00	0.97	597.2847	6.00		
												2	13.03	13.03	74.00	1.60E+06	(R)MTFPIFR(F)	8.23	5.53	1.00	456.2444	9.50		
												2	23.25	23.25	92.30	4.27E+05	(K)QDCLSDWSFYEGYCYK(V)	8.20	5.53	1.00	1060.9237	4.03		
												3	15.45	15.45	78.70	1.14E+05	(K)QDCLSDWSFYEGYCYK(V)	8.20	5.53	1.00	707.6167	4.03		
												2	17.56	17.56	88.20	1.35E+06	(R)FDFFWIGLR(D)	10.47	10.81	0.98	600.8153	5.84		
												3	16.13	16.13	81.70	6.55E+04	(R)MTFPIFRFDFFWIGLR(D)	11.93	6.97	0.99	698.3677	9.35		
3	3.2	7	4	76.05	50.6	4.98E+05	17529.7	6.58	*Daboia siamensis*	Q4PRC8	Snaclec 5	4	11.57	11.57	72.00	1.53E+06	(K)NHWSHMDCSSTHNFVCK(F)	4.95	0.00	0.97	537.4732	7.02	1.48%	0.49%
												3	16.15	16.15	83.30	7.47E+05	(K)NHWSHMDCSSTHNFVCK(F)	4.95	0.00	0.95	716.2947	7.02		
												4	10.26	10.26	82.60	2.99E+05	(K)GSHLLSLHNIAEADFVLK(K)	8.07	21.74	0.99	491.7730	5.99		
												3	13.63	13.63	88.60	2.90E+05	(K)GSHLLSLHNIAEADFVLK(K)	8.07	21.74	0.98	655.3606	5.99		
												2	23.25	23.25	92.30	4.27E+05	(K)QDCLSDWSFYEGYCYK(V)	8.20	5.53	1.00	1060.9237	4.03		
												3	15.45	15.45	78.70	1.14E+05	(K)QDCLSDWSFYEGYCYK(V)	8.20	5.53	1.00	707.6167	4.03		
												2	23.02	23.02	91.30	7.62E+04	(K)DGVIWMGLNDVWNECNWGWTDGAK(L)	10.40	16.21	0.95	1412.1165	3.84		
4	4.1	9	8	134.32	13.8	7.66E+04	98156.9	7.36	*Daboia russelii*	CL3655.contig2_DrSL	phosphodiesterase 1	2	15.21	15.21	87.80	2.08E+05	(K)AATYFWPGSEVK(I)	7.28	0.00	0.99	678.3375	6.04	0.23%	0.08%
												2	12.64	5.88	81.60	3.44E+04	(R)TLGMLMEGLK(Q)	7.92	0.00	0.99	546.7929	5.66		
												3	16.07	10.05	83.90	4.92E+04	(R)VRDVELLTGLNFYSGLK(Q)	8.73	0.00	0.99	642.0225	6.04		
												2	15.85	10.58	96.50	2.22E+05	(K)TFLPIFVNPVN(-)	9.82	0.00	1.00	630.8549	5.18		
												2	18.95	18.95	82.50	2.99E+04	(K)FGPVSGEIIMALQMADR(T)	9.87	5.40	0.99	917.9637	4.37		
												3	21.16	21.16	90.90	2.62E+04	(K)DQCASSSAAQCPAGFEQSPLILFSMDGFR(A)	10.30	5.40	0.98	1059.8096	4.03		
												3	16.67	16.67	84.70	3.51E+04	(K)GKNEVTSFENIEVYNLMCDLLK(L)	10.45	0.00	0.58	872.7626	4.41		
												3	17.77	8.96	93.00	6.69E+04	(K)NEVTSFENIEVYNLMCDLLK(L)	11.10	7.25	1.00	811.0564	4.00		
												2	15.89	15.89	73.80	1.78E+04	(K)NEVTSFENIEVYNLMCDLLK(L)	11.10	0.00	0.99	1216.0801	4.00		
5	5.1	9	7	122.17	33.0	7.94E+05	29507.5	9.78	*Daboia siamensis*	P18965	Factor V activator RVV-V gamma	2	15.06	15.06	73.10	4.25E+05	(K)YFCLNTK(F)	5.63	5.40	1.00	473.2291	8.59	2.36%	0.79%
												2	16.29	16.29	89.10	8.94E+05	(R)TLCAGILK(G)	5.80	5.40	1.00	438.2551	8.41		
												2	12.88	12.88	82.60	6.86E+05	(R)EWVLTAAHCDR(R)	5.85	0.00	0.99	679.3218	5.32		
												3	18.11	18.11	94.10	8.10E+05	(R)RPVTYSTHIAPVSLPSR(S)	5.85	0.00	0.97	627.6851	10.84		
												3	16.18	16.18	86.50	3.83E+05	(K)FPNGLDKDIMLIR(L)	7.97	5.40	0.99	511.2831	5.96		
												2	18.85	18.85	91.00	3.24E+06	(K)WCEPLYPWVPADSR(T)	8.47	0.00	0.93	888.4186	4.37		
												3	20.49	15.66	95.60	6.35E+05	(K)WCEPLYPWVPADSR(T)	8.47	0.00	0.99	592.6129	4.37		
												2	23.16	23.16	96.10	4.16E+04	(K)VFDYNNWIQSIIAGNR(T)	9.83	5.40	0.96	955.4831	5.80		
												3	19.34	19.34	92.00	2.85E+04	(K)VFDYNNWIQSIIAGNR(T)	9.83	5.40	0.96	637.3242	5.80		
5	5.2	6	4	76.29	21.5	1.15E+06	27523.2	9.03	*Naja naja*	CL31.contig2_Nn	RVV-V gamma-like protein	2	16.29	16.29	89.10	8.94E+05	(R)TLCAGILK(G)	5.80	5.40	1.00	438.2551	8.41	3.41%	1.14%
												3	18.11	18.11	94.10	8.10E+05	(R)RPVTYSTHIAPVSLPSR(S)	5.85	0.00	0.97	627.6851	10.84		

												2	18.85	18.85	91.00	3.24E+06	(K)WCEPLYPWVPADSR(T)	8.47	0.00	0.93	888.4186	4.37		
												3	19.71	15.27	83.20	8.81E+05	(K)SFTPWDKDIMLIR(L)	8.48	5.40	1.00	541.2864	5.68		
												3	20.49	15.66	95.60	6.35E+05	(K)WCEPLYPWVPADSR(T)	8.47	0.00	0.99	592.6129	4.37		
												2	21.40	16.61	84.70	4.29E+05	(K)SFTPWDKDIMLIR(L)	8.48	5.40	1.00	811.4261	5.68		
5	5.3	4	4	66.98	22.6	7.14E+05	28908.7	9.10	*Daboia russelii*	CL2958.contig11_DrSL	serine beta-fibrinogenase-like protein	2	15.06	15.06	73.10	4.25E+05	(K)YFCLNTK(F)	5.63	5.40	1.00	473.2291	8.59	2.12%	0.71%
												2	12.88	12.88	82.60	6.86E+05	(R)EWVLTAAHCDR(R)	5.85	0.00	0.99	679.3218	5.32		
												3	16.18	16.18	86.50	3.83E+05	(K)FPNGLDKDIMLIR(L)	7.97	5.40	0.99	511.2831	5.96		
												3	22.86	13.13	97.90	1.36E+06	(R)LNSPVTYNTHIAPFSLPSSPPTVGSVCR(I)	8.02	0.00	1.00	1000.1784	8.75		
6	6.1	9	6	113.88	11.9	2.17E+05	71801.6	6.01	*Daboia russelii*	K9JAW0	factor X activator heavy chain	4	16.24	16.24	84.70	3.82E+04	(R)ARNECDVPEHCTGQSAECPR(D)	4.15	0.00	0.99	594.0000	4.83	0.64%	0.21%
												3	23.95	23.95	100.00	2.75E+04	(R)ARNECDVPEHCTGQSAECPR(D)	4.23	0.00	-0.26	791.6661	4.83		
												3	19.69	19.69	82.30	7.83E+04	(R)NECDVPEHCTGQSAECPR(D)	4.30	5.40	0.99	715.9525	4.40		
												3	14.45	14.45	78.80	6.31E+05	(R)KIPCAPQDVK(C)	4.58	0.00	0.99	385.8792	8.59		
												2	18.48	18.48	93.80	3.04E+05	(R)KIPCAPQDVK(C)	4.58	0.00	0.99	578.3145	8.59		
												3	17.33	17.33	79.10	3.04E+05	(K)LKPGAECGNGLCCYQCK(I)	4.95	0.00	0.94	672.2922	8.50		
												2	14.15	7.34	84.70	3.26E+05	(K)CILYPPLR(K)	6.78	5.40	1.00	516.2893	8.75		
												3	20.28	20.28	82.60	1.55E+05	(R)FDLNTLGITFLAGMCQAYR(S)	10.90	10.30	1.00	731.0289	5.83		
												2	19.88	19.88	94.70	8.74E+04	(R)FDLNTLGITFLAGMCQAYR(S)	10.90	10.30	0.98	1096.0382	5.83		
6	6.2	8	6	111.95	10.6	3.08E+05	71793.7	6.05	*Daboia russelii*	Unigene32626_DrSL	factor X activator heavy chain	4	16.24	16.24	84.70	3.82E+04	(R)ARNECDVPEHCTGQSAECPR(D)	4.15	0.00	0.99	594.0000	4.83	0.91%	0.30%
												3	23.95	23.95	100.00	2.75E+04	(R)ARNECDVPEHCTGQSAECPR(D)	4.23	0.00	-0.26	791.6661	4.83		
												3	19.69	19.69	82.30	7.83E+04	(R)NECDVPEHCTGQSAECPR(D)	4.30	5.40	0.99	715.9525	4.40		
												3	14.45	14.45	78.80	6.31E+05	(R)KIPCAPQDVK(C)	4.58	0.00	0.99	385.8792	8.59		
												2	18.48	18.48	93.80	3.04E+05	(R)KIPCAPQDVK(C)	4.58	0.00	0.99	578.3145	8.59		
												3	17.33	17.33	79.10	3.04E+05	(K)LKPGAECGNGLCCYQCK(I)	4.95	0.00	0.94	672.2922	8.50		
												2	18.35	18.35	88.40	7.53E+05	(R)LFCLNNSPGNK(N)	5.93	0.00	1.00	632.3138	8.75		
												2	14.15	7.34	84.70	3.26E+05	(K)CILYPPLR(K)	6.78	5.40	1.00	516.2893	8.75		
6	6.3	6	4	69.00	51.8	3.38E+05	12226.6	9.44	*Naja naja*	Unigene31385_Nn	Zinc metalloproteinase-disintegrin VLAIP-A	3	14.45	14.45	78.80	6.31E+05	(R)KIPCAPQDVK(C)	4.58	0.00	0.99	385.8792	8.59	1.00%	0.33%
												2	18.48	18.48	93.80	3.04E+05	(R)KIPCAPQDVK(C)	4.58	0.00	0.99	578.3145	8.59		
												2	16.78	16.78	86.70	2.24E+05	(K)NPCQIYYAPSDENK(G)	5.30	0.00	1.00	849.8777	4.37		
												2	12.94	12.94	72.40	6.52E+05	(K)QCISLFGSR(A)	6.67	0.00	1.00	534.2704	9.75		
												2	20.80	20.80	76.00	1.12E+05	(R)ATVAQDACFNFNSLGNAYGYCR(K)	7.93	5.40	1.00	1250.0460	5.88		
												3	17.45	17.45	85.80	1.05E+05	(R)ATVAQDACFNFNSLGNAYGYCR(K)	7.95	10.81	0.99	833.7002	5.88		
6	6.4	5	3	53.98	6.8	2.09E+05	70876.8	5.45	*Macrovipera lebetina*	Q4VM08	Zinc metalloproteinase-disintegrin-like VLAIP-A	3	14.45	14.45	78.80	6.31E+05	(R)KIPCAPQDVK(C)	4.58	0.00	0.99	385.8792	8.59	0.62%	0.21%
												2	18.48	18.48	93.80	3.04E+05	(R)KIPCAPQDVK(C)	4.58	0.00	0.99	578.3145	8.59		
												2	22.47	12.27	96.30	9.05E+04	(R)IYEIVNILNVIYR(V)	10.63	0.00	1.00	811.4710	6.00		
												3	20.33	6.31	100.00	1.63E+04	(R)IYEIVNILNVIYR(V)	10.63	0.00	0.98	541.3158	6.00		
												3	13.03	13.03	77.30	5.24E+03	(R)VLNIYIALLGLEIWNNGDK(I)	11.67	0.00	0.64	720.0665	4.37		
7	7.1	10	5	83.13	55.7	2.40E+06	16251.0	4.65	*Daboia siamensis*	Q7T2R1	Acidic phospholipase A2 daboiatoxin A chain	2	21.60	21.60	90.40	1.86E+06	(R)CCFVHDCCYGTVNDCNPK(M)	5.22	0.00	0.93	1153.4358	5.21	7.13%	2.38%
												2	14.73	14.73	84.30	3.60E+06	(K)NYENYAISHCTEESEQC(-)	5.38	10.81	0.98	1067.4152	4.09		
												3	12.68	12.68	74.10	1.17E+06	(K)NYENYAISHCTEESEQC(-)	5.38	10.81	1.00	711.9453	4.09		
												2	20.60	20.60	89.10	8.42E+06	(R)AAAICLGQNVNTYDK(N)	6.20	97.40	0.92	819.3868	5.88		
												3	17.35	12.39	86.50	1.49E+05	(R)AAAICLGQNVNTYDK(N)	6.33	21.74	0.99	546.5942	5.88		
												4	15.29	15.29	83.50	5.53E+06	(K)EAVHSYAIYGCYCGWGGQGKPQDATDR(C)	6.42	27.15	0.98	762.3393	5.38		
												3	26.20	26.20	100.00	2.17E+06	(K)EAVHSYAIYGCYCGWGGQGKPQDATDR(C)	6.42	21.75	0.99	1016.1157	5.38		
												2	20.54	15.34	91.90	7.61E+05	(R)AAAICLGQNVNTYDK(N)	8.13	156.99	0.99	819.4014	5.88		
												3	12.59	12.59	84.50	3.28E+05	(-)NFFQFAEMIVK(M)	10.15	10.81	1.00	458.5704	6.00		
												4	13.37	13.37	73.60	4.47E+03	(K)EAVHSYAIYGCYCGWGGQGKPQDATDR(C)	14.85	0.00	-0.43	762.3374	5.38		
7	7.2	8	3	62.65	43.4	1.38E+06	16441.3	4.93	*Daboia siamensis*	A8CG78	Acidic phospholipase A2 DsM-a2	2	17.25	17.25	81.70	2.43E+06	(K)YMLYSIFDCKEESDQC(-)	8.37	10.82	0.94	1044.4258	3.92	4.10%	1.37%
												3	10.96	10.96	75.30	9.18E+05	(K)YMLYSIFDCKEESDQC(-)	8.37	10.82	0.97	696.6189	3.92		
												3	22.81	22.81	87.20	2.62E+06	(K)TGNFGLLSYVYYGCYCGWGGK(G)	9.70	21.62	0.99	808.0287	8.05		
												2	20.01	20.01	90.40	2.60E+06	(K)TGNFGLLSYVYYGCYCGWGGK(G)	9.68	21.62	0.99	1211.5381	8.05		
												3	22.26	22.26	95.00	1.86E+06	(K)TATYSYSFENGDIVCGGDDPCLR(A)	11.85	178.85	0.95	866.3703	3.84		
												3	19.30	14.72	80.80	5.65E+05	(K)TATYSYSFENGDIVCGGDDPCLR(A)	12.52	35.81	0.90	866.3703	3.84		
												3	16.74	16.74	72.80	4.18E+04	(K)TGNFGLLSYVYYGCYCGWGGK(G)	14.38	1.87	0.97	808.0269	8.05		
												3	22.59	22.59	90.00	1.05E+04	(K)TATYSYSFENGDIVCGGDDPCLR(A)	14.88	0.00	0.79	866.3703	3.84		
8	8.1	4	4	83.65	45.2	7.24E+05	17267.5	5.63	*Daboia siamensis*	Q4PRC9	Snaclec 4	2	20.41	20.41	88.30	3.16E+05	(K)SMTCNFIAPVVCK(F)	6.95	5.40	1.00	763.8642	8.47	2.15%	0.72%
												2	24.56	24.56	95.90	1.75E+06	(K)VFTEEMNWADAEK(F)	7.02	0.00	1.00	785.3515	4.00		
												3	20.73	11.23	88.00	7.32E+05	(K)ALAEESYCLIMITHEK(V)	7.73	43.23	0.96	636.6480	4.75		
												2	17.95	17.95	89.60	9.88E+04	(K)FVVNLISENLEYPATWIGLGNMWK(D)	11.45	6.63	0.97	1397.7161	4.53		
8	8.2	6	4	77.36	48.6	7.82E+05	17366.4	6.03	*Daboia siamensis*	Q4PRD0	Snaclec 3	4	11.57	11.57	72.00	1.53E+06	(K)NHWSHMDCSSTHNFVCK(F)	4.95	0.00	0.97	537.4732	7.02	2.32%	0.77%
												3	16.15	16.15	83.30	7.47E+05	(K)NHWSHMDCSSTHNFVCK(F)	4.95	0.00	0.95	716.2947	7.02		
												2	24.56	24.56	95.90	1.75E+06	(K)VFTEEMNWADAEK(F)	7.02	0.00	1.00	785.3515	4.00		
												4	10.26	10.26	82.60	2.99E+05	(K)GSHLLSLHNIAEADFVLK(K)	8.07	21.74	0.99	491.7730	5.99		
												3	13.63	13.63	88.60	2.90E+05	(K)GSHLLSLHNIAEADFVLK(K)	8.07	21.74	0.98	655.3606	5.99		
												2	23.02	23.02	91.30	7.62E+04	(K)DGVIWMGLNDVWNECNWGWTDGAK(L)	10.40	16.21	0.95	1412.1165	3.84		
9	9.1	5	4	78.86	39.1	2.40E+06	16698.9	9.60	*Daboia siamensis*	A8CG82	Basic phospholipase A2 DsM-b1	2	20.69	20.69	78.40	3.13E+06	(R)CCFVHDCCYAR(V)	4.87	10.81	0.97	774.2887	6.74	7.15%	2.38%
												3	14.36	5.06	82.10	7.97E+05	(R)VAAICLGQNVNTYNK(G)	6.12	0.00	1.00	555.6092	8.56		
												2	25.83	25.83	97.60	3.31E+06	(K)YISYGCYCGWGGQGTPK(D)	6.57	0.00	0.89	977.4201	8.43		
												3	18.49	18.49	88.20	7.03E+05	(K)YISYGCYCGWGGQGTPK(D)	6.57	0.00	1.00	651.9490	8.43		
												2	17.98	14.24	80.40	4.08E+06	(K)GYMFLSSYYCR(Q)	7.87	5.40	0.95	723.8157	8.50		
9	9.2	5	3	63.77	33.8	2.10E+06	15683.6	5.81	*Daboia siamensis*	Q7T3T5	Acidic phospholipase A2 daboiatoxin B chain	2	20.69	20.69	78.40	3.13E+06	(R)CCFVHDCCYAR(V)	4.87	10.81	0.97	774.2887	6.74	6.24%	2.08%
												2	25.83	25.83	97.60	3.31E+06	(K)YISYGCYCGWGGQGTPK(D)	6.57	0.00	0.89	977.4201	8.43		
												3	18.49	18.49	88.20	7.03E+05	(K)YISYGCYCGWGGQGTPK(D)	6.57	0.00	1.00	651.9490	8.43		
												2	17.25	17.25	81.70	2.43E+06	(K)YMLYSIFDCKEESDQC(-)	8.37	10.82	0.94	1044.4258	3.92		
												3	10.96	10.96	75.30	9.18E+05	(K)YMLYSIFDCKEESDQC(-)	8.37	10.82	0.97	696.6189	3.92		
10	10.1	7	5	76.94	24.3	1.03E+06	29126.9	7.94	*Daboia russelii*	CL2958.contig6_DrSL	Serine protease VLSP-1	2	16.29	16.29	89.10	8.94E+05	(R)TLCAGILK(G)	5.80	5.40	1.00	438.2551	8.41	3.06%	1.02%
												2	14.67	14.67	72.90	6.00E+05	(R)FFCLSNK(N)	6.12	0.00	1.00	458.2149	8.75		
												2	16.39	16.39	83.20	1.61E+06	(R)HPCAQAELPAFYTK(V)	6.20	0.00	0.99	816.8822	6.75		
												3	15.67	15.67	84.10	1.43E+06	(R)HPCAQAELPAFYTK(V)	6.20	0.00	1.00	544.9239	6.75		
												3	22.86	13.13	97.90	1.36E+06	(R)LNSPVTYNTHIAPFSLPSSPPTVGSVCR(I)	8.02	0.00	1.00	1000.1784	8.75		
												3	19.71	15.27	83.20	8.81E+05	(K)SFTPWDKDIMLIR(L)	8.48	5.40	1.00	541.2864	5.68		
												2	21.40	16.61	84.70	4.29E+05	(K)SFTPWDKDIMLIR(L)	8.48	5.40	1.00	811.4261	5.68		
11	11.1	5	3	59.00	10.0	3.06E+05	55887.4	5.44	*Daboia russelii*	CL3662.contig2_DrSL	Zinc metalloproteinase-disintegrin VLAIP-A	3	13.18	13.18	75.90	9.41E+05	(K)LTQGSQCADEECCDQCK(F)	4.03	3.76	0.94	696.9310	3.91	0.91%	0.30%
												2	23.50	23.50	92.70	4.78E+05	(K)LTQGSQCADEECCDQCK(F)	4.05	3.76	0.96	1044.8909	3.91		
												2	22.47	12.27	96.30	9.05E+04	(R)IYEIVNILNVIYR(V)	10.63	0.00	1.00	811.4710	6.00		
												3	20.33	6.31	100.00	1.63E+04	(R)IYEIVNILNVIYR(V)	10.63	0.00	0.98	541.3158	6.00		
												3	13.03	13.03	77.30	5.24E+03	(R)VLNIYIALLGLEIWNNGDK(I)	11.67	0.00	0.64	720.0665	4.37		
12	12.1	4	3	53.06	24.6	5.06E+05	17905.9	7.07	*Daboia siamensis*	Q38L02	Snaclec dabocetin subunit alpha	3	23.19	23.19	95.60	8.27E+05	(R)QQCSSHWTDGSAVSYETVTK(Y)	5.67	0.00	0.99	757.6730	5.32	1.50%	0.50%
												2	23.01	23.01	93.40	4.05E+05	(R)QQCSSHWTDGSAVSYETVTK(Y)	5.67	0.00	1.00	1136.0050	5.32		
												3	15.64	9.34	91.50	5.03E+05	(K)YHEWITLPCGDK(N)	6.75	0.00	1.00	506.9073	5.32		
												4	14.23	6.47	79.80	2.87E+05	(K)YHEWITLPCGDKNPFICK(S)	7.77	5.40	0.98	570.2777	6.75		

13	13.1	2	2	47.50	38.0	2.19E+06	9712.8	6.82	*Daboia siamensis*	A8Y7P4	Kunitz-type serine protease inhibitor B4	2	19.12	19.12	88.70	3.07E+06	(R)IYYNLESNK(C)	5.40	5.40	0.86	572.2907	6.00	6.49%	2.16%
												2	28.38	28.38	97.90	1.30E+06	(K)CEVFFYGGCGGNDNNFSTWDECR(H)	8.12	10.94	0.85	1396.0368	3.91		
13	13.2	2	2	34.45	38.3	3.58E+06	7191.8	9.69	*Daboia siamensis*	P00990	Kunitz-type serine protease inhibitor 2	4	15.33	15.33	86.40	4.08E+06	(-)HDRPTFCNLAPESGR(C)	5.15	5.40	0.99	439.9559	6.75	10.63%	3.54%
												2	19.12	19.12	88.70	3.07E+06	(R)IYYNLESNK(C)	5.40	5.40	0.86	572.2907	6.00		
14	14.1	3	3	46.12	16.2	1.75E+05	29180.4	9.89	*Daboia siamensis*	E5L0E3	Alpha-fibrinogenase-like	2	16.97	10.99	81.60	1.52E+04	(R)TLCAGVSGR(R)	4.32	0.00	0.97	460.7351	9.41	0.52%	0.17%
												4	14.10	5.45	81.00	4.14E+05	(K)LNKPVTYSTHIASLSLPSNPPR(V)	6.48	0.13	1.00	598.8328	9.99		
												2	15.05	15.05	71.90	9.45E+04	(R)IMGWGSITSPK(K)	7.02	0.00	1.00	588.8087	8.75		
15	15.1	3	3	25.80	11.3	3.07E+05	18793.1	5.84	*Daboia siamensis*	Q4PRD2	Snaclec coagulation factor X-activating enzyme light chain 2	2	19.53	14.99	92.60	1.78E+05	(-)LDCPPDSSLYR(Y)	5.53	10.81	0.99	661.8077	4.21	0.91%	0.30%
												2	13.75	13.75	74.40	6.78E+05	(R)YLFVCK(V)	6.03	0.00	1.00	415.2099	8.59		
												3	12.05	12.05	73.10	6.39E+04	(K)FITHFWIGLMIK(D)	9.63	27.03	0.98	502.6171	8.76		
16	16.1	2	2	43.61	21.3	9.18E+05	13625.1	9.69	*Daboia russelii*	P30894	Venom nerve growth factor	2	23.11	23.11	96.60	7.56E+05	(R)INTACVCVISR(K)	5.67	0.00	0.99	646.8270	9.75	2.73%	0.91%
												2	20.50	14.17	85.80	1.08E+06	(K)HWNSYCTTTDTFVR(A)	5.93	0.00	1.00	894.3985	6.74		
17	17.1	3	2	37.43	17.0	1.41E+06	18452.4	5.38	*Daboia siamensis*	K9JBV0	P68 alpha subunit	2	18.75	18.75	89.90	1.72E+06	(K)TPADYVWIGLR(N)	8.30	5.41	1.00	645.8475	5.50	4.19%	1.40%
												3	13.76	13.76	77.60	2.98E+04	(K)TPADYVWIGLR(N)	8.30	5.41	1.00	430.8995	5.50		
												2	18.68	18.68	91.30	2.48E+06	(R)TWFNLSCGDDYPFVCK(F)	8.87	5.40	0.99	1004.9344	4.21		
18	18.1	3	2	37.17	33.0	1.57E+06	13010.7	7.20	*Daboia siamensis*	P0DL42	Snake venom vascular endothelial growth factor toxin VR-1	2	12.81	12.81	72.10	1.63E+06	(K)HTADIQIMR(M)	5.03	0.00	0.98	542.7840	6.74	4.68%	1.56%
												3	24.36	19.44	98.50	2.83E+06	(R)ETLVSILQEHPDEISDIFRPSCVAVLR(C)	9.42	5.40	1.00	1041.8800	4.50		
												5	15.14	5.60	80.90	2.60E+05	(R)ETLVSILQEHPDEISDIFRPSCVAVLR(C)	9.42	5.40	1.00	625.5306	4.50		
19	19.1	3	2	36.15	5.5	3.93E+04	77077.2	5.92	*Boiga irregularis*	A0A0B8RNS9	Xaa-Pro aminopeptidase 2	2	15.51	15.51	82.10	2.01E+04	(R)GDDIPYTPVFYAYTLLTK(T)	10.83	5.40	0.95	1039.0300	4.21	0.12%	0.04%
												3	12.09	5.63	72.30	1.29E+04	(R)GDDIPYTPVFYAYTLLTK(T)	10.80	0.00	0.88	693.0250	4.21		
												3	20.64	16.56	92.10	8.50E+04	(K)KPTAILLSGLEETAWLFNLR(G)	11.08	7.25	0.95	758.0958	6.14		
19	19.2	3	2	32.86	5.4	2.74E+04	74587.3	5.63	*Daboia russelii*	Unigene32033_DrSL	xaa-Pro aminopeptidase 2-like	2	15.51	15.51	82.10	2.01E+04	(R)GDDIPYTPVFYAYTLLTK(T)	10.83	5.40	0.95	1039.0300	4.21	0.08%	0.03%
												3	12.09	5.63	72.30	1.29E+04	(R)GDDIPYTPVFYAYTLLTK(T)	10.80	0.00	0.88	693.0250	4.21		
												3	17.35	8.71	82.70	4.93E+04	(R)TLDLLEVNLVDLVWGSER(L)	11.50	10.07	0.89	691.0408	3.91		
20	20.1	2	2	35.11	6.9	3.23E+04	65308.5	9.01	*Crotalus adamanteus*	F8S0Z7	Snake venom 5'-nucleotidase	3	17.58	9.96	87.90	3.61E+04	(K)YLGYLNVIFDDKGNVIK(A)	9.02	5.41	0.92	657.6923	5.96	0.10%	0.03%
												3	17.53	13.07	87.10	2.84E+04	(R)YDAMALGNHEFDNGLAGLLDPLLK(H)	10.35	0.00	0.94	863.1009	4.22		
20	20.2	2	2	32.02	4.7	7.36E+04	63610.7	9.15	*Daboia russelii*	CL3322.contig1_DrSL	Snake venom 5'-nucleotidase	2	14.44	14.44	81.00	1.11E+05	(R)VVSLNVLCTK(C)	6.75	0.00	0.99	566.8242	8.72	0.22%	0.07%
												3	17.58	9.96	87.90	3.61E+04	(K)YLGYLNVIFDDKGNVIK(A)	9.02	5.41	0.92	657.6923	5.96		
21	21.1	3	2	34.76	8.9	5.39E+05	28719.5	7.07	*Daboia siamensis*	E5L0E4	Beta-fibrinogenase-like	2	14.67	14.67	72.90	6.00E+05	(R)FFCLSNK(N)	6.12	0.00	1.00	458.2149	8.75	1.60%	0.53%
												2	17.83	12.44	96.90	8.77E+05	(K)TSTYIAPLSLPSSPPR(V)	7.37	5.40	0.98	843.9601	8.41		
												3	20.09	12.31	97.80	1.40E+05	(K)TSTYIAPLSLPSSPPR(V)	7.35	5.40	0.98	562.9753	8.41		
22	22.1	3	2	34.74	27.7	1.44E+06	10243.6	9.57	*Daboia siamensis*	A8Y7P5	Kunitz-type serine protease inhibitor B5	2	15.20	6.67	80.00	1.76E+06	(R)SFYYDSESK(K)	5.03	0.00	0.98	563.2429	4.37	4.27%	1.42%
												3	18.02	18.02	77.80	2.18E+06	(K)FCYLPADPGECLAHMR(S)	7.45	10.81	0.97	646.2907	5.32		
												2	19.54	19.54	85.60	3.67E+05	(K)FCYLPADPGECLAHMR(S)	7.45	10.81	0.98	968.9320	5.32		
23	23.1	3	2	31.42	14.1	1.84E+05	18116.4	9.16	*Macrovipera lebetina*	B4XSY7	Snaclec A12	3	15.19	15.19	81.10	7.44E+04	(K)LETHVWIGLR(V)	6.77	5.40	0.97	408.5680	6.75	0.55%	0.18%
												2	16.23	11.29	79.60	4.44E+05	(K)ANFVAELVTLMK(L)	10.73	5.40	1.00	668.3720	6.05		
												3	13.55	7.85	83.50	3.44E+04	(K)ANFVAELVTLMK(L)	10.73	5.40	0.98	445.9168	6.05		
24	24.1	2	1	26.84	6.9	1.05E+05	29577.7	9.77	*Macrovipera lebetina*	Q9PT40	Venom serine proteinase-like protein 2	2	26.84	26.84	91.70	1.16E+05	(R)FYCAGTLINQEWVLTAAR(C)	8.98	5.40	0.99	1057.0314	6.00	0.31%	0.10%
												3	18.44	18.44	80.20	9.32E+04	(R)FYCAGTLINQEWVLTAAR(C)	9.03	16.34	0.90	705.0248	6.00		
25	25.1	1	1	23.47	11.3	1.31E+05	18504.6	6.41	*Daboia siamensis*	K9JBU9	P31 alpha subunit	2	23.47	23.47	100.00	1.31E+05	(K)SVGEANFVAQLASGFMQK(D)	9.78	5.40	1.00	942.4717	5.72	0.39%	0.13%
26	26.1	1	1	21.59	5.8	4.79E+05	28981.6	7.11	*Macrovipera lebetina*	E0Y419	Beta-fibrinogenase	2	21.59	21.59	94.20	4.79E+05	(R)TLCAGILQGGIDTCK(G)	6.83	5.40	0.99	803.9011	5.50	1.42%	0.47%
27	27.1	2	1	20.63	80.0	2.59E+05	1698.8	4.65	*Daboia russelii*	P86531	Vipera russelli proteinase RVV-V homolog 2	3	20.63	20.63	93.60	4.68E+05	(-)VVGGDECNINEHR(F)	4.23	5.40	0.96	500.2297	4.65	0.77%	0.26%
												2	19.94	13.23	83.50	4.93E+04	(-)VVGGDECNINEHR(F)	4.23	0.00	0.99	749.8402	4.65		
28	28.1	2	1	20.06	6.6	2.48E+05	27826.7	5.61	*Gloydius blomhoffii*	Q8JI40	Cysteine-rich venom protein ablomin	3	20.06	20.06	96.10	2.48E+05	(R)KPEIQNEIVDLHNSLR(R)	6.57	0.00	0.96	635.6798	5.45	0.74%	0.25%
												4	12.62	3.53	81.20	2.47E+05	(R)KPEIQNEIVDLHNSLR(R)	6.57	0.00	0.95	477.0115	5.45		
						3.36E+07																	100.00%	

Daboia siamensis Guangxi venom 2

Group	Subgroup	Spectra	Distinct Peptides	Distinct Summed MS/MS Search Score	% AA Coverage	Mean Protein Spectral Intensity	Protein MW (Da)	Protein pI	Species	Database Accession	Protein Name	z	Score	Fwd-Rev Score	SPI (%)	Spectrum Intensity	Sequence	RT (min)	Peak Width (sec)	Averagine Chi Squared	m/z Measured (Da)	Peptide pI	Relative abundance (n=1)	Relative abundance (% overall)
1	1.1	17	10	185.33	22.6	1.70E+06	71721.7	5.95	*Daboia russelii*	B8K1W0	Zinc metalloproteinase-disintegrin-like daborhagin-K	3	27.55	27.55	97.7	1.91E+05	(R)GEECDCGSPENCRDPCCDAASCK(L)	4.18	0.00	0.87	911.9878	4.02	6.12%	2.04%
												2	26.99	26.99	91.9	4.22E+05	(R)GEECDCGSPENCRDPCCDAASCK(L)	4.28	0.00	0.88	1367.4738	4.02		
												4	12.36	12.36	75.0	8.39E+04	(R)GEECDCGSPENCRDPCCDAASCK(L)	4.28	0.00	0.71	684.2413	4.02		
												2	20.12	20.12	77.6	3.30E+06	(K)LHSWVECESGK(C)	4.85	5.40	0.97	666.3087	5.40		
												3	16.66	16.66	84.7	3.46E+06	(K)LHSWVECESGK(C)	4.85	5.40	0.95	444.5419	5.40		
												2	16.43	8.78	94.0	7.26E+05	(K)YKNDLTAIR(T)	4.87	5.40	1.00	547.3043	8.59		
												2	21.73	11.28	95.5	4.99E+06	(R)TDIVSPPACGNELLER(G)	6.72	10.81	0.92	885.9398	4.14		
												4	14.23	14.23	83.6	2.54E+05	(R)RFLTEHNPECIINPPLR(T)	6.55	5.40	1.00	527.2798	6.76		
												3	21.55	21.55	93.3	6.74E+05	(R)TDIVSPPACGNELLER(G)	6.72	0.00	0.99	590.9621	4.14		
												3	21.83	21.83	97.9	6.16E+06	(R)FLTEHNPECIINPPLR(T)	6.95	5.40	0.92	650.6717	5.40		
												2	23.19	23.19	98.5	2.32E+06	(R)FLTEHNPECIINPPLR(T)	6.95	5.40	1.00	975.5030	5.40		
												2	16.64	16.64	93.9	1.64E+05	(K)VCSNGQCVDLNIAY(-)	7.23	5.40	0.99	806.8611	3.80		
												2	14.93	14.93	88.6	2.87E+06	(K)SPGNQIPCLPYYIPSDENK(G)	7.72	5.40	0.99	1096.5246	4.37		
												3	13.34	8.95	93.0	1.13E+06	(K)SPGNQIPCLPYYIPSDENK(G)	7.72	5.40	1.00	731.3523	4.37		
												3	13.72	7.53	83.9	8.57E+04	(R)VPLVGLEIWK(N)	8.95	10.81	0.99	385.2378	5.97		
												3	16.79	8.15	83.0	2.00E+06	(R)TWVFELVNTINEIFKYLYIR(V)	14.42	18.39	0.98	854.4667	5.81		
												4	12.68	8.88	82.4	1.77E+04	(R)TWVFELVNTINEIFKYLYIR(V)	14.47	0.13	0.76	641.1028	5.81		
2	2.1	15	11	184.18	26.3	3.52E+05	57286.2	8.82	*Daboia russelii*	G8XQX1	L-amino-acid oxidase	2	19.34	11.51	87.0	6.10E+05	(K)VTVTYQTTQK(N)	4.55	0.00	0.99	584.8158	8.56	1.27%	0.42%
												3	15.20	7.38	74.8	6.56E+05	(K)VTVLEASERPGGR(V)	4.88	5.40	0.99	457.5851	6.11		
												2	18.28	7.95	94.4	1.48E+05	(K)VTVLEASERPGGR(V)	4.87	5.40	0.95	685.8741	6.11		

												2	19.96	12.01	93.1	9.14E+05	(K)SAGQLYQESLGK(A)	5.45	0.00	0.99	640.8292	5.72		
												2	14.06	14.06	74.0	1.63E+05	(K)IFLTCTK(K)	5.48	5.40	1.00	441.7422	8.75		
												2	14.42	14.42	100.0	4.39E+05	(K)HDDIFAYEK(R)	5.55	0.00	1.00	569.2663	4.54		
												3	13.40	5.40	81.8	1.58E+05	(R)ITFKPPLPPK(K)	6.33	21.62	1.00	379.9067	10.00		
												2	16.89	8.81	90.9	1.18E+05	(K)KDLQTFCYPSIIQK(W)	6.82	5.40	0.99	870.9547	8.50		
												2	17.20	17.20	83.5	1.76E+05	(K)EGWYANLGPMR(V)	7.25	0.00	1.00	647.3081	6.10		
												2	21.92	21.92	94.0	2.14E+05	(K)DLQTFCYPSIIQK(W)	7.75	5.40	0.99	806.9078	5.83		
												3	13.43	7.80	74.0	2.08E+04	(K)DLQTFCYPSIIQK(W)	7.75	5.40	0.79	538.2742	5.83		
												3	15.79	4.99	79.3	3.50E+05	(R)IFFAGEYTANAHGWIDSTIK(S)	9.42	0.00	0.98	747.7062	5.32		
												3	12.20	12.20	89.7	1.40E+05	(K)YAMGAITTFTPYQFQHFSEALTAPVGR(I)	9.42	0.00	1.00	1002.1645	6.75		
												3	16.51	6.86	85.6	1.17E+06	(R)IFFAGEYTANAHGWIDSTIK(S)	11.80	180.70	0.98	747.7048	5.32		
												3	14.04	5.09	86.2	8.02E+03	(R)IFFAGEYTANAHGWIDSTIK(S)	13.08	0.00	0.91	747.7048	5.32		
2	2.2	15	10	168.82	30.9	7.29E+05	46713.1	7.90	*Daboia siamensis*	Q4F867	L-amino-acid oxidase	2	19.34	11.51	87.0	6.10E+05	(K)VTVTYQTTQK(N)	4.55	0.00	0.99	584.8158	8.56	2.63%	0.88%
												2	19.96	12.01	93.1	9.14E+05	(K)SAGQLYQESLGK(A)	5.45	0.00	0.99	640.8292	5.72		
												2	14.06	14.06	74.0	1.63E+05	(K)IFLTCTK(K)	5.48	5.40	1.00	441.7422	8.75		
												2	14.42	14.42	100.0	4.39E+05	(K)HDDIFAYEK(R)	5.55	0.00	1.00	569.2663	4.54		
												3	13.40	5.40	81.8	1.58E+05	(R)ITFKPPLPPK(K)	6.33	21.62	1.00	379.9067	10.00		
												2	16.89	8.81	90.9	1.18E+05	(K)KDLQTFCYPSIIQK(W)	6.82	5.40	0.99	870.9547	8.50		
												2	21.92	21.92	94.0	2.14E+05	(K)DLQTFCYPSIIQK(W)	7.75	5.40	0.99	806.9078	5.83		
												3	13.43	7.80	74.0	2.08E+04	(K)DLQTFCYPSIIQK(W)	7.75	5.40	0.79	538.2742	5.83		
												3	15.79	4.99	79.3	3.50E+05	(R)IFFAGEYTANAHGWIDSTIK(S)	9.42	0.00	0.98	747.7062	5.32		
												3	12.20	12.20	89.7	1.40E+05	(K)YAMGAITTFTPYQFQHFSEALTAPVGR(I)	9.42	0.00	1.00	1002.1645	6.75		
												3	16.51	6.86	85.6	1.17E+06	(R)IFFAGEYTANAHGWIDSTIK(S)	11.80	180.70	0.98	747.7048	5.32		
												3	20.12	12.86	95.5	4.01E+06	(K)NLLLETVDYVIVCTTSR(A)	11.30	16.12	1.00	666.0208	4.37		
												2	18.39	13.14	86.7	2.15E+06	(K)NLLLETVDYVIVCTTSR(A)	11.30	16.12	0.99	998.5263	4.37		
												3	13.71	13.71	73.3	4.77E+05	(K)NLLLETVDYVIVCTTSR(A)	12.35	35.25	0.96	666.0195	4.37		
												3	14.04	5.09	86.2	8.02E+03	(R)IFFAGEYTANAHGWIDSTIK(S)	13.08	0.00	0.91	747.7048	5.32		
2	2.3	5	2	32.87	48.8	4.59E+05	10351.4	5.10	*Vipera berus berus*	P0C2D7	L-amino-acid oxidase	3	15.79	4.99	79.3	3.50E+05	(K)IFFAGEYTANAHGWIDSTIK(-)	9.42	0.00	0.98	747.7062	5.32	1.66%	0.55%
												4	11.48	4.10	91.0	4.78E+05	(-)ADDKNPLEECFREDDYEEFLEIAK(N)	8.62	10.81	0.99	744.5913	3.96		
												3	16.36	8.78	85.8	2.90E+05	(-)ADDKNPLEECFREDDYEEFLEIAK(N)	8.63	5.40	0.99	992.4495	3.96		
												3	16.51	6.86	85.6	1.17E+06	(K)IFFAGEYTANAHGWIDSTIK(-)	11.80	180.70	0.98	747.7048	5.32		
												3	14.04	5.09	86.2	8.02E+03	(K)IFFAGEYTANAHGWIDSTIK(-)	13.08	0.00	0.91	747.7048	5.32		
2	2.4	3	2	29.37	4.8	2.89E+05	58619.2	6.05	*Calloselasma rhodostoma*	P81382	L-amino-acid oxidase	2	14.42	14.42	100.0	4.39E+05	(K)HDDIFAYEK(R)	5.55	0.00	1.00	569.2663	4.54	1.04%	0.35%
												2	11.08	5.69	74.0	3.18E+05	(R)LNEFSQENDNAWYFIK(N)	8.77	5.40	0.98	1009.4832	4.14		
												3	14.95	7.00	85.1	1.11E+05	(R)LNEFSQENDNAWYFIK(N)	8.77	5.40	0.99	673.3250	4.14		
3	3.1	11	9	165.52	15.5	4.29E+05	71801.6	6.01	*Daboia russelii*	K9JAW0	factor X activator heavy chain	3	23.12	23.12	98.5	2.95E+04	(R)ARNECDVPEHCTGQSAECPR(D)	4.18	0.00	0.32	791.6647	4.83	1.55%	0.52%
												3	20.17	20.17	85.4	1.29E+05	(R)NECDVPEHCTGQSAECPR(D)	4.28	0.00	0.98	715.9535	4.40		
												3	14.67	14.67	79.2	2.52E+05	(R)KIPCAPQDVK(C)	4.63	5.40	0.99	385.8789	8.59		
												3	18.12	18.12	85.9	6.27E+05	(K)LKPGAECGNGLCCYQCK(I)	4.92	0.00	0.97	672.2926	8.50		
												2	25.76	18.96	94.6	2.15E+05	(K)LKPGAECGNGLCCYQCK(I)	4.92	0.00	0.94	1007.9351	8.50		
												2	17.35	8.47	97.5	1.48E+06	(R)SVGIVQVQGNR(N)	5.10	0.00	1.00	578.8281	9.47		
												3	13.26	10.00	81.1	2.05E+05	(K)CILYPPLRK(D)	5.90	0.00	0.98	387.2280	9.99		
												2	12.66	7.05	78.2	7.94E+04	(K)CILYPPLRK(D)	5.90	0.00	1.00	580.3386	9.99		
												2	16.11	16.11	76.1	8.31E+05	(R)NQCISLFGSR(A)	6.62	0.00	0.99	591.2910	9.75		
												2	14.70	7.88	85.4	7.39E+05	(K)CILYPPLR(K)	6.80	0.00	1.00	516.2899	8.75		
												3	20.38	16.94	85.0	1.33E+05	(R)FDLNTLGITFLAGMCQAYR(S)	10.92	10.81	0.95	731.0288	5.83		
3	3.2	11	9	164.98	14.2	4.50E+05	71793.7	6.05	*Daboia russelii*	Unigene32626_DrSL	factor X activator heavy chain	3	23.12	23.12	98.5	2.95E+04	(R)ARNECDVPEHCTGQSAECPR(D)	4.18	0.00	0.32	791.6647	4.83	1.62%	0.54%
												3	20.17	20.17	85.4	1.29E+05	(R)NECDVPEHCTGQSAECPR(D)	4.28	0.00	0.98	715.9535	4.40		
												3	14.67	14.67	79.2	2.52E+05	(R)KIPCAPQDVK(C)	4.63	5.40	0.99	385.8789	8.59		
												3	18.12	18.12	85.9	6.27E+05	(K)LKPGAECGNGLCCYQCK(I)	4.92	0.00	0.97	672.2926	8.50		
												2	25.76	18.96	94.6	2.15E+05	(K)LKPGAECGNGLCCYQCK(I)	4.92	0.00	0.94	1007.9351	8.50		
												2	17.35	8.47	97.5	1.48E+06	(R)SVGIVQVQGNR(N)	5.10	0.00	1.00	578.8281	9.47		
												2	19.84	19.84	88.2	3.63E+05	(R)LFCLNNSPGNK(N)	5.92	5.40	0.99	632.3140	8.75		
												3	13.26	10.00	81.1	2.05E+05	(K)CILYPPLRK(D)	5.90	0.00	0.98	387.2280	9.99		
												2	12.66	7.05	78.2	7.94E+04	(K)CILYPPLRK(D)	5.90	0.00	1.00	580.3386	9.99		
												2	16.11	16.11	76.1	8.31E+05	(R)NQCISLFGSR(A)	6.62	0.00	0.99	591.2910	9.75		
												2	14.70	7.88	85.4	7.39E+05	(K)CILYPPLR(K)	6.80	0.00	1.00	516.2899	8.75		
3	3.3	3	2	33.23	3.7	1.24E+05	70876.8	5.45	*Macrovipera lebetina*	Q4VM08	Zinc metalloproteinase-disintegrin-like VLAIP-A	3	14.67	14.67	79.2	2.52E+05	(R)KIPCAPQDVK(C)	4.63	5.40	0.99	385.8789	8.59	0.45%	0.15%
												2	18.02	11.04	88.3	1.06E+05	(R)IYEIVNILNVIYR(V)	10.65	5.40	0.99	811.4706	6.00		
												3	18.56	6.31	93.7	1.47E+04	(R)IYEIVNILNVIYR(V)	10.68	0.00	0.78	541.3162	6.00		
3	3.4	2	2	27.51	17.9	1.88E+05	12226.6	9.44	*Naja naja*	Unigene31385_Nn	Zinc metalloproteinase-disintegrin VLAIP-A	3	14.67	14.67	79.2	2.52E+05	(R)KIPCAPQDVK(C)	4.63	5.40	0.99	385.8789	8.59	0.68%	0.23%
												2	12.84	12.84	76.7	1.24E+05	(K)QCISLFGSR(A)	6.65	5.40	0.99	534.2685	9.75		
4	4.1	9	6	125.13	51.4	2.34E+06	16719.9	9.60	*Daboia russelii*	A8CG89	Basic phospholipase A2 Drk-b1	2	20.62	20.62	77.4	3.02E+06	(R)CCFVHDCCYAR(V)	4.88	5.40	0.97	774.2884	6.74	8.45%	2.82%
												2	18.16	10.78	92.4	3.67E+06	(K)LVEYSYSYR(T)	5.60	5.40	0.96	590.2912	6.00		
												2	21.59	14.50	94.0	4.47E+06	(R)VAAICLGQNVNTYNK(G)	6.15	10.81	0.94	832.9290	8.56		
												3	17.00	7.60	88.9	6.66E+05	(R)VAAICLGQNVNTYNK(G)	6.15	5.40	0.99	555.6207	8.56		
												2	25.83	25.83	97.6	4.48E+06	(K)YISYGCYCGWGGQGTPK(D)	6.58	5.40	0.96	977.4203	8.43		
												3	18.08	18.08	89.8	9.09E+05	(K)YISYGCYCGWGGQGTPK(D)	6.58	10.81	1.00	651.9480	8.43		
												2	17.97	14.26	80.5	3.39E+06	(K)GYMFLSSYYCR(Q)	7.90	10.81	0.98	723.8159	8.50		
												3	10.98	10.98	70.2	1.91E+04	(K)GYMFLSSYYCR(Q)	7.90	5.40	0.95	482.8759	8.50		
												3	20.96	20.96	92.8	4.60E+05	(K)QEAFSFFKYISYGCYCGWGGQGTPK(D)	9.23	0.00	0.98	980.1064	8.38		
4	4.2	9	3	63.51	43.4	1.00E+06	16441.3	4.93	*Daboia siamensis*	A8CG78	Acidic phospholipase A2 DsM-a2	2	18.08	18.08	83.2	2.58E+06	(K)YMLYSIFDCKEESDQC(-)	8.35	16.35	0.97	1044.4262	3.92	3.61%	1.20%
												3	11.30	11.30	70.3	9.93E+05	(K)YMLYSIFDCKEESDQC(-)	8.37	16.35	0.97	696.6203	3.92		
												3	22.67	22.67	88.9	2.37E+06	(K)TGNFGLLSYVYYGCYCGWGGK(G)	9.70	16.21	0.99	808.0288	8.05		
												2	19.95	19.95	90.1	2.19E+06	(K)TGNFGLLSYVYYGCYCGWGGK(G)	9.68	16.21	0.98	1211.5381	8.05		
												3	22.76	22.76	93.5	6.78E+04	(K)TATYSYSFENGDIVCGGDDPCLR(A)	11.03	9.54	0.90	866.3705	3.84		
												3	21.19	21.19	91.5	7.59E+05	(K)TATYSYSFENGDIVCGGDDPCLR(A)	12.47	35.58	0.91	866.3705	3.84		
												3	14.28	14.28	75.1	2.66E+04	(K)TGNFGLLSYVYYGCYCGWGGK(G)	14.23	0.26	0.98	808.0264	8.05		
												3	20.37	20.37	89.4	1.04E+04	(K)TATYSYSFENGDIVCGGDDPCLR(A)	14.72	0.00	0.86	866.3705	3.84		
												3	13.56	13.56	75.2	2.11E+04	(K)TGNFGLLSYVYYGCYCGWGGK(G)	15.13	1.15	0.96	808.0264	8.05		
5	5.1	13	6	101.25	72.4	1.04E+06	16251.0	4.65	*Daboia siamensis*	Q7T2R1	Acidic phospholipase A2 daboiatoxin A chain	2	21.58	21.58	90.2	1.07E+06	(R)CCFVHDCCYGTVNDCNPK(M)	5.18	0.00	0.92	1153.4355	5.21	3.76%	1.25%
												2	14.75	14.75	84.6	2.75E+06	(K)NYENYAISHCTEESEQC(-)	5.38	5.41	0.98	1067.3964	4.09		
												3	17.59	11.74	84.2	8.61E+05	(R)AAAICLGQNVNTYDK(N)	6.02	5.40	0.99	546.6053	5.88		
												4	15.32	15.32	83.8	5.46E+06	(K)EAVHSYAIYGCYCGWGGQGKPQDATDR(C)	6.37	10.81	0.97	762.3395	5.38		
												3	24.95	24.95	100.0	2.34E+06	(K)EAVHSYAIYGCYCGWGGQGKPQDATDR(C)	6.37	10.81	0.99	1016.1155	5.38		
												2	17.34	17.34	84.1	5.70E+05	(R)AAAICLGQNVNTYDK(N)	8.35	156.85	0.95	819.4021	5.88		
												3	20.41	20.41	92.6	4.47E+04	(K)MATYSYSFENGDIVCGDNNLCLK(T)	9.05	0.00	0.41	891.0529	4.03		
												3	22.38	22.38	94.0	3.91E+04	(K)MATYSYSFENGDIVCGDNNLCLK(T)	11.28	0.00	0.65	891.0529	4.03		
												2	15.16	15.16	74.4	3.85E+05	(R)AAAICLGQNVNTYDK(N)	12.38	35.51	0.91	819.4021	5.88		
												3	18.09	18.09	76.4	1.45E+04	(K)MATYSYSFENGDIVCGDNNLCLK(T)	14.97	0.00	0.88	891.0548	4.03		
												2	11.16	11.16	71.1	1.11E+04	(-)NFFQFAEMIVK(M)	13.28	0.58	0.95	687.3503	6.00		
												4	14.88	14.88	72.6	6.05E+03	(K)EAVHSYAIYGCYCGWGGQGKPQDATDR(C)	14.38	0.00	0.28	762.3373	5.38		
												3	17.63	17.63	78.8	1.09E+04	(K)MATYSYSFENGDIVCGDNNLCLK(T)	15.82	0.00	0.90	891.0548	4.03		
5	5.2	13	5	98.97	68.1	7.96E+05	16256.9	4.61	*Daboia siamensis*	Q7ZZQ1	phospholipase A2-I	2	21.58	21.58	90.2	1.07E+06	(R)CCFVHDCCYGTVNDCNPK(M)	5.18	0.00	0.92	1153.4355	5.21	2.87%	0.96%
												2	14.75	14.75	84.6	2.75E+06	(K)NYENYAISHCTEESEQC(-)	5.38	5.41	0.98	1067.3964	4.09		
												3	17.59	11.74	84.2	8.61E+05	(R)AAAICLGQNVNTYDK(N)	6.02	5.40	0.99	546.6053	5.88		
												2	17.34	17.34	84.1	5.70E+05	(R)AAAICLGQNVNTYDK(N)	8.35	156.85	0.95	819.4021	5.88		
												3	22.67	22.67	88.9	2.37E+06	(K)TGNFGLLSYVYYGCYCGWGGK(G)	9.70	16.21	0.99	808.0288	8.05		
												2	19.95	19.95	90.1	2.19E+06	(K)TGNFGLLSYVYYGCYCGWGGK(G)	9.68	16.21	0.98	1211.5381	8.05		
												3	20.41	20.41	92.6	4.47E+04	(K)MATYSYSFENGDIVCGDNNLCLK(T)	9.05	0.00	0.41	891.0529	4.03		
												3	22.38	22.38	94.0	3.91E+04	(K)MATYSYSFENGDIVCGDNNLCLK(T)	11.28	0.00	0.65	891.0529	4.03		
												2	15.16	15.16	74.4	3.85E+05	(R)AAAICLGQNVNTYDK(N)	12.38	35.51	0.91	819.4021	5.88		

												3	14.28	14.28	75.1	2.66E+04	(K)TGNFGLLSYVYYGCYCGWGGK(G)	14.23	0.26	0.98	808.0264	8.05		
												3	18.09	18.09	76.4	1.45E+04	(K)MATYSYSFENGDIVCGDNNLCLK(T)	14.97	0.00	0.88	891.0548	4.03		
												3	13.56	13.56	75.2	2.11E+04	(K)TGNFGLLSYVYYGCYCGWGGK(G)	15.13	1.15	0.96	808.0264	8.05		
												3	17.63	17.63	78.8	1.09E+04	(K)MATYSYSFENGDIVCGDNNLCLK(T)	15.82	0.00	0.90	891.0548	4.03		
6	6.1	7	6	102.71	30.0	8.09E+05	29507.5	9.78	*Daboia siamensis*	P18965	Factor V activator RVV-V gamma	2	13.19	13.19	71.1	5.56E+05	(K)YFCLNTK(F)	5.67	5.40	1.00	473.2301	8.59	2.92%	0.97%
												2	13.35	13.35	82.7	6.11E+05	(R)EWVLTAAHCDR(R)	5.83	5.40	1.00	679.3212	5.32		
												3	14.91	14.91	85.7	7.73E+05	(R)RPVTYSTHIAPVSLPSR(S)	5.90	0.00	0.99	627.6862	10.84		
												3	17.70	17.70	87.3	5.05E+05	(K)FPNGLDKDIMLIR(L)	7.97	0.00	0.99	511.2835	5.96		
												2	18.86	18.86	91.1	2.67E+06	(K)WCEPLYPWVPADSR(T)	8.50	5.40	0.99	888.4181	4.37		
												3	20.99	16.21	95.6	4.93E+05	(K)WCEPLYPWVPADSR(T)	8.50	5.40	0.98	592.6132	4.37		
												2	22.57	22.57	98.2	5.42E+04	(K)VFDYNNWIQSIIAGNR(T)	9.87	0.00	0.96	955.4830	5.80		
6	6.2	7	5	72.73	24.3	7.16E+05	29126.9	7.94	*Daboia russelii*	CL2958.contig6_DrSL	Serine protease VLSP-1	2	14.63	14.63	78.3	9.74E+05	(K)IYDYSVCR(A)	5.10	0.00	1.00	538.2494	5.83	2.58%	0.86%
												2	15.13	15.13	74.7	2.55E+05	(R)FFCLSNK(N)	6.15	5.40	1.00	458.2244	8.75		
												3	15.46	15.46	82.9	5.12E+05	(R)HPCAQAELPAFYTK(V)	6.27	0.00	0.99	544.9338	6.75		
												3	22.55	12.85	96.6	7.86E+05	(R)LNSPVTYNTHIAPFSLPSSPPTVGSVCR(I)	8.07	0.00	1.00	1000.1771	8.75		
												3	18.15	18.15	81.6	1.28E+06	(K)SFTPWDKDIMLIR(L)	8.52	0.00	1.00	541.2869	5.68		
												2	20.09	20.09	84.0	6.03E+05	(K)SFTPWDKDIMLIR(L)	8.52	0.00	1.00	811.4258	5.68		
												2	10.81	3.99	70.2	6.03E+05	(K)SFTPWDKDIMLIR(L)	8.52	0.00	1.00	811.4258	5.68		
6	6.3	4	3	51.13	17.5	7.27E+05	28719.5	7.07	*Daboia siamensis*	E5L0E4	Beta-fibrinogenase-like	2	15.13	15.13	74.7	2.55E+05	(R)FFCLSNK(N)	6.15	5.40	1.00	458.2244	8.75	2.62%	0.87%
												2	17.83	12.52	96.9	1.78E+06	(K)TSTYIAPLSLPSSPPR(V)	7.35	5.41	0.99	843.9607	8.41		
												3	19.33	11.28	97.4	3.24E+05	(K)TSTYIAPLSLPSSPPR(V)	7.35	5.41	0.96	562.9757	8.41		
												3	16.67	10.62	86.9	5.47E+05	(K)VYDYTDWIQSIIAGNTAATCPP(-)	10.40	0.00	1.00	819.3887	3.56		
7	7.1	5	5	91.81	33.3	1.19E+06	18523.6	6.31	*Daboia siamensis*	Q4PRC6	Snaclec 7	2	17.82	12.57	76.9	1.51E+06	(K)TTDNQWLR(W)	5.38	5.41	0.99	517.2504	5.50	4.30%	1.43%
												2	20.20	20.20	97.2	5.84E+05	(R)SSEEMDFVIR(M)	6.90	5.40	1.00	606.7841	4.14		
												2	22.61	22.61	94.4	2.21E+05	(K)QDCLSDWSFYEGYCYK(V)	8.18	5.53	1.00	1060.9232	4.03		
												2	13.79	13.79	74.7	2.43E+06	(R)MTFPIFR(F)	8.25	0.13	0.98	456.2456	9.50		
												2	17.39	17.39	85.5	1.21E+06	(R)FDFFWIGLR(D)	10.48	10.81	0.97	600.8161	5.84		
7	7.2	7	3	63.73	39.1	1.92E+05	17529.7	6.58	*Daboia siamensis*	Q4PRC8	Snaclec 5	4	10.89	10.89	83.2	4.03E+05	(K)GSHLLSLHNIAEADFVLK(K)	8.00	10.81	0.99	491.7726	5.99	0.69%	0.23%
												3	15.06	15.06	89.8	3.90E+05	(K)GSHLLSLHNIAEADFVLK(K)	8.00	10.81	0.97	655.3606	5.99		
												2	19.17	11.79	100.0	1.45E+05	(K)GSHLLSLHNIAEADFVLK(K)	7.98	5.40	0.98	982.5358	5.99		
												2	22.61	22.61	94.4	2.21E+05	(K)QDCLSDWSFYEGYCYK(V)	8.18	5.53	1.00	1060.9232	4.03		
												3	21.95	21.95	94.7	1.18E+05	(K)DGVIWMGLNDVWNECNWGWTDGAK(L)	10.43	16.21	0.99	941.7484	3.84		
												2	21.82	21.82	86.1	5.99E+04	(K)DGVIWMGLNDVWNECNWGWTDGAK(L)	10.42	16.21	0.89	1412.1182	3.84		
												4	11.34	11.34	76.7	6.53E+03	(K)GSHLLSLHNIAEADFVLK(K)	11.18	0.00	0.60	491.7713	5.99		
8	8.1	6	5	83.11	8.9	4.68E+04	98156.9	7.36	*Daboia russelii*	CL3655.contig2_DrSL	phosphodiesterase 1	2	16.10	16.10	91.0	6.00E+04	(K)AATYFWPGSEVK(I)	7.30	5.40	0.99	678.3362	6.04	0.17%	0.06%
												2	13.11	6.64	86.6	4.68E+04	(R)TLGMLMEGLK(Q)	7.93	5.40	0.99	546.7938	5.66		
												3	10.57	3.11	70.7	8.01E+04	(R)VRDVELLTGLNFYSGLK(Q)	8.78	0.00	0.99	642.0248	6.04		
												3	17.88	17.88	71.2	2.34E+04	(K)FGPVSGEIIMALQMADR(T)	9.88	5.40	0.96	612.3138	4.37		
												2	22.61	22.61	96.4	3.08E+04	(K)FGPVSGEIIMALQMADR(T)	9.88	5.40	0.98	917.9637	4.37		
												3	20.72	11.49	100.0	3.98E+04	(K)NEVTSFENIEVYNLMCDLLK(L)	11.10	4.13	0.98	811.0573	4.00		
9	9.1	6	4	70.59	68.3	3.31E+06	7191.8	9.69	*Daboia siamensis*	P00990	Kunitz-type serine protease inhibitor 2	2	13.23	3.95	88.5	2.55E+06	(R)RIYYNLESNK(C)	5.00	0.00	0.94	650.3426	8.50	11.93%	3.98%
												3	13.09	13.09	83.4	1.80E+06	(R)RIYYNLESNK(C)	5.00	0.00	0.98	433.8980	8.50		
												4	15.29	15.29	86.3	4.82E+06	(-)HDRPTFCNLAPESGR(C)	5.13	5.40	1.00	439.9628	6.75		
												2	17.46	12.11	98.4	3.68E+06	(-)HDRPTFCNLAPESGR(C)	5.13	5.40	1.00	878.9174	6.75		
												2	18.89	18.89	89.1	4.79E+06	(R)IYYNLESNK(C)	5.40	5.41	0.91	572.2838	6.00		
												3	21.01	21.01	90.3	2.20E+06	(K)VFFYGGCGGNANNFETR(D)	6.87	16.21	0.94	637.2832	5.97		
9	9.2	5	3	61.62	39.2	2.20E+06	9712.8	6.82	*Daboia siamensis*	A8Y7P4	Kunitz-type serine protease inhibitor B4	2	13.23	3.95	88.5	2.55E+06	(R)RIYYNLESNK(C)	5.00	0.00	0.94	650.3426	8.50	7.92%	2.64%
												3	13.09	13.09	83.4	1.80E+06	(R)RIYYNLESNK(C)	5.00	0.00	0.98	433.8980	8.50		
												2	18.89	18.89	89.1	4.79E+06	(R)IYYNLESNK(C)	5.40	5.41	0.91	572.2838	6.00		
												3	21.33	21.33	90.5	6.50E+05	(K)CEVFFYGGCGGNDNNFSTWDECR(H)	8.07	0.00	0.95	931.0278	3.91		
												2	29.50	29.50	100.0	1.19E+06	(K)CEVFFYGGCGGNDNNFSTWDECR(H)	8.12	10.93	0.86	1396.0361	3.91		
10	10.1	4	3	66.44	28.7	5.67E+05	17267.5	5.63	*Daboia siamensis*	Q4PRC9	Snaclec 4	2	24.56	24.56	96.0	7.28E+05	(K)VFTEEMNWADAEK(F)	6.98	0.00	0.98	785.3509	4.00	2.05%	0.68%
												2	19.44	19.44	85.2	3.19E+05	(K)SMTCNFIAPVVCK(F)	6.98	0.00	0.99	763.8641	8.47		
												3	22.44	12.32	92.5	1.05E+06	(K)ALAEESYCLIMITHEK(V)	7.63	5.40	1.00	636.6481	4.75		
												2	20.59	20.59	82.7	1.72E+05	(K)ALAEESYCLIMITHEK(V)	7.62	0.00	0.91	954.4676	4.75		
10	10.2	7	3	65.68	37.1	2.64E+05	17366.4	6.03	*Daboia siamensis*	Q4PRD0	Snaclec 3	2	24.56	24.56	96.0	7.28E+05	(K)VFTEEMNWADAEK(F)	6.98	0.00	0.98	785.3509	4.00	0.95%	0.32%
												4	10.89	10.89	83.2	4.03E+05	(K)GSHLLSLHNIAEADFVLK(K)	8.00	10.81	0.99	491.7726	5.99		
												3	15.06	15.06	89.8	3.90E+05	(K)GSHLLSLHNIAEADFVLK(K)	8.00	10.81	0.97	655.3606	5.99		
												2	19.17	11.79	100.0	1.45E+05	(K)GSHLLSLHNIAEADFVLK(K)	7.98	5.40	0.98	982.5358	5.99		
												3	21.95	21.95	94.7	1.18E+05	(K)DGVIWMGLNDVWNECNWGWTDGAK(L)	10.43	16.21	0.99	941.7484	3.84		
												2	21.82	21.82	86.1	5.99E+04	(K)DGVIWMGLNDVWNECNWGWTDGAK(L)	10.42	16.21	0.89	1412.1182	3.84		
												4	11.34	11.34	76.7	6.53E+03	(K)GSHLLSLHNIAEADFVLK(K)	11.18	0.00	0.60	491.7713	5.99		
11	11.1	6	4	65.93	29.8	2.76E+05	17905.9	7.07	*Daboia siamensis*	Q38L02	Snaclec dabocetin subunit alpha	3	21.70	21.70	95.4	3.25E+05	(R)QQCSSHWTDGSAVSYETVTK(Y)	5.68	5.40	0.99	757.6751	5.32	1.00%	0.33%
												2	20.70	20.70	100.0	1.41E+05	(R)QQCSSHWTDGSAVSYETVTK(Y)	5.70	5.40	0.99	1136.0073	5.32		
												2	12.02	12.02	73.3	3.94E+05	(K)YHAWIGLR(D)	6.17	0.00	0.99	508.2780	8.75		
												3	14.50	9.81	85.9	8.57E+04	(K)YHEWITLPCGDK(N)	6.73	5.40	1.00	506.9070	5.32		
												3	17.71	11.11	90.2	3.57E+05	(K)YHEWITLPCGDKNPFICK(S)	7.80	0.00	1.00	760.0340	6.75		
												4	12.81	4.83	76.7	3.54E+05	(K)YHEWITLPCGDKNPFICK(S)	7.80	0.00	1.00	570.2776	6.75		
12	12.1	3	3	54.79	10.9	1.44E+05	55972.0	7.01	*Ovophis okinavensis*	U3T7C6	5'-nucleotidase	3	17.55	12.23	93.5	2.62E+05	(K)IIALGHSGFFEDQR(I)	7.08	32.42	0.97	530.6095	5.32	0.52%	0.17%
												3	19.67	12.26	89.7	1.08E+05	(K)YLGYLNVIFDDKGNVIK(A)	9.05	0.00	0.99	657.6928	5.96		
												3	17.57	7.82	87.5	6.30E+04	(R)YDAMALGNHEFDNGLAGLLDPLLK(H)	10.40	0.00	0.74	863.1016	4.22		
12	12.2	3	3	50.48	7.4	1.48E+05	63610.7	9.15	*Daboia russelii*	CL3322.contig1_DrSL	Snake venom 5'-nucleotidase	3	13.26	9.00	77.8	7.34E+04	(K)VVYDLSQKPGSR(V)	4.83	5.40	0.98	450.2465	8.56	0.53%	0.18%
												3	17.55	12.23	93.5	2.62E+05	(K)IIALGHSGFFEDQR(I)	7.08	32.42	0.97	530.6095	5.32		
												3	19.67	12.26	89.7	1.08E+05	(K)YLGYLNVIFDDKGNVIK(A)	9.05	0.00	0.99	657.6928	5.96		
13	13.1	3	3	44.44	16.4	3.35E+05	18793.1	5.84	*Daboia siamensis*	Q4PRD2	Snaclec coagulation factor X-activating enzyme light chain 2	2	11.33	1.70	76.0	3.54E+05	(K)CFVLEK(E)	5.37	0.00	1.00	398.1993	6.00	1.21%	0.40%
												2	19.31	14.41	93.4	1.98E+05	(-)LDCPPDSSLYR(Y)	5.55	0.00	0.98	661.8084	4.21		
												2	13.80	13.80	76.3	4.52E+05	(R)YLFVCK(V)	6.05	5.40	1.00	415.2194	8.59		
14	14.1	3	2	43.62	17.0	1.37E+06	18452.4	5.38	*Daboia siamensis*	K9JBV0	P68 alpha subunit	2	23.45	19.53	100.0	1.97E+06	(K)TPADYVWIGLR(N)	8.33	0.00	0.97	645.8487	5.50	4.94%	1.65%
												3	14.49	14.49	84.3	3.98E+04	(K)TPADYVWIGLR(N)	8.33	0.00	0.99	430.9001	5.50		
												2	20.17	20.17	91.3	2.10E+06	(R)TWFNLSCGDDYPFVCK(F)	8.87	10.81	0.99	1004.9344	4.21		
15	15.1	3	2	43.60	21.3	4.80E+05	13625.1	9.69	*Daboia russelii*	P30894	Venom nerve growth factor	2	22.55	22.55	95.6	9.80E+05	(R)INTACVCVISR(K)	5.72	0.00	0.99	646.8285	9.75	1.73%	0.58%
												2	21.05	15.41	87.2	2.48E+05	(K)HWNSYCTTTDTFVR(A)	5.90	0.00	1.00	894.3984	6.74		
												3	18.16	18.16	75.6	2.11E+05	(K)HWNSYCTTTDTFVR(A)	5.90	0.00	0.98	596.6009	6.74		
16	16.1	4	2	42.03	6.1	3.56E+05	55887.4	5.44	*Daboia russelii*	CL3662.contig2_DrSL	Zinc metalloproteinase-disintegrin VLAIP-A	3	17.03	17.03	85.3	8.34E+05	(K)LTQGSQCADEECCDQCK(F)	3.98	3.76	0.91	696.9306	3.91	1.28%	0.43%
												2	23.47	23.47	92.4	4.68E+05	(K)LTQGSQCADEECCDQCK(F)	3.98	3.76	0.93	1044.8912	3.91		
												2	18.02	11.04	88.3	1.06E+05	(R)IYEIVNILNVIYR(V)	10.65	5.40	0.99	811.4706	6.00		
												3	18.56	6.31	93.7	1.47E+04	(R)IYEIVNILNVIYR(V)	10.68	0.00	0.78	541.3162	6.00		
17	17.1	2	2	39.51	15.8	1.19E+05	18504.6	6.41	*Daboia siamensis*	K9JBU9	P31 alpha subunit	2	17.86	17.86	95.2	1.28E+05	(K)IIYVNWK(E)	6.72	0.00	1.00	468.2713	8.59	0.43%	0.14%
												2	21.65	21.65	95.8	1.09E+05	(K)SVGEANFVAQLASGFMQK(D)	9.80	5.40	0.99	942.4707	5.72		
18	18.1	3	2	37.06	27.7	1.29E+06	10243.6	9.57	*Daboia siamensis*	A8Y7P5	Kunitz-type serine protease inhibitor B5	2	15.19	6.68	79.7	8.36E+05	(R)SFYYDSESK(K)	5.00	0.00	1.00	563.2434	4.37	4.66%	1.55%
												3	17.82	17.82	76.0	2.61E+06	(K)FCYLPADPGECLAHMR(S)	7.47	10.81	0.97	646.2907	5.32		
												2	21.87	21.87	87.0	4.25E+05	(K)FCYLPADPGECLAHMR(S)	7.47	10.81	0.99	968.9318	5.32		
19	19.1	2	2	34.00	5.5	9.11E+04	77077.2	5.92	*Boiga*	A0A0B8RN	Xaa-Pro aminopeptidase	3	14.17	14.17	82.2	1.29E+05	(R)GDDIPYTPVFYAYTLLTK(T)	11.03	0.00	-0.83	693.0249	4.21	0.33%	0.11%

									irregularis	S9	2													
												3	19.83	19.83	91.4	5.31E+04	(K)KPTAILLSGLEETAWLFNLR(G)	11.08	8.77	0.97	758.0954	6.14		
19	19.2	2	2	32.21	5.4	8.79E+04	74587.3	5.63	*Daboia russelii*	Unigene32033_DrSL	xaa-Pro aminopeptidase 2-like	3	14.17	14.17	82.2	1.29E+05	(R)GDDIPYTPVFYAYTLLTK(T)	11.03	0.00	-0.83	693.0249	4.21	0.32%	0.11%
												3	18.04	10.26	92.5	4.67E+04	(R)TLDLLEVNLVDLVWGSER(L)	11.52	7.97	0.91	691.0412	3.91		
20	20.1	2	2	33.24	15.7	2.30E+05	15889.7	9.56	*Daboia russelii*	Unigene30367_DrSL	C-type lectin A12	2	18.08	18.08	77.1	2.64E+05	(K)TWEDAETFCQK(Q)	5.92	5.40	0.99	707.8100	4.14	0.83%	0.28%
												2	15.16	8.32	85.6	1.96E+05	(K)HLATIEWLGK(A)	6.85	10.81	1.00	584.3307	6.75		
20	20.2	3	2	31.37	14.1	2.06E+05	18116.4	9.16	*Macrovipera lebetina*	B4XSY7	Snaclec A12	2	15.16	8.32	85.6	1.96E+05	(K)HLATIEWLGK(A)	6.85	10.81	1.00	584.3307	6.75	0.74%	0.25%
												2	16.21	16.21	80.0	3.88E+05	(K)ANFVAELVTLMK(L)	10.75	5.40	1.00	668.3723	6.05		
												3	14.26	8.00	88.1	3.48E+04	(K)ANFVAELVTLMK(L)	10.75	5.40	1.00	445.9170	6.05		
21	21.1	2	1	28.94	6.9	7.35E+04	29577.7	9.77	*Macrovipera lebetina*	Q9PT40	Venom serine proteinase-like protein 2	2	28.94	28.94	95.9	8.59E+04	(R)FYCAGTLINQEWVLTAAR(C)	9.02	10.81	0.99	1057.0309	6.00	0.27%	0.09%
												3	17.94	17.94	80.6	6.11E+04	(R)FYCAGTLINQEWVLTAAR(C)	9.05	16.21	0.96	705.0224	6.00		
22	22.1	2	1	25.36	24.7	1.59E+06	13010.7	7.20	*Daboia siamensis*	P0DL42	Snake venom vascular endothelial growth factor toxin VR-1	3	25.36	20.45	98.5	2.91E+06	(R)ETLVSILQEHPDEISDIFRPSCVAVLR(C)	9.47	5.41	1.00	1041.8811	4.50	5.75%	1.92%
												5	14.79	4.99	82.5	2.75E+05	(R)ETLVSILQEHPDEISDIFRPSCVAVLR(C)	9.45	5.41	0.99	625.5318	4.50		
23	23.1	2	1	24.05	80.0	2.77E+05	1698.8	4.65	*Daboia russelii*	P86531	Vipera russelli proteinase RVV-V homolog 2	3	20.37	20.37	93.6	4.89E+05	(-)VVGGDECNINEHR(F)	4.22	5.40	0.92	500.2305	4.65	1.00%	0.33%
												2	24.05	17.27	96.9	6.57E+04	(-)VVGGDECNINEHR(F)	4.22	5.40	0.89	749.8403	4.65		
24	24.1	1	1	23.11	5.8	4.63E+05	28981.6	7.11	*Macrovipera lebetina*	E0Y419	Beta-fibrinogenase	2	23.11	23.11	94.1	4.63E+05	(R)TLCAGILQGGIDTCK(G)	6.83	5.40	0.98	803.9022	5.50	1.67%	0.56%
25	25.1	2	1	21.67	6.6	2.61E+05	27826.7	5.61	*Gloydius blomhoffii*	Q8JI40	Cysteine-rich venom protein ablomin	4	11.55	1.83	81.5	2.62E+05	(R)KPEIQNEIVDLHNSLR(R)	6.58	5.40	0.99	477.0109	5.45	0.94%	0.31%
												3	21.67	14.32	100.0	2.60E+05	(R)KPEIQNEIVDLHNSLR(R)	6.58	5.40	0.98	635.6789	5.45		
						2.77E+07																	100.00%	33.33%

Daboia siamensis Guangxi venom 3

Group	Subgroup	Spectra	Distinct Peptides	Distinct Summed MS/MS Search Score	% AA Coverage	Mean Protein Spectral Intensity	Protein MW (Da)	Protein pI	Species	Database Accession	Protein Name	z	Score	Fwd-Rev Score	SPI (%)	Spectrum Intensity	Sequence	RT (min)	Peak Width (sec)	Average Chi Squared	m/z Measured (Da)	Peptide pI	Relative abundance (n=1)	Relative abundance (% overall)
1	1.1	20	12	205.40	26.0	8.69E+05	71721.7	5.95	*Daboia russelii*	B8K1W0	Zinc metalloproteinase-disintegrin-like daborhagin-K	4	11.90	11.90	75.1	3.47E+05	(R)AGTECRPARDECDKAEQCTGR(S)	3.88	4.09	0.98	617.5186	4.94	3.01%	1.00%
												3	27.55	27.55	97.6	3.70E+05	(R)GEECDCGSPENCRDPCCDAASCK(L)	4.17	0.00	0.87	911.9871	4.02		
												2	25.22	25.22	91.4	1.75E+04	(R)GEECDCGSPENCRDPCCDAASCK(L)	4.17	0.00	0.90	1367.4725	4.02		
												4	12.83	12.83	74.2	1.05E+05	(R)GEECDCGSPENCRDPCCDAASCK(L)	4.25	5.40	0.91	684.2414	4.02		
												2	20.13	20.13	77.4	2.70E+06	(K)LHSWVECESGK(C)	4.82	5.40	0.95	666.3084	5.40		
												3	16.65	16.65	84.6	2.74E+06	(K)LHSWVECESGK(C)	4.82	5.40	0.95	444.5423	5.40		
												2	15.78	8.15	89.7	6.50E+05	(K)YKNDLTAIR(T)	4.87	10.81	0.99	547.3051	8.59		
												4	14.26	14.26	88.6	3.02E+05	(R)RFLTEHNPECIINPPLR(T)	6.52	0.00	1.00	527.2800	6.76		
												3	14.27	14.27	71.6	2.99E+05	(R)RFLTEHNPECIINPPLR(T)	6.52	0.00	1.00	702.7039	6.76		
												3	15.78	15.78	70.5	5.91E+05	(R)TDIVSPPACGNELLER(G)	6.68	0.00	0.98	590.9632	4.14		
												2	23.19	17.22	98.4	2.95E+06	(R)FLTEHNPECIINPPLR(T)	6.92	5.41	1.00	975.5034	5.40		
												4	13.54	13.54	77.6	1.17E+05	(R)FLTEHNPECIINPPLR(T)	6.90	10.81	0.89	488.2549	5.40		
												2	14.69	14.69	90.3	2.20E+05	(K)VCSNGQCVDLNIAY(-)	7.70	5.40	1.00	806.8602	3.80		
												2	14.92	14.92	88.3	2.56E+06	(K)SPGNQIPCLPYYIPSDENK(G)	7.68	0.00	0.98	1096.5253	4.37		
												3	14.43	9.79	95.2	8.90E+05	(K)SPGNQIPCLPYYIPSDENK(G)	7.68	0.00	1.00	731.3520	4.37		
												3	13.56	7.71	91.1	7.87E+04	(R)VPLVGLEIWK(N)	8.93	10.81	0.99	385.2387	5.97		
												2	18.51	12.78	74.9	8.06E+04	(R)TWVFELVNTINEIFK(Y)	12.87	0.13	0.99	926.9956	4.53		
												3	13.61	4.41	79.6	2.33E+06	(R)TWVFELVNTINEIFKYLYIR(V)	14.43	15.92	0.99	854.4668	5.81		
												4	13.28	8.28	78.5	1.36E+04	(R)TWVFELVNTINEIFKYLYIR(V)	14.47	0.00	0.89	641.1006	5.81		
												2	15.12	15.12	70.2	2.47E+04	(R)TWVFELVNTINEIFKYLYIR(V)	14.47	0.26	0.81	1281.1923	5.81		
2	2.1	15	11	196.56	27.9	3.76E+05	57286.2	8.82	*Daboia russelii*	G8XQX1	L-amino-acid oxidase	3	14.02	8.05	74.0	6.32E+05	(K)VTVLEASERPGGR(V)	4.85	5.40	0.98	457.5861	6.11	1.30%	0.43%
												2	16.83	10.54	97.6	1.49E+05	(K)VTVLEASERPGGR(V)	4.85	5.40	0.95	685.8751	6.11		
												2	19.09	10.76	91.1	8.89E+05	(K)SAGQLYQESLGK(A)	5.42	5.41	0.99	640.8311	5.72		
												3	13.18	4.81	81.4	2.30E+05	(R)ITFKPPLPPK(K)	6.25	16.21	1.00	379.9066	10.00		
												2	14.98	8.07	86.9	1.49E+05	(K)KDLQTFCYPSIIQK(W)	6.77	5.40	0.99	870.9567	8.50		
												2	17.07	17.07	81.3	6.58E+05	(K)EGWYANLGPMR(V)	7.23	0.13	1.00	647.3080	6.10		
												2	18.41	12.35	88.9	2.20E+05	(K)DLQTFCYPSIIQK(W)	7.70	5.40	1.00	806.9077	5.83		
												3	18.28	18.28	89.0	6.75E+04	(K)RFDEIVGGMDQLPTSMYR(A)	7.87	0.00	0.99	705.6725	4.56		
												2	18.95	18.95	86.5	9.84E+04	(R)FDEIVGGMDQLPTSMYR(A)	8.08	10.94	0.92	979.9537	4.03		
												3	14.26	4.59	79.3	8.34E+05	(R)IFFAGEYTANAHGWIDSTIK(S)	8.42	32.55	0.99	747.7063	5.32		
												2	23.05	16.62	97.0	4.29E+05	(K)LNEFVQETENGWYFIK(N)	8.97	10.81	0.96	1008.9900	4.25		
												3	20.86	20.86	88.2	8.13E+04	(K)YAMGAITTFTPYQFQHFSEALTAPVGR(I)	9.33	5.40	0.97	1002.1627	6.75		
												3	15.86	5.51	80.8	1.18E+06	(R)IFFAGEYTANAHGWIDSTIK(S)	11.77	175.15	0.99	747.7047	5.32		
												3	11.34	11.34	74.2	1.10E+04	(R)IFFAGEYTANAHGWIDSTIK(S)	13.12	0.00	0.98	747.7047	5.32		
												3	13.66	5.56	83.9	7.29E+03	(R)IFFAGEYTANAHGWIDSTIK(S)	15.07	0.00	0.97	747.7047	5.32		
2	2.2	14	10	182.92	32.9	7.28E+05	46713.1	7.90	*Daboia siamensis*	Q4F867	L-amino-acid oxidase	2	19.09	10.76	91.1	8.89E+05	(K)SAGQLYQESLGK(A)	5.42	5.41	0.99	640.8311	5.72	2.52%	0.84%
												3	13.18	4.81	81.4	2.30E+05	(R)ITFKPPLPPK(K)	6.25	16.21	1.00	379.9066	10.00		
												2	14.98	8.07	86.9	1.49E+05	(K)KDLQTFCYPSIIQK(W)	6.77	5.40	0.99	870.9567	8.50		
												2	18.41	12.35	88.9	2.20E+05	(K)DLQTFCYPSIIQK(W)	7.70	5.40	1.00	806.9077	5.83		
												3	18.28	18.28	89.0	6.75E+04	(K)RFDEIVGGMDQLPTSMYR(A)	7.87	0.00	0.99	705.6725	4.56		
												2	18.95	18.95	86.5	9.84E+04	(R)FDEIVGGMDQLPTSMYR(A)	8.08	10.94	0.92	979.9537	4.03		
												3	14.26	4.59	79.3	8.34E+05	(R)IFFAGEYTANAHGWIDSTIK(S)	8.42	32.55	0.99	747.7063	5.32		
												2	23.05	16.62	97.0	4.29E+05	(K)LNEFVQETENGWYFIK(N)	8.97	10.81	0.96	1008.9900	4.25		
												3	20.86	20.86	88.2	8.13E+04	(K)YAMGAITTFTPYQFQHFSEALTAPVGR(I)	9.33	5.40	0.97	1002.1627	6.75		
												3	15.86	5.51	80.8	1.18E+06	(R)IFFAGEYTANAHGWIDSTIK(S)	11.77	175.15	0.99	747.7047	5.32		
												3	19.67	12.11	93.5	3.81E+06	(K)NLLLETVDYVIVCTTSR(A)	11.28	15.15	1.00	666.0209	4.37		
												2	20.26	13.01	92.0	2.18E+06	(K)NLLLETVDYVIVCTTSR(A)	11.28	15.15	0.99	998.5260	4.37		
												3	11.34	11.34	74.2	1.10E+04	(R)IFFAGEYTANAHGWIDSTIK(S)	13.12	0.00	0.98	747.7047	5.32		
												3	13.66	5.56	83.9	7.29E+03	(R)IFFAGEYTANAHGWIDSTIK(S)	15.07	0.00	0.97	747.7047	5.32		
2	2.3	6	2	32.00	48.8	5.18E+05	10351.4	5.10	*Vipera berus berus*	P0C2D7	L-amino-acid oxidase	3	14.26	4.59	79.3	8.34E+05	(K)IFFAGEYTANAHGWIDSTIK(-)	8.42	32.55	0.99	747.7063	5.32	1.79%	0.60%
												4	11.92	3.41	89.9	6.61E+05	(-)ADDKNPLEECFREDDYEEFLEIAK(N)	8.62	5.53	1.00	744.5912	3.96		
												3	16.14	16.14	86.2	4.14E+05	(-)ADDKNPLEECFREDDYEEFLEIAK(N)	8.62	5.53	1.00	992.4503	3.96		
												3	15.86	5.51	80.8	1.18E+06	(K)IFFAGEYTANAHGWIDSTIK(-)	11.77	175.15	0.99	747.7047	5.32		
												3	11.34	11.34	74.2	1.10E+04	(K)IFFAGEYTANAHGWIDSTIK(-)	13.12	0.00	0.98	747.7047	5.32		
												3	13.66	5.56	83.9	7.29E+03	(K)IFFAGEYTANAHGWIDSTIK(-)	15.07	0.00	0.97	747.7047	5.32		
3	3.1	10	8	144.02	14.2	2.58E+05	71801.6	6.01	*Daboia russelii*	K9JAW0	factor X activator heavy chain	3	24.20	24.20	100.0	9.73E+04	(R)ARNECDVPEHCTGQSAECPR(D)	4.15	3.12	0.95	791.6650	4.83	0.89%	0.30%
												3	22.23	22.23	81.9	1.27E+05	(R)NECDVPEHCTGQSAECPR(D)	4.27	0.00	0.99	715.9534	4.40		
												3	19.20	19.20	86.4	7.37E+05	(K)LKPGAECGNGLCCYQCK(I)	4.88	0.00	0.96	672.2942	8.50		
												2	11.83	6.07	76.0	4.40E+05	(R)SVGIVQVQGNR(N)	5.07	0.00	0.98	578.8278	9.47		
												3	12.90	8.59	75.4	2.46E+05	(K)CILYPPLRK(D)	5.88	0.00	0.99	387.2202	9.99		
												2	17.80	17.80	86.8	8.44E+04	(K)SHDNALLFTDMR(F)	6.68	0.00	1.00	710.3398	5.19		
												3	16.25	16.25	91.0	6.88E+04	(K)SHDNALLFTDMR(F)	6.68	0.00	0.98	473.8958	5.19		
												2	14.09	7.29	83.3	5.23E+05	(K)CILYPPLR(K)	6.78	0.00	1.00	516.2905	8.75		
												3	21.26	21.26	87.6	1.62E+05	(R)FDLNTLGITFLAGMCQAYR(S)	10.92	10.55	0.99	731.0287	5.83		
												2	21.77	21.77	97.2	9.67E+04	(R)FDLNTLGITFLAGMCQAYR(S)	10.90	10.55	1.00	1096.0382	5.83		
3	3.2	9	8	142.36	12.9	3.46E+05	71793.7	6.05	*Daboia russelii*	Unigene32626_DrSL	factor X activator heavy chain	3	24.20	24.20	100.0	9.73E+04	(R)ARNECDVPEHCTGQSAECPR(D)	4.15	3.12	0.95	791.6650	4.83	1.20%	0.40%
												3	22.23	22.23	81.9	1.27E+05	(R)NECDVPEHCTGQSAECPR(D)	4.27	0.00	0.99	715.9534	4.40		

												3	19.20	19.20	86.4	7.37E+05	(K)LKPGAECGNGLCCYQCK(I)	4.88	0.00	0.96	672.2942	8.50		
												2	11.83	6.07	76.0	4.40E+05	(R)SVGIVQVQGNR(N)	5.07	0.00	0.98	578.8278	9.47		
												2	20.11	20.11	88.6	7.90E+05	(R)LFCLNNSPGNK(N)	5.88	0.00	1.00	632.3008	8.75		
												3	12.90	8.59	75.4	2.46E+05	(K)CILYPPLRK(D)	5.88	0.00	0.99	387.2202	9.99		
												2	17.80	17.80	86.8	8.44E+04	(K)SHDNALLFTDMR(F)	6.68	0.00	1.00	710.3398	5.19		
												3	16.25	16.25	91.0	6.88E+04	(K)SHDNALLFTDMR(F)	6.68	0.00	0.98	473.8958	5.19		
												2	14.09	7.29	83.3	5.23E+05	(K)CILYPPLR(K)	6.78	0.00	1.00	516.2905	8.75		
4	4.1	9	8	140.33	15.7	1.00E+05	98156.9	7.36	*Daboia russelii*	CL3655.contig2_DrSL	phosphodiesterase 1	2	19.49	19.49	88.7	2.30E+05	(K)AATYFWPGSEVK(I)	7.23	0.13	0.99	678.3368	6.04	0.35%	0.12%
												2	14.33	7.67	87.0	4.30E+04	(R)TLGMLMEGLK(Q)	7.87	10.81	0.99	546.7929	5.66		
												3	12.38	12.38	70.1	1.19E+05	(R)LWNYFHGTLLPK(Y)	7.95	0.00	0.99	496.9401	8.60		
												4	15.21	15.21	82.1	7.60E+04	(R)NLHNCVNLILLADHGMEQISCNR(L)	8.32	0.00	0.98	681.0822	5.99		
												2	15.46	10.23	93.8	2.79E+05	(K)TFLPIFVNPVN(-)	9.83	5.40	1.00	630.8548	5.18		
												2	21.50	21.50	79.6	4.58E+04	(K)FGPVSGEIIMALQMADR(T)	9.87	5.40	0.97	917.9636	4.37		
												3	20.69	20.69	89.0	4.48E+04	(K)DQCASSSAAQCPAGFEQSPLILFSMDGFR(A)	10.30	0.00	0.98	1059.8105	4.03		
												3	21.27	13.84	97.1	4.58E+04	(K)NEVTSFENIEVYNLMCDLLK(L)	11.10	4.64	0.99	811.0558	4.00		
												2	14.43	14.43	70.8	1.70E+04	(K)NEVTSFENIEVYNLMCDLLK(L)	11.10	4.64	0.92	1216.0789	4.00		
5	5.1	15	6	101.96	72.4	1.44E+06	16251.0	4.65	*Daboia siamensis*	Q7T2R1	Acidic phospholipase A2 daboiatoxin A chain	2	21.59	21.59	90.6	1.47E+06	(R)CCFVHDCCYGTVNDCNPK(M)	5.17	0.00	0.95	1153.4348	5.21	4.99%	1.66%
												2	13.75	13.75	84.9	4.07E+06	(K)NYENYAISHCTEESEQC(-)	5.32	5.40	0.97	1067.4152	4.09		
												3	11.58	11.58	72.6	1.39E+06	(K)NYENYAISHCTEESEQC(-)	5.30	5.40	0.99	711.9453	4.09		
												3	17.82	11.97	84.2	5.72E+05	(R)AAAICLGQNVNTYDK(N)	5.97	0.00	0.99	546.6056	5.88		
												4	15.29	15.29	83.8	5.01E+06	(K)EAVHSYAIYGCYCGWGGQGKPQDATDR(C)	6.35	27.02	0.97	762.3397	5.38		
												3	26.20	26.20	100.0	1.70E+06	(K)EAVHSYAIYGCYCGWGGQGKPQDATDR(C)	6.35	21.62	1.00	1016.1160	5.38		
												2	20.52	20.52	87.5	7.43E+05	(R)AAAICLGQNVNTYDK(N)	8.20	151.82	0.93	819.4020	5.88		
												2	15.67	15.67	71.8	4.91E+06	(-)NFFQFAEMIVK(M)	10.25	10.81	0.93	687.3530	6.00		
												4	14.76	14.76	71.4	7.68E+05	(K)EAVHSYAIYGCYCGWGGQGKPQDATDR(C)	11.83	167.92	0.88	762.3375	5.38		
												2	14.59	14.59	71.0	9.21E+05	(R)AAAICLGQNVNTYDK(N)	11.93	130.92	0.94	819.4020	5.88		
												2	12.42	12.42	85.2	1.60E+04	(R)AAAICLGQNVNTYDK(N)	13.28	0.13	0.99	819.4020	5.88		
												2	15.75	15.75	79.4	1.67E+04	(-)NFFQFAEMIVK(M)	14.27	0.25	0.98	687.3505	6.00		
												3	19.19	19.19	83.1	1.41E+04	(K)MATYSYSFENGDIVCGDNNLCLK(T)	15.82	0.00	0.87	891.0545	4.03		
												4	12.06	12.06	74.4	6.68E+03	(K)EAVHSYAIYGCYCGWGGQGKPQDATDR(C)	14.92	0.00	0.69	762.3375	5.38		
												3	19.90	19.90	81.5	1.41E+04	(K)MATYSYSFENGDIVCGDNNLCLK(T)	15.82	0.00	0.87	891.0545	4.03		
5	5.2	10	4	79.90	43.4	1.42E+06	16441.3	4.93	*Daboia siamensis*	A8CG78	Acidic phospholipase A2 DsM-a2	2	17.97	17.97	85.2	2.61E+06	(K)YMLYSIFDCKEESDQC(-)	8.32	10.81	0.96	1044.4262	3.92	4.91%	1.64%
												3	10.84	10.84	73.7	8.52E+05	(K)YMLYSIFDCKEESDQC(-)	8.32	10.81	0.97	696.6195	3.92		
												2	18.53	18.53	70.3	1.38E+06	(K)YMLYSIFDCK(E)	8.48	10.81	1.00	670.3089	5.83		
												2	19.81	19.81	91.3	2.42E+06	(K)TGNFGLLSYVYYGCYCGWGGK(G)	9.65	21.61	0.99	1211.5375	8.05		
												3	22.76	22.76	86.9	2.43E+06	(K)TGNFGLLSYVYYGCYCGWGGK(G)	9.67	21.61	0.99	808.0284	8.05		
												3	20.64	20.64	88.9	5.00E+04	(K)TATYSYSFENGDIVCGGDDPCLR(A)	10.97	5.15	0.92	866.3701	3.84		
												3	19.69	19.69	83.9	4.27E+04	(K)TATYSYSFENGDIVCGGDDPCLR(A)	13.10	2.42	0.94	866.3701	3.84		
												3	17.37	17.37	80.3	4.36E+06	(K)TGNFGLLSYVYYGCYCGWGGK(G)	15.40	171.54	0.95	808.0260	8.05		
												3	17.17	17.17	77.2	1.24E+04	(K)TATYSYSFENGDIVCGGDDPCLR(A)	14.88	0.00	0.88	866.3701	3.84		
												3	17.86	17.86	77.1	8.39E+03	(K)TGNFGLLSYVYYGCYCGWGGK(G)	15.82	0.00	0.89	808.0260	8.05		
6	6.1	9	7	117.14	33.0	8.90E+05	29507.5	9.78	*Daboia siamensis*	P18965	Factor V activator RVV-V gamma	2	15.02	15.02	71.8	2.69E+05	(K)YFCLNTK(F)	5.62	0.00	1.00	473.2295	8.59	3.08%	1.03%
												2	16.12	10.63	90.7	1.49E+06	(R)TLCAGILK(G)	5.78	0.00	0.99	438.2565	8.41		
												2	13.06	13.06	78.8	7.24E+05	(R)EWVLTAAHCDR(R)	5.78	0.00	1.00	679.3214	5.32		
												3	13.82	13.82	81.3	4.03E+05	(R)RPVTYSTHIAPVSLPSR(S)	5.85	5.40	0.99	627.6773	10.84		
												3	16.75	16.75	85.0	4.51E+05	(K)FPNGLDKDIMLIR(L)	7.95	5.40	0.99	511.2833	5.96		
												2	18.84	18.84	90.9	3.87E+06	(K)WCEPLYPWVPADSR(T)	8.42	5.40	0.90	888.4184	4.37		
												3	20.99	16.05	95.7	6.96E+05	(K)WCEPLYPWVPADSR(T)	8.40	0.00	0.99	592.6135	4.37		
												2	21.38	21.38	93.4	6.36E+04	(K)VFDYNNWIQSIIAGNR(T)	9.85	0.00	0.96	955.4798	5.80		
												3	16.09	16.09	89.7	4.75E+04	(K)VFDYNNWIQSIIAGNR(T)	9.85	5.40	0.98	637.3244	5.80		
6	6.2	7	5	76.63	24.3	1.19E+06	29126.9	7.94	*Daboia russelii*	CL2958.contig6_DrSL	Serine protease VLSP-1	2	16.12	10.63	90.7	1.49E+06	(R)TLCAGILK(G)	5.78	0.00	0.99	438.2565	8.41	4.11%	1.37%
												2	14.70	14.70	77.9	7.06E+05	(R)FFCLSNK(N)	6.07	0.00	1.00	458.2252	8.75		
												2	16.65	16.65	83.6	1.91E+06	(R)HPCAQAELPAFYTK(V)	6.15	0.00	0.99	816.8994	6.75		
												3	15.47	15.47	84.1	1.48E+06	(R)HPCAQAELPAFYTK(V)	6.15	0.00	1.00	544.9351	6.75		
												3	23.35	13.60	97.9	1.12E+06	(R)LNSPVTYNTHIAPFSLPSSPPTVGSVCR(I)	8.05	10.94	0.90	1000.1770	8.75		
												3	19.57	13.31	87.0	1.04E+06	(K)SFTPWDKDIMLIR(L)	8.48	10.81	0.99	541.2869	5.68		
												2	20.51	20.51	86.2	5.56E+05	(K)SFTPWDKDIMLIR(L)	8.48	10.81	1.00	811.4265	5.68		
6	6.3	4	3	46.68	17.5	9.50E+05	28719.5	7.07	*Daboia siamensis*	E5L0E4	Beta-fibrinogenase-like	2	14.70	14.70	77.9	7.06E+05	(R)FFCLSNK(N)	6.07	0.00	1.00	458.2252	8.75	3.29%	1.10%
												2	17.83	12.51	97.0	2.06E+06	(K)TSTYIAPLSLPSSPPR(V)	7.32	0.00	1.00	843.9604	8.41		
												3	18.03	10.61	96.2	4.78E+05	(K)TSTYIAPLSLPSSPPR(V)	7.32	0.00	0.98	562.9749	8.41		
												3	13.95	7.01	77.6	5.57E+05	(K)VYDYTDWIQSIIAGNTAATCPP(-)	10.40	5.40	1.00	819.3889	3.56		
7	7.1	7	5	98.40	44.9	1.94E+06	16719.9	9.60	*Daboia russelii*	A8CG89	Basic phospholipase A2 Drk-b1	2	19.61	19.61	77.4	3.31E+06	(R)CCFVHDCCYAR(V)	4.83	5.40	0.97	774.2890	6.74	6.70%	2.23%
												2	21.48	13.69	92.3	3.05E+05	(R)VAAICLGQNVNTYNK(G)	6.15	0.00	0.86	832.9266	8.56		
												3	14.74	5.43	77.3	9.39E+05	(R)VAAICLGQNVNTYNK(G)	6.07	0.00	1.00	555.6218	8.56		
												2	25.82	25.82	97.5	4.10E+06	(K)YISYGCYCGWGGQGTPK(D)	6.53	5.40	0.90	977.4199	8.43		
												3	18.65	18.65	90.8	9.48E+05	(K)YISYGCYCGWGGQGTPK(D)	6.52	5.40	0.99	651.9489	8.43		
												2	17.88	14.19	79.3	3.74E+06	(K)GYMFLSSYYCR(Q)	7.80	5.40	0.95	723.8150	8.50		
												3	13.61	13.61	71.4	2.06E+05	(K)QEAFSFFKYISYGCYCGWGGQGTPK(D)	9.22	0.00	0.98	980.1064	8.38		
7	7.2	7	5	95.54	40.0	1.92E+06	15683.6	5.81	*Daboia siamensis*	Q7T3T5	Acidic phospholipase A2 daboiatoxin B chain	2	19.61	19.61	77.4	3.31E+06	(R)CCFVHDCCYAR(V)	4.83	5.40	0.97	774.2890	6.74	6.63%	2.21%
												2	25.82	25.82	97.5	4.10E+06	(K)YISYGCYCGWGGQGTPK(D)	6.53	5.40	0.90	977.4199	8.43		
												3	18.65	18.65	90.8	9.48E+05	(K)YISYGCYCGWGGQGTPK(D)	6.52	5.40	0.99	651.9489	8.43		
												2	17.97	17.97	85.2	2.61E+06	(K)YMLYSIFDCKEESDQC(-)	8.32	10.81	0.96	1044.4262	3.92		
												3	10.84	10.84	73.7	8.52E+05	(K)YMLYSIFDCKEESDQC(-)	8.32	10.81	0.97	696.6195	3.92		
												2	18.53	18.53	70.3	1.38E+06	(K)YMLYSIFDCK(E)	8.48	10.81	1.00	670.3089	5.83		
												3	13.61	13.61	71.4	2.06E+05	(K)QEAFSFFKYISYGCYCGWGGQGTPK(D)	9.22	0.00	0.98	980.1064	8.38		
8	8.1	6	6	109.48	13.5	2.11E+05	63610.7	9.15	*Daboia russelii*	CL3322.contig1_DrSL	Snake venom 5'-nucleotidase	3	16.07	11.47	83.2	8.23E+04	(K)VVYDLSQKPGSR(V)	4.82	5.40	0.99	450.2461	8.56	0.73%	0.24%
												2	15.03	15.03	73.5	2.00E+05	(K)LQNYYSQEIGK(T)	5.42	5.41	0.98	671.8366	6.00		
												3	15.61	9.87	86.1	3.14E+05	(K)VLLPSFLAAGGDGYHMLK(G)	8.77	0.00	0.98	630.3387	6.71		
												3	23.06	23.06	100.0	4.25E+05	(R)FHECNLGNLICDAVIYNNLR(H)	9.03	0.00	1.00	812.3930	5.32		
												3	19.04	11.48	91.5	1.50E+05	(K)YLGYLNVIFDDKGNVIK(A)	9.03	0.00	0.98	657.6929	5.96		
												2	20.67	20.67	88.9	9.28E+04	(K)YLGYLNVIFDDK(G)	9.32	0.00	0.98	730.3794	4.21		
8	8.2	3	3	58.01	6.9	1.08E+05	65308.5	9.01	*Crotalus adamanteus*	F8S0Z7	Snake venom 5'-nucleotidase	3	19.04	11.48	91.5	1.50E+05	(K)YLGYLNVIFDDKGNVIK(A)	9.03	0.00	0.98	657.6929	5.96	0.37%	0.12%
												2	20.67	20.67	88.9	9.28E+04	(K)YLGYLNVIFDDK(G)	9.32	0.00	0.98	730.3794	4.21		
												3	18.30	11.04	93.0	7.97E+04	(R)YDAMALGNHEFDNGLAGLLDPLLK(H)	10.40	0.00	0.87	863.1017	4.22		
9	9.1	7	6	109.41	43.3	6.75E+05	18523.6	6.31	*Daboia siamensis*	Q4PRC6	Snaclec 7	2	17.92	11.07	77.3	6.97E+05	(K)TTDNQWLR(W)	5.33	0.00	0.99	517.2589	5.50	2.34%	0.78%
												2	19.64	19.64	97.0	6.69E+05	(R)SSEEMDFVIR(M)	6.85	10.81	1.00	606.7842	4.14		
												3	16.80	16.80	79.0	3.84E+04	(K)FCNEQVNGGYLVSFR(S)	7.23	0.00	0.99	597.2839	6.00		
												2	13.14	13.14	75.6	1.52E+06	(R)MTFPIFR(F)	8.18	5.53	0.99	456.2459	9.50		
												2	24.00	24.00	92.7	4.38E+05	(K)QDCLSDWSFYEGYCYK(V)	8.13	5.53	0.99	1060.9239	4.03		

												3	11.02	11.02	84.8	1.10E+05	(K)QDCLSDWSFYEGYCYK(V)	8.13	5.53	0.99	707.6172	4.03		
												2	17.91	17.91	83.3	1.25E+06	(R)FDFFWIGLR(D)	10.45	5.40	0.98	600.8159	5.84		
9	9.2	9	4	80.52	50.6	2.53E+05	17529.7	6.58	*Daboia siamensis*	Q4PRC8	Snaclec 5	3	16.06	16.06	96.9	6.23E+05	(K)NHWSHMDCSSTHNFVCK(F)	4.88	0.00	0.97	716.2949	7.02	0.88%	0.29%
												3	14.12	7.98	89.5	6.04E+05	(K)GSHLLSLHNIAEADFVLK(K)	7.97	5.40	0.98	655.3604	5.99		
												2	16.60	10.20	95.2	2.64E+05	(K)GSHLLSLHNIAEADFVLK(K)	7.95	0.00	0.98	982.5364	5.99		
												2	24.00	24.00	92.7	4.38E+05	(K)QDCLSDWSFYEGYCYK(V)	8.13	5.53	0.99	1060.9239	4.03		
												3	11.02	11.02	84.8	1.10E+05	(K)QDCLSDWSFYEGYCYK(V)	8.13	5.53	0.99	707.6172	4.03		
												3	20.43	20.43	89.9	1.46E+05	(K)DGVIWMGLNDVWNECNWGWTDGAK(L)	10.42	16.22	0.96	941.7498	3.84		
												2	23.86	23.86	92.3	8.16E+04	(K)DGVIWMGLNDVWNECNWGWTDGAK(L)	10.38	21.62	0.96	1412.1183	3.84		
												4	12.65	12.65	85.3	7.89E+03	(K)GSHLLSLHNIAEADFVLK(K)	12.17	0.00	0.88	491.7710	5.99		
												4	10.08	10.08	79.0	5.56E+03	(K)GSHLLSLHNIAEADFVLK(K)	13.18	0.00	0.31	491.7710	5.99		
10	10.1	6	4	83.84	45.2	5.31E+05	17267.5	5.63	*Daboia siamensis*	Q4PRC9	Snaclec 4	2	24.04	24.04	95.6	9.84E+05	(K)VFTEEMNWADAEK(F)	6.93	5.41	0.98	785.3515	4.00	1.84%	0.61%
												2	18.92	18.92	85.1	3.16E+05	(K)SMTCNFIAPVVCK(F)	6.97	0.00	1.00	763.8650	8.47		
												3	21.01	12.35	94.3	1.07E+06	(K)ALAEESYCLIMITHEK(V)	7.60	5.40	0.99	636.6487	4.75		
												3	19.87	19.87	95.5	6.65E+05	(K)FVVNLISENLEYPATWIGLGNMWK(D)	11.45	9.81	1.00	932.1497	4.53		
												2	15.84	15.84	80.8	1.47E+05	(K)FVVNLISENLEYPATWIGLGNMWK(D)	11.45	9.81	0.98	1397.7156	4.53		
												4	10.60	10.60	70.9	5.82E+03	(K)FVVNLISENLEYPATWIGLGNMWK(D)	11.45	0.00	0.17	699.3677	4.53		
10	10.2	8	4	80.56	48.6	3.40E+05	17366.4	6.03	*Daboia siamensis*	Q4PRD0	Snaclec 3	3	16.06	16.06	96.9	6.23E+05	(K)NHWSHMDCSSTHNFVCK(F)	4.88	0.00	0.97	716.2949	7.02	1.18%	0.39%
												2	24.04	24.04	95.6	9.84E+05	(K)VFTEEMNWADAEK(F)	6.93	5.41	0.98	785.3515	4.00		
												3	14.12	7.98	89.5	6.04E+05	(K)GSHLLSLHNIAEADFVLK(K)	7.97	5.40	0.98	655.3604	5.99		
												2	16.60	10.20	95.2	2.64E+05	(K)GSHLLSLHNIAEADFVLK(K)	7.95	0.00	0.98	982.5364	5.99		
												3	20.43	20.43	89.9	1.46E+05	(K)DGVIWMGLNDVWNECNWGWTDGAK(L)	10.42	16.22	0.96	941.7498	3.84		
												2	23.86	23.86	92.3	8.16E+04	(K)DGVIWMGLNDVWNECNWGWTDGAK(L)	10.38	21.62	0.96	1412.1183	3.84		
												4	12.65	12.65	85.3	7.89E+03	(K)GSHLLSLHNIAEADFVLK(K)	12.17	0.00	0.88	491.7710	5.99		
												4	10.08	10.08	79.0	5.56E+03	(K)GSHLLSLHNIAEADFVLK(K)	13.18	0.00	0.31	491.7710	5.99		
11	11.1	5	3	63.11	66.6	3.64E+06	7191.8	9.69	*Daboia siamensis*	P00990	Kunitz-type serine protease inhibitor 2	4	15.30	15.30	86.4	4.17E+06	(-)HDRPTFCNLAPESGR(C)	5.10	5.40	0.99	439.9629	6.75	12.61%	4.20%
												2	17.46	12.19	98.3	3.14E+06	(-)HDRPTFCNLAPESGR(C)	5.08	5.40	1.00	878.9175	6.75		
												2	18.87	18.87	88.6	3.31E+06	(R)IYYNLESNK(C)	5.35	5.41	0.86	572.2914	6.00		
												2	26.78	26.78	92.5	5.51E+06	(K)VFFYGGCGGNANNFETR(D)	6.75	5.40	0.95	955.4208	5.97		
												3	20.05	20.05	85.9	2.08E+06	(K)VFFYGGCGGNANNFETR(D)	6.78	16.22	0.98	637.2840	5.97		
11	11.2	3	2	47.75	38.0	1.50E+06	9712.8	6.82	*Daboia siamensis*	A8Y7P4	Kunitz-type serine protease inhibitor B4	2	18.87	18.87	88.6	3.31E+06	(R)IYYNLESNK(C)	5.35	5.41	0.86	572.2914	6.00	5.19%	1.73%
												3	23.61	23.61	94.5	6.53E+05	(K)CEVFFYGGCGGNDNNFSTWDECR(H)	8.03	5.40	0.97	931.0275	3.91		
												2	28.88	28.88	97.9	5.30E+05	(K)CEVFFYGGCGGNDNNFSTWDECR(H)	8.03	5.40	0.98	1396.0363	3.91		
12	12.1	5	4	59.28	24.8	5.13E+05	29180.4	9.89	*Daboia siamensis*	E5L0E3	Alpha-fibrinogenase-like	3	18.37	18.37	91.6	2.04E+06	(R)VIYRPLPEQSR(T)	4.98	0.00	0.99	453.2601	8.72	1.78%	0.59%
												2	15.15	15.15	71.0	1.68E+05	(R)IMGWGSITSPK(K)	6.97	0.00	0.99	588.8105	8.75		
												3	10.52	1.25	74.2	7.15E+04	(K)ILPFVPHCANINIVPYTVCR(V)	8.58	5.40	0.99	795.0873	8.75		
												3	13.87	2.22	86.4	2.52E+05	(K)VFDYTDWIHSIIAGNTAATCPS(-)	10.02	5.40	1.00	813.7153	4.20		
												2	15.24	9.88	93.2	3.37E+04	(K)VFDYTDWIHSIIAGNTAATCPS(-)	9.95	0.00	0.98	1220.0685	4.20		
13	13.1	6	3	56.54	24.6	4.46E+05	17905.9	7.07	*Daboia siamensis*	Q38L02	Snaclec dabocetin subunit alpha	3	22.92	22.92	95.3	9.17E+05	(R)QQCSSHWTDGSAVSYETVTK(Y)	5.62	0.00	0.99	757.6740	5.32	1.54%	0.51%
												2	21.57	21.57	94.2	4.93E+05	(R)QQCSSHWTDGSAVSYETVTK(Y)	5.62	0.00	0.99	1136.0064	5.32		
												3	13.95	9.65	84.0	4.32E+05	(K)YHEWITLPCGDK(N)	6.68	0.00	1.00	506.9081	5.32		
												3	19.67	11.19	90.0	3.94E+05	(K)YHEWITLPCGDKNPFICK(S)	7.72	5.40	1.00	760.0335	6.75		
												4	11.46	5.29	71.3	3.99E+05	(K)YHEWITLPCGDKNPFICK(S)	7.72	5.40	0.99	570.2769	6.75		
												2	11.53	11.53	75.5	4.07E+04	(K)YHEWITLPCGDKNPFICK(S)	7.72	5.40	1.00	1139.5439	6.75		
14	14.1	5	3	55.04	7.8	2.52E+05	55887.4	5.44	*Daboia russelii*	CL3662.contig2_DrSL	Zinc metalloproteinase-disintegrin VLAIP-A	2	23.36	23.36	92.2	3.79E+05	(K)LTQGSQCADEECCDQCK(F)	3.97	3.58	0.96	1044.8894	3.91	0.87%	0.29%
												3	17.22	17.22	92.1	6.53E+05	(K)LTQGSQCADEECCDQCK(F)	3.97	3.58	0.94	696.9310	3.91		
												2	14.55	8.26	81.0	9.25E+04	(K)VTLDLFGK(W)	7.60	10.81	1.00	446.7616	5.81		
												2	17.13	10.67	81.2	1.13E+05	(R)IYEIVNILNVIYR(V)	10.65	5.40	0.97	811.4708	6.00		
												3	16.29	5.80	85.3	2.24E+04	(R)IYEIVNILNVIYR(V)	10.65	5.40	0.99	541.3157	6.00		
15	15.1	3	3	52.58	42.4	2.33E+05	12226.6	9.44	*Naja naja*	Unigene31385_Nn	Zinc metalloproteinase-disintegrin VLAIP-A	2	18.07	18.07	88.9	1.91E+05	(K)NPCQIYYAPSDENK(G)	5.27	5.40	1.00	849.8775	4.37	0.81%	0.27%
												2	12.82	12.82	73.9	4.01E+05	(K)QCISLFGSR(A)	6.60	0.00	0.99	534.2707	9.75		
												2	21.69	21.69	76.4	1.06E+05	(R)ATVAQDACFNFNSLGNAYGYCR(K)	7.90	10.81	0.97	1250.0456	5.88		
16	16.1	2	2	42.32	17.0	2.44E+06	18452.4	5.38	*Daboia siamensis*	K9JBV0	P68 alpha subunit	2	22.17	19.03	100.0	1.36E+06	(K)TPADYVWIGLR(N)	8.28	5.40	1.00	645.8473	5.50	8.45%	2.82%
												2	20.15	20.15	91.2	3.52E+06	(R)TWFNLSCGDDYPFVCK(F)	8.82	10.81	0.99	1004.9350	4.21		
17	17.1	2	2	41.55	21.3	5.40E+05	13625.1	9.69	*Daboia russelii*	P30894	Venom nerve growth factor	2	22.65	22.65	97.6	4.52E+05	(R)INTACVCVISR(K)	5.67	5.40	0.99	646.8275	9.75	1.87%	0.62%
												2	18.90	18.90	85.3	6.27E+05	(K)HWNSYCTTTDTFVR(A)	5.88	0.00	1.00	894.3791	6.74		
18	18.1	3	2	37.96	33.0	1.14E+06	13010.7	7.20	*Daboia siamensis*	P0DL42	Snake venom vascular endothelial growth factor toxin VR-1	2	12.85	12.85	72.0	3.63E+05	(K)HTADIQIMR(M)	4.98	0.00	1.00	542.7830	6.74	3.96%	1.32%
												3	25.11	20.20	98.4	2.77E+06	(R)ETLVSILQEHPDEISDIFRPSCVAVLR(C)	9.45	5.40	1.00	1041.8803	4.50		
												5	14.57	5.50	81.9	2.98E+05	(R)ETLVSILQEHPDEISDIFRPSCVAVLR(C)	9.43	5.40	0.98	625.5316	4.50		
19	19.1	2	2	36.09	16.4	1.15E+05	18793.1	5.84	*Daboia siamensis*	Q4PRD2	Snaclec coagulation factor X-activating enzyme light chain 2	2	20.91	16.32	90.8	1.71E+05	(-)LDCPPDSSLYR(Y)	5.50	10.81	0.99	661.8084	4.21	0.40%	0.13%
												3	15.18	15.18	75.1	5.95E+04	(K)FITHFWIGLMIK(D)	9.60	21.61	0.98	502.6172	8.76		
20	20.1	3	2	34.79	27.7	1.68E+06	10243.6	9.57	*Daboia siamensis*	A8Y7P5	Kunitz-type serine protease inhibitor B5	2	15.21	6.63	80.1	1.84E+06	(R)SFYYDSESK(K)	4.98	0.00	0.98	563.2429	4.37	5.81%	1.94%
												3	18.00	18.00	77.8	2.80E+06	(K)FCYLPADPGECLAHMR(S)	7.42	10.81	0.96	646.2904	5.32		
												2	19.58	19.58	86.0	3.92E+05	(K)FCYLPADPGECLAHMR(S)	7.42	10.81	0.99	968.9302	5.32		

21	21.1	3	2	32.36	14.1	1.85E+05	18116.4	9.16	*Macrovipera lebetina*	B4XSY7	Snaclec A12	3	15.88	15.88	86.4	5.37E+04	(K)LETHVWIGLR(V)	6.72	5.40	0.96	408.5690	6.75	0.64%	0.21%
												2	16.48	16.48	79.8	4.58E+05	(K)ANFVAELVTLMK(L)	10.72	5.40	1.00	668.3719	6.05		
												3	11.42	7.63	93.0	4.22E+04	(K)ANFVAELVTLMK(L)	10.72	5.40	0.99	445.9166	6.05		
22	22.1	2	2	32.29	5.5	4.68E+04	77077.2	5.92	*Boiga irregularis*	A0A0B8RNS9	Xaa-Pro aminopeptidase 2	2	12.81	12.81	75.7	2.16E+04	(R)GDDIPYTPVFYAYTLLTK(T)	10.83	5.41	0.95	1039.0300	4.21	0.16%	0.05%
												3	19.48	19.48	92.9	7.19E+04	(K)KPTAILLSGLEETAWLFNLR(G)	11.08	8.52	0.98	758.0961	6.14		
22	22.2	2	2	32.04	5.4	1.67E+04	74587.3	5.63	*Daboia russelii*	Unigene32033_DrSL	xaa-Pro aminopeptidase 2-like	2	12.81	12.81	75.7	2.16E+04	(R)GDDIPYTPVFYAYTLLTK(T)	10.83	5.41	0.95	1039.0300	4.21	0.06%	0.02%
												3	19.23	10.89	92.3	1.17E+04	(R)TLDLLEVNLVDLVWGSER(L)	11.52	0.00	0.67	691.0404	3.91		
23	23.1	2	1	27.76	6.9	9.04E+04	29577.7	9.77	*Macrovipera lebetina*	Q9PT40	Venom serine proteinase-like protein 2	2	27.76	27.76	96.9	1.11E+05	(R)FYCAGTLINQEWVLTAAR(C)	9.00	16.34	0.99	1057.0305	6.00	0.31%	0.10%
												3	19.22	19.22	86.7	6.97E+04	(R)FYCAGTLINQEWVLTAAR(C)	9.03	10.94	0.95	705.0236	6.00		
24	24.1	1	1	22.75	11.3	1.79E+05	18504.6	6.41	*Daboia siamensis*	K9JBU9	P31 alpha subunit	2	22.75	22.75	97.2	1.79E+05	(K)SVGEANFVAQLASGFMQK(D)	9.77	10.81	0.99	942.4709	5.72	0.62%	0.21%
25	25.1	1	1	22.58	5.8	4.69E+05	28981.6	7.11	*Macrovipera lebetina*	E0Y419	Beta-fibrinogenase	2	22.58	22.58	97.8	4.69E+05	(R)TLCAGILQGGIDTCK(G)	6.78	0.00	0.99	803.9033	5.50	1.62%	0.54%
26	26.1	2	1	20.83	6.6	3.43E+05	27826.7	5.61	*Gloydius blomhoffii*	Q8JI40	Cysteine-rich venom protein ablomin	4	12.40	3.14	94.7	3.50E+05	(R)KPEIQNEIVDLHNSLR(R)	6.52	5.40	0.98	477.0116	5.45	1.19%	0.40%
												3	20.83	14.66	96.6	3.35E+05	(R)KPEIQNEIVDLHNSLR(R)	6.52	5.40	0.98	635.6797	5.45		
						2.89E+07																	100.00%	33.33%

Daboia siamensis Guangxi venom protein (n=3) sorted according to protein families

Sample	Group	Subgroup	Spectra	Distinct Peptides	Distinct Summed MS/MS Search Score	% AA Coverage	Mean Protein Spectral Intensity	Protein MW (Da)	Protein pI	Species	Database Accession	Protein Name	Relative abundance (% overall)	Relative abundance of subtype (% overall)	Protein subtype
**Kunitz-type serine protease inhibitor (KSPI)**													**23.17%**	**23.17%**	**3**
C1	13	13.1	2	2	47.50	38.0	2.19E+06	9712.8	6.82	*Daboia siamensis*	A8Y7P4	Kunitz-type serine protease inhibitor B4	2.16%	6.53%	1
C2	9	9.2	5	3	61.62	39.2	2.20E+06	9712.8	6.82	*Daboia siamensis*	A8Y7P4	Kunitz-type serine protease inhibitor B4	2.64%		1
C3	11	11.2	3	2	47.75	38.0	1.50E+06	9712.8	6.82	*Daboia siamensis*	A8Y7P4	Kunitz-type serine protease inhibitor B4	1.73%		1
C1	22	22.1	3	2	34.74	27.7	1.44E+06	10243.6	9.57	*Daboia siamensis*	A8Y7P5	Kunitz-type serine protease inhibitor B5	1.42%	4.91%	2
C2	18	18.1	3	2	37.06	27.7	1.29E+06	10243.6	9.57	*Daboia siamensis*	A8Y7P5	Kunitz-type serine protease inhibitor B5	1.55%		2
C3	20	20.1	3	2	34.79	27.7	1.68E+06	10243.6	9.57	*Daboia siamensis*	A8Y7P5	Kunitz-type serine protease inhibitor B5	1.94%		2
C1	13	13.2	2	2	34.45	38.3	3.58E+06	7191.8	9.69	*Daboia siamensis*	P00990	Kunitz-type serine protease inhibitor 2	3.54%	11.72%	3
C2	9	9.1	6	4	70.59	68.3	3.31E+06	7191.8	9.69	*Daboia siamensis*	P00990	Kunitz-type serine protease inhibitor 2	3.98%		3
C3	11	11.1	5	3	63.11	66.6	3.64E+06	7191.8	9.69	*Daboia siamensis*	P00990	Kunitz-type serine protease inhibitor 2	4.20%		3
**Phospholipase A2 (PLA2)**													**22.18%**	**22.18%**	**6**
C1	7	7.2	8	3	62.65	43.4	1.38E+06	16441.3	4.93	*Daboia siamensis*	A8CG78	Acidic phospholipase A2 DsM-a2	1.37%	4.21%	1
C2	4	4.2	9	3	63.51	43.4	1.00E+06	16441.3	4.93	*Daboia siamensis*	A8CG78	Acidic phospholipase A2 DsM-a2	1.20%		1
C3	5	5.2	10	4	79.90	43.4	1.42E+06	16441.3	4.93	*Daboia siamensis*	A8CG78	Acidic phospholipase A2 DsM-a2	1.64%		1
C1	9	9.1	5	4	78.86	39.1	2.40E+06	16698.9	9.60	*Daboia siamensis*	A8CG82	Basic phospholipase A2 DsM-b1	2.38%	2.38%	2
C2	4	4.1	9	6	125.13	51.4	2.34E+06	16719.9	9.60	*Daboia russelii*	A8CG89	Basic phospholipase A2 Drk-b1	2.82%	5.05%	3
C3	7	7.1	7	5	98.40	44.9	1.94E+06	16719.9	9.60	*Daboia russelii*	A8CG89	Basic phospholipase A2 Drk-b1	2.23%		3
C1	7	7.1	10	5	83.13	55.7	2.40E+06	16251.0	4.65	*Daboia siamensis*	Q7T2R1	Acidic phospholipase A2 daboiatoxin A chain	2.38%	5.30%	4
C2	5	5.1	13	6	101.25	72.4	1.04E+06	16251.0	4.65	*Daboia siamensis*	Q7T2R1	Acidic phospholipase A2 daboiatoxin A chain	1.25%		4
C3	5	5.1	15	6	101.96	72.4	1.44E+06	16251.0	4.65	*Daboia siamensis*	Q7T2R1	Acidic phospholipase A2 daboiatoxin A chain	1.66%		4
C1	9	9.2	5	3	63.77	33.8	2.10E+06	15683.6	5.81	*Daboia siamensis*	Q7T3T5	Acidic phospholipase A2 daboiatoxin B chain	2.08%	4.29%	5
C3	7	7.2	7	5	95.54	40.0	1.92E+06	15683.6	5.81	*Daboia siamensis*	Q7T3T5	Acidic phospholipase A2 daboiatoxin B chain	2.21%		5
C2	5	5.2	13	5	98.97	68.1	7.96E+05	16256.9	4.61	*Daboia siamensis*	Q7ZZQ1	phospholipase A2-I	0.96%	0.96%	6
**Snake venom C-type lectin (Snaclec)**													**16.89%**	**16.89%**	**10**
C1	23	23.1	3	2	31.42	14.1	1.84E+05	18116.4	9.16	*Macrovipera lebetina*	B4XSY7	Snaclec A12	0.18%	0.64%	1
C2	20	20.2	3	2	31.37	14.1	2.06E+05	18116.4	9.16	*Macrovipera lebetina*	B4XSY7	Snaclec A12	0.25%		1
C3	21	21.1	3	2	32.36	14.1	1.85E+05	18116.4	9.16	*Macrovipera lebetina*	B4XSY7	Snaclec A12	0.21%		1
C1	25	25.1	1	1	23.47	11.3	1.31E+05	18504.6	6.41	*Daboia siamensis*	K9JBU9	P31 alpha subunit	0.13%	0.48%	2
C2	17	17.1	2	2	39.51	15.8	1.19E+05	18504.6	6.41	*Daboia siamensis*	K9JBU9	P31 alpha subunit	0.14%		2
C3	24	24.1	1	1	22.75	11.3	1.79E+05	18504.6	6.41	*Daboia siamensis*	K9JBU9	P31 alpha subunit	0.21%		2
C1	17	17.1	3	2	37.43	17.0	1.41E+06	18452.4	5.38	*Daboia siamensis*	K9JBV0	P68 alpha subunit	1.40%	5.86%	3
C2	14	14.1	3	2	43.62	17.0	1.37E+06	18452.4	5.38	*Daboia siamensis*	K9JBV0	P68 alpha subunit	1.65%		3
C3	16	16.1	2	2	42.32	17.0	2.44E+06	18452.4	5.38	*Daboia siamensis*	K9JBV0	P68 alpha subunit	2.82%		3
C1	12	12.1	4	3	53.06	24.6	5.06E+05	17905.9	7.07	*Daboia siamensis*	Q38L02	Snaclec dabocetin subunit alpha	0.50%	1.35%	4
C2	11	11.1	6	4	65.93	29.8	2.76E+05	17905.9	7.07	*Daboia siamensis*	Q38L02	Snaclec dabocetin subunit alpha	0.33%		4
C3	13	13.1	6	3	56.54	24.6	4.46E+05	17905.9	7.07	*Daboia siamensis*	Q38L02	Snaclec dabocetin subunit alpha	0.51%		4
C1	3	3.1	9	7	134.54	43.3	7.22E+05	18523.6	6.31	*Daboia siamensis*	Q4PRC6	Snaclec 7	0.72%	2.93%	5
C2	7	7.1	5	5	91.81	33.3	1.19E+06	18523.6	6.31	*Daboia siamensis*	Q4PRC6	Snaclec 7	1.43%		5
C3	9	9.1	7	6	109.41	43.3	6.75E+05	18523.6	6.31	*Daboia siamensis*	Q4PRC6	Snaclec 7	0.78%		5
C1	3	3.2	7	4	76.05	50.6	4.98E+05	17529.7	6.58	*Daboia siamensis*	Q4PRC8	Snaclec 5	0.49%	1.02%	6
C2	7	7.2	7	3	63.73	39.1	1.92E+05	17529.7	6.58	*Daboia siamensis*	Q4PRC8	Snaclec 5	0.23%		6
C3	9	9.2	9	4	80.52	50.6	2.53E+05	17529.7	6.58	*Daboia siamensis*	Q4PRC8	Snaclec 5	0.29%		6
C1	8	8.1	4	4	83.65	45.2	7.24E+05	17267.5	5.63	*Daboia siamensis*	Q4PRC9	Snaclec 4	0.72%	2.01%	7
C2	10	10.1	4	3	66.44	28.7	5.67E+05	17267.5	5.63	*Daboia siamensis*	Q4PRC9	Snaclec 4	0.68%		7
C3	10	10.1	6	4	83.84	45.2	5.31E+05	17267.5	5.63	*Daboia siamensis*	Q4PRC9	Snaclec 4	0.61%		7
C1	8	8.2	6	4	77.36	48.6	7.82E+05	17366.4	6.03	*Daboia siamensis*	Q4PRD0	Snaclec 3	0.77%	1.48%	8
C2	10	10.2	7	3	65.68	37.1	2.64E+05	17366.4	6.03	*Daboia siamensis*	Q4PRD0	Snaclec 3	0.32%		8
C3	10	10.2	8	4	80.56	48.6	3.40E+05	17366.4	6.03	*Daboia siamensis*	Q4PRD0	Snaclec 3	0.39%		8
C1	15	15.1	3	3	25.80	11.3	3.07E+05	18793.1	5.84	*Daboia siamensis*	Q4PRD2	Snaclec coagulation factor X-activating enzyme light chain 2	0.30%	0.84%	9
C2	13	13.1	3	3	44.44	16.4	3.35E+05	18793.1	5.84	*Daboia siamensis*	Q4PRD2	Snaclec coagulation factor X-activating enzyme light chain 2	0.40%		9
C3	19	19.1	2	2	36.09	16.4	1.15E+05	18793.1	5.84	*Daboia siamensis*	Q4PRD2	Snaclec coagulation factor X-activating enzyme light chain 2	0.13%		9
C2	20	20.1	2	2	33.24	15.7	2.30E+05	15889.7	9.56	*Daboia russelii*	Unigene30367_DrSL	C-type lectin A12	0.28%	0.28%	10
**Snake venom serine protease (SVSP)**													**13.61%**	**13.61%**	**9**
C1	26	26.1	1	1	21.59	5.8	4.79E+05	28981.6	7.11	*Macrovipera lebetina*	E0Y419	Beta-fibrinogenase	0.47%	1.57%	1
C2	24	24.1	1	1	23.11	5.8	4.63E+05	28981.6	7.11	*Macrovipera lebetina*	E0Y419	Beta-fibrinogenase	0.56%		1
C3	25	25.1	1	1	22.58	5.8	4.69E+05	28981.6	7.11	*Macrovipera lebetina*	E0Y419	Beta-fibrinogenase	0.54%		1
C1	14	14.1	3	3	46.12	16.2	1.75E+05	29180.4	9.89	*Daboia siamensis*	E5L0E3	Alpha-fibrinogenase-like	0.17%	0.77%	2
C3	12	12.1	5	4	59.28	24.8	5.13E+05	29180.4	9.89	*Daboia siamensis*	E5L0E3	Alpha-fibrinogenase-like	0.59%		2
C1	21	21.1	3	2	34.76	8.9	5.39E+05	28719.5	7.07	*Daboia siamensis*	E5L0E4	Beta-fibrinogenase-like	0.53%	2.50%	3
C2	6	6.3	4	3	51.13	17.5	7.27E+05	28719.5	7.07	*Daboia siamensis*	E5L0E4	Beta-fibrinogenase-like	0.87%		3
C3	6	6.3	4	3	46.68	17.5	9.50E+05	28719.5	7.07	*Daboia siamensis*	E5L0E4	Beta-fibrinogenase-like	1.10%		3
C1	5	5.1	9	7	122.17	33.0	7.94E+05	29507.5	9.78	*Daboia siamensis*	P18965	Factor V activator RVV-V gamma	0.79%	2.79%	4
C2	6	6.1	7	6	102.71	30.0	8.09E+05	29507.5	9.78	*Daboia siamensis*	P18965	Factor V activator RVV-V gamma	0.97%		4
C3	6	6.1	9	7	117.14	33.0	8.90E+05	29507.5	9.78	*Daboia siamensis*	P18965	Factor V activator RVV-V gamma	1.03%		4
C1	27	27.1	2	1	20.63	80.0	2.59E+05	1698.8	4.65	*Daboia russelii*	P86531	Vipera russelli proteinase RVV-V homolog 2	0.26%	0.59%	5
C2	23	23.1	2	1	24.05	80.0	2.77E+05	1698.8	4.65	*Daboia russelii*	P86531	Vipera russelli proteinase RVV-V homolog 2	0.33%		5
C1	24	24.1	2	1	26.84	6.9	1.05E+05	29577.7	9.77	*Macrovipera lebetina*	Q9PT40	Venom serine proteinase-like protein 2	0.10%	0.30%	6
C2	21	21.1	2	1	28.94	6.9	7.35E+04	29577.7	9.77	*Macrovipera lebetina*	Q9PT40	Venom serine proteinase-like protein 2	0.09%		6
C3	23	23.1	2	1	27.76	6.9	9.04E+04	29577.7	9.77	*Macrovipera lebetina*	Q9PT40	Venom serine proteinase-like protein 2	0.10%		6
C1	5	5.3	4	4	66.98	22.6	7.14E+05	28908.7	9.10	*Daboia russelii*	CL2958.contig11_DrSL	serine beta-fibrinogenase-like protein	0.71%	0.71%	7
C1	10	10.1	7	5	76.94	24.3	1.03E+06	29126.9	7.94	*Daboia russelii*	CL2958.contig6_DrSL	Serine protease VLSP-1	1.02%	3.25%	8
C2	6	6.2	7	5	72.73	24.3	7.16E+05	29126.9	7.94	*Daboia russelii*	CL2958.contig6_DrSL	Serine protease VLSP-1	0.86%		8
C3	6	6.2	7	5	76.63	24.3	1.19E+06	29126.9	7.94	*Daboia russelii*	CL2958.contig6_DrSL	Serine protease VLSP-1	1.37%		8
C1	5	5.2	6	4	76.29	21.5	1.15E+06	27523.2	9.03	*Naja naja*	CL31.contig2_Nn	RVV-V gamma-like protein	1.14%	1.14%	9
**Snake venom metalloproteinase (SVMP)**													**8.92%**	**8.92%**	**6**
C1	1	1.1	16	12	217.59	26.0	1.40E+06	71721.7	5.95	*Daboia russelii*	B8K1W0	Zinc metalloproteinase-disintegrin-like daborhagin-K	1.39%	4.44%	1

C2	1	1.1	17	10	185.33	22.6	1.70E+06	71721.7	5.95	*Daboia russelii*	B8K1W0	Zinc metalloproteinase-disintegrin-like daborhagin-K	2.04%		1
C3	1	1.1	20	12	205.40	26.0	8.69E+05	71721.7	5.95	*Daboia russelii*	B8K1W0	Zinc metalloproteinase-disintegrin-like daborhagin-K	1.00%		1
C1	6	6.1	9	6	113.88	11.9	2.17E+05	71801.6	6.01	*Daboia russelii*	K9JAW0	factor X activator heavy chain	0.21%	1.03%	2
C2	3	3.1	11	9	165.52	15.5	4.29E+05	71801.6	6.01	*Daboia russelii*	K9JAW0	factor X activator heavy chain	0.52%		2
C3	3	3.1	10	8	144.02	14.2	2.58E+05	71801.6	6.01	*Daboia russelii*	K9JAW0	factor X activator heavy chain	0.30%		2
C1	6	6.4	5	3	53.98	6.8	2.09E+05	70876.8	5.45	*Macrovipera lebetina*	Q4VM08	Zinc metalloproteinase-disintegrin-like VLAIP-A	0.21%	0.36%	3
C2	3	3.3	3	2	33.23	3.7	1.24E+05	70876.8	5.45	*Macrovipera lebetina*	Q4VM08	Zinc metalloproteinase-disintegrin-like VLAIP-A	0.15%		3
C1	11	11.1	5	3	59.00	10.0	3.06E+05	55887.4	5.44	*Daboia russelii*	CL3662.contig2_DrSL	Zinc metalloproteinase-disintegrin VLAIP-A	0.30%	1.02%	4
C2	16	16.1	4	2	42.03	6.1	3.56E+05	55887.4	5.44	*Daboia russelii*	CL3662.contig2_DrSL	Zinc metalloproteinase-disintegrin VLAIP-A	0.43%		4
C3	14	14.1	5	3	55.04	7.8	2.52E+05	55887.4	5.44	*Daboia russelii*	CL3662.contig2_DrSL	Zinc metalloproteinase-disintegrin VLAIP-A	0.29%		4
C1	6	6.3	6	4	69.00	51.8	3.38E+05	12226.6	9.44	*Naja naja*	Unigene31385_Nn	Zinc metalloproteinase-disintegrin VLAIP-A	0.33%	0.83%	5
C2	3	3.4	2	2	27.51	17.9	1.88E+05	12226.6	9.44	*Naja naja*	Unigene31385_Nn	Zinc metalloproteinase-disintegrin VLAIP-A	0.23%		5
C3	15	15.1	3	3	52.58	42.4	2.33E+05	12226.6	9.44	*Naja naja*	Unigene31385_Nn	Zinc metalloproteinase-disintegrin VLAIP-A	0.27%		5
C1	6	6.2	8	6	111.95	10.6	3.08E+05	71793.7	6.05	*Daboia russelii*	Unigene32626_DrSL	factor X activator heavy chain	0.30%	1.25%	6
C2	3	3.2	11	9	164.98	14.2	4.50E+05	71793.7	6.05	*Daboia russelii*	Unigene32626_DrSL	factor X activator heavy chain	0.54%		6
C3	3	3.2	9	8	142.36	12.9	3.46E+05	71793.7	6.05	*Daboia russelii*	Unigene32626_DrSL	factor X activator heavy chain	0.40%		6
**L-amino acid oxidase (LAAO)**													**5.95%**	**5.95%**	**4**
C1	2	2.2	13	10	173.61	29.1	4.32E+05	57286.2	8.82	*Daboia russelii*	G8XQX1	L-amino-acid oxidase	0.43%	1.29%	1
C2	2	2.1	15	11	184.18	26.3	3.52E+05	57286.2	8.82	*Daboia russelii*	G8XQX1	L-amino-acid oxidase	0.42%		1
C3	2	2.1	15	11	196.56	27.9	3.76E+05	57286.2	8.82	*Daboia russelii*	G8XQX1	L-amino-acid oxidase	0.43%		1
C1	2	2.3	4	2	31.01	48.8	7.00E+05	10351.4	5.10	*Vipera berus berus*	P0C2D7	L-amino-acid oxidase	0.69%	1.84%	2
C2	2	2.3	5	2	32.87	48.8	4.59E+05	10351.4	5.10	*Vipera berus berus*	P0C2D7	L-amino-acid oxidase	0.55%		2
C3	2	2.3	6	2	32.00	48.8	5.18E+05	10351.4	5.10	*Vipera berus berus*	P0C2D7	L-amino-acid oxidase	0.60%		2
C2	2	2.4	3	2	29.37	4.8	2.89E+05	58619.2	6.05	*Calloselasma rhodostoma*	P81382	L-amino-acid oxidase	0.35%	0.35%	3
C1	2	2.1	14	10	175.41	37.1	7.58E+05	46713.1	7.90	*Daboia siamensis*	Q4F867	L-amino-acid oxidase	0.75%	2.47%	4
C2	2	2.2	15	10	168.82	30.9	7.29E+05	46713.1	7.90	*Daboia siamensis*	Q4F867	L-amino-acid oxidase	0.88%		4
C3	2	2.2	14	10	182.92	32.9	7.28E+05	46713.1	7.90	*Daboia siamensis*	Q4F867	L-amino-acid oxidase	0.84%		4
**Snake venom vascular endothelial growth factor (svEGF)**													**4.79%**	**4.79%**	**1**
C1	18	18.1	3	2	37.17	33.0	1.57E+06	13010.7	7.20	*Daboia siamensis*	P0DL42	Snake venom vascular endothelial growth factor toxin VR-1	1.56%	4.79%	1
C2	22	22.1	2	1	25.36	24.7	1.59E+06	13010.7	7.20	*Daboia siamensis*	P0DL42	Snake venom vascular endothelial growth factor toxin VR-1	1.92%		1
C3	18	18.1	3	2	37.96	33.0	1.14E+06	13010.7	7.20	*Daboia siamensis*	P0DL42	Snake venom vascular endothelial growth factor toxin VR-1	1.32%		1
**Snake venom nerve growth factor (svNGF)**													**2.11%**	**2.11%**	**1**
C1	16	16.1	2	2	43.61	21.3	9.18E+05	13625.1	9.69	*Daboia russelii*	P30894	Venom nerve growth factor	0.91%	2.11%	1
C2	15	15.1	3	2	43.60	21.3	4.80E+05	13625.1	9.69	*Daboia russelii*	P30894	Venom nerve growth factor	0.58%		1
C3	17	17.1	2	2	41.55	21.3	5.40E+05	13625.1	9.69	*Daboia russelii*	P30894	Venom nerve growth factor	0.62%		1
**Snake venom 5'-nucleotidase (5' NUC)**													**0.82%**	**0.82%**	**3**
C1	20	20.1	2	2	35.11	6.9	3.23E+04	65308.5	9.01	*Crotalus adamanteus*	F8S0Z7	Snake venom 5'-nucleotidase	0.03%	0.16%	1
C3	8	8.2	3	3	58.01	6.9	1.08E+05	65308.5	9.01	*Crotalus adamanteus*	F8S0Z7	Snake venom 5'-nucleotidase	0.12%		1
C2	12	12.1	3	3	54.79	10.9	1.44E+05	55972.0	7.01	*Ovophis okinavensis*	U3T7C6	5'-nucleotidase	0.17%	0.17%	2
C1	20	20.2	2	2	32.02	4.7	7.36E+04	63610.7	9.15	*Daboia russelii*	CL3322.contig1_DrSL	Snake venom 5'-nucleotidase	0.07%	0.49%	3
C2	12	12.2	3	3	50.48	7.4	1.48E+05	63610.7	9.15	*Daboia russelii*	CL3322.contig1_DrSL	Snake venom 5'-nucleotidase	0.18%		3
C3	8	8.1	6	6	109.48	13.5	2.11E+05	63610.7	9.15	*Daboia russelii*	CL3322.contig1_DrSL	Snake venom 5'-nucleotidase	0.24%		3
**Cysteine-rich secretory protein (CRISP)**													**0.95%**	**0.95%**	**1**
C1	28	28.1	2	1	20.06	6.6	2.48E+05	27826.7	5.61	*Gloydius blomhoffii*	Q8JI40	Cysteine-rich venom protein ablomin	0.25%	0.95%	1
C2	25	25.1	2	1	21.67	6.6	2.61E+05	27826.7	5.61	*Gloydius blomhoffii*	Q8JI40	Cysteine-rich venom protein ablomin	0.31%		1
C3	26	26.1	2	1	20.83	6.6	3.43E+05	27826.7	5.61	*Gloydius blomhoffii*	Q8JI40	Cysteine-rich venom protein ablomin	0.40%		1
**Phosphodiesterase (PDE)**													**0.25%**	**0.25%**	**1**
C1	4	4.1	9	8	134.32	13.8	7.66E+04	98156.9	7.36	*Daboia russelii*	CL3655.contig2_DrSL	phosphodiesterase 1	0.08%	0.25%	1
C2	8	8.1	6	5	83.11	8.9	4.68E+04	98156.9	7.36	*Daboia russelii*	CL3655.contig2_DrSL	phosphodiesterase 1	0.06%		1
C3	4	4.1	9	8	140.33	15.7	1.00E+05	98156.9	7.36	*Daboia russelii*	CL3655.contig2_DrSL	phosphodiesterase 1	0.12%		1
**Aminopeptidase (non-toxin)**													**0.35%**	**0.35%**	**2**
C1	19	19.1	3	2	36.15	5.5	3.93E+04	77077.2	5.92	*Boiga irregularis*	A0A0B8RNS9	Xaa-Pro aminopeptidase 2	0.04%	0.20%	1
C2	19	19.1	2	2	34.00	5.5	9.11E+04	77077.2	5.92	*Boiga irregularis*	A0A0B8RNS9	Xaa-Pro aminopeptidase 2	0.11%		1
C3	22	22.1	2	2	32.29	5.5	4.68E+04	77077.2	5.92	*Boiga irregularis*	A0A0B8RNS9	Xaa-Pro aminopeptidase 2	0.05%		1
C1	19	19.2	3	2	32.86	5.4	2.74E+04	74587.3	5.63	*Daboia russelii*	Unigene32033_DrSL	xaa-Pro aminopeptidase 2-like	0.03%	0.15%	2
C2	19	19.2	2	2	32.21	5.4	8.79E+04	74587.3	5.63	*Daboia russelii*	Unigene32033_DrSL	xaa-Pro aminopeptidase 2-like	0.11%		2
C3	22	22.2	2	2	32.04	5.4	1.67E+04	74587.3	5.63	*Daboia russelii*	Unigene32033_DrSL	xaa-Pro aminopeptidase 2-like	0.02%		2

Daboia siamensis Guangxi venom summary table

Protein Name	Database Accession	Species	Relative abundance of subtype (% overall)	Protein subtype
**Kunitz-type serine protease inhibitor (KSPI)**			**23.17%**	**3**
Kunitz-type serine protease inhibitor B4	A8Y7P4	*Daboia siamensis*	6.53%	1
Kunitz-type serine protease inhibitor B5	A8Y7P5	*Daboia siamensis*	4.91%	2
Kunitz-type serine protease inhibitor 2	P00990	*Daboia siamensis*	11.72%	3
**Phospholipase A2 (PLA2)**			**22.18%**	**6**
**Neurotoxic**				
Acidic phospholipase A2 daboiatoxin A chain	Q7T2R1	*Daboia siamensis*	5.30%	1
Acidic phospholipase A2 daboiatoxin B chain	Q7T3T5	*Daboia siamensis*	4.29%	2
Basic phospholipase A2 DsM-b1	A8CG82	*Daboia siamensis*	2.38%	3
**Non-neurotoxic**				
Acidic phospholipase A2 DsM-a2	A8CG78	*Daboia siamensis*	4.21%	4
Basic phospholipase A2 Drk-b1	A8CG89	*Daboia russelii*	5.05%	5
phospholipase A2-I	Q7ZZQ1	*Daboia siamensis*	0.96%	6
**Snake venom C-type lectin (Snaclec)**			**16.89%**	**10**
Snaclec dabocetin subunit alpha	Q38L02	*Daboia siamensis*	1.35%	1
Snaclec A12	B4XSY7	*Macrovipera lebetina*	0.64%	2
C-type lectin A12	Unigene30367_DrSL	*Daboia russelii*	0.28%	3
Snaclec 7	Q4PRC6	*Daboia siamensis*	2.93%	4
Snaclec 5	Q4PRC8	*Daboia siamensis*	1.02%	5
Snaclec 4	Q4PRC9	*Daboia siamensis*	2.01%	6
Snaclec 3	Q4PRD0	*Daboia siamensis*	1.48%	7
P31 alpha subunit	K9JBU9	*Daboia siamensis*	0.48%	8
P68 alpha subunit	K9JBV0	*Daboia siamensis*	5.86%	9
Snaclec coagulation factor X-activating enzyme light chain 2	Q4PRD2	*Daboia siamensis*	0.84%	10
**Snake venom serine protease (SVSP)**			**13.61%**	**9**
Alpha-fibrinogenase-like	E5L0E3	*Daboia siamensis*	0.77%	1
Beta-fibrinogenase	E0Y419	*Macrovipera lebetina*	1.57%	2
Beta-fibrinogenase-like	E5L0E4	*Daboia siamensis*	2.50%	3
serine beta-fibrinogenase-like protein	CL2958.contig11_DrSL	*Daboia russelii*	0.71%	4
Factor V activator RVV-V gamma	P18965	*Daboia siamensis*	2.79%	5
Vipera russelli proteinase RVV-V homolog 2	P86531	*Daboia russelii*	0.59%	6
RVV-V gamma-like protein	CL31.contig2_Nn	*Naja naja*	1.14%	7
Venom serine proteinase-like protein 2	Q9PT40	*Macrovipera lebetina*	0.30%	8
Serine protease VLSP-1	CL2958.contig6_DrSL	*Daboia russelii*	3.25%	9
**Snake venom metalloproteinase (SVMP)**			**8.92%**	**6**
Zinc metalloproteinase-disintegrin-like daborhagin-K	B8K1W0	*Daboia russelii*	4.44%	1
Zinc metalloproteinase-disintegrin-like VLAIP-A	Q4VM08	*Macrovipera lebetina*	0.36%	2
Zinc metalloproteinase-disintegrin VLAIP-A	CL3662.contig2_DrSL	*Daboia russelii*	1.02%	3
Zinc metalloproteinase-disintegrin VLAIP-A	Unigene31385_Nn	*Naja naja*	0.83%	4
factor X activator heavy chain	K9JAW0	*Daboia russelii*	1.03%	5
factor X activator heavy chain	Unigene32626_DrSL	*Daboia russelii*	1.25%	6
**L-amino acid oxidase (LAAO)**			**5.95%**	**4**
L-amino-acid oxidase	G8XQX1	*Daboia russelii*	1.29%	1
L-amino-acid oxidase	P0C2D7	*Vipera berus berus*	1.84%	2
L-amino-acid oxidase	P81382	*Calloselasma rhodostoma*	0.35%	3
L-amino-acid oxidase	Q4F867	*Daboia siamensis*	2.47%	4
**Snake venom vascular endothelial growth factor (svEGF)**			**4.79%**	**1**
Snake venom vascular endothelial growth factor toxin VR-1	P0DL42	*Daboia siamensis*	4.79%	1
**Snake venom nerve growth factor (svNGF)**			**2.11%**	**1**
Venom nerve growth factor	P30894	*Daboia russelii*	2.11%	1
**Snake venom 5'-nucleotidase (5' NUC)**			**0.82%**	**3**
Snake venom 5'-nucleotidase	F8S0Z7	*Crotalus adamanteus*	0.16%	1
5'-nucleotidase	U3T7C6	*Ovophis okinavensis*	0.17%	2
Snake venom 5'-nucleotidase	CL3322.contig1_DrSL	*Daboia russelii*	0.49%	3
**Cysteine-rich secretory protein (CRISP)**			**0.95%**	**1**
Cysteine-rich venom protein ablomin	Q8JI40	*Gloydius blomhoffii*	0.95%	1
**Phosphodiesterase (PDE)**			**0.25%**	**1**
phosphodiesterase 1	CL3655.contig2_DrSL	*Daboia russelii*	0.25%	1
**Aminopeptidase (non-toxin)**			**0.35%**	**2**
Xaa-Pro aminopeptidase 2	A0A0B8RNS9	*Boiga irregularis*	0.20%	1
xaa-Pro aminopeptidase 2-like	Unigene32033_DrSL	*Daboia russelii*	0.15%	2
