# Supplementary material for: Venom proteomics and antivenom neutralization for the Chinese eastern Russell’s viper, Daboia siamensis from Guangxi and Taiwan
Source: Sci Rep. 2018 Jun 4;8:8545. doi: 10.1038/s41598-018-25955-y (PMC5986800; doi:10.1038/s41598-018-25955-y)
Supplement: Supplementary file 3 — Supplementary File S2B [file 41598_2018_25955_MOESM3_ESM.pdf]

Venom proteomics and antivenom neutralization for the Chinese Eastern Russell's viper, *Daboia siamensis* from Guangxi and Taiwan

[a]Kae Yi Tan, [b*]Choo Hock Tan, [a]Nget Hong Tan

[a]Department of Molecular Medicine, Faculty of Medicine, University of Malaya, Kuala Lumpur, Malaysia.

[b]Department of Pharmacology, Faculty of Medicine, University of Malaya, Kuala Lumpur, Malaysia.

Supplementary File S2B. LCMS-MS data collection for the Taiwan *Daboia siamensis* venom.

Daboia siamensis Taiwan venom 1

Group	Subgroup	Spectra	Distinct Peptides	Distinct Summed MS/MS Search Score	% AA Coverage	Mean Protein Spectral Intensity	Protein MW (Da)	Protein pI	Species	Database Accession	Protein Name	z	Score	Fwd-Rev Score	SPI (%)	Spectrum Intensity	Sequence	RT (min)	Peak Width (sec)	Averagine Chi Squared	m/z Measured (Da)	Peptide pI	Relative abundance (n=1)	Relative abundance (% overall)
1	1.1	22	15	266.26	25.6	6.84E+05	71928.8	5.98	*Daboia siamensis*	Q7LZ61	Coagulation factor X-activating enzyme heavy chain	4	17.77	17.77	89.0	1.95E+06	(R)ARDECDVPEHCTGQSAECPR(D)	4.20	0.00	0.97	594.2488	4.50	2.47%	0.82%
												3	24.20	24.20	100.0	6.80E+05	(R)ARDECDVPEHCTGQSAECPR(D)	4.20	0.00	0.99	791.9939	4.50		
												2	14.92	14.92	78.7	9.59E+04	(R)ARDECDVPEHCTGQSAECPR(D)	4.20	0.00	0.99	1187.4851	4.50		
												3	25.16	25.16	96.6	1.26E+06	(R)DECDVPEHCTGQSAECPR(D)	4.37	0.00	0.98	716.2823	4.17		
												2	26.08	26.08	96.8	4.25E+05	(R)DECDVPEHCTGQSAECPR(D)	4.37	0.00	0.99	1073.9171	4.17		
												3	14.62	14.62	76.4	8.70E+05	(R)KIPCAPQDVK(C)	4.63	5.40	1.00	385.8797	8.59		
												2	17.95	17.95	93.5	4.91E+05	(R)KIPCAPQDVK(C)	4.63	5.40	0.99	578.3148	8.59		
												2	15.19	15.19	79.4	1.26E+05	(K)GSYYGYCR(K)	4.68	5.40	1.00	513.2124	8.50		
												3	18.85	18.85	85.1	1.62E+06	(K)LKPGAECGNGLCCYQCK(I)	4.82	0.00	0.94	672.2951	8.50		
												2	13.36	13.36	72.1	1.19E+06	(K)DSCFQENLK(G)	5.02	5.40	0.99	570.7556	4.37		
												2	13.46	7.18	78.5	2.23E+05	(K)CIFNPPLRK(D)	5.90	5.40	1.00	572.8200	11.00		
												2	19.42	11.49	87.3	1.69E+06	(R)LFCLNNSPR(N)	6.08	0.00	0.98	560.7848	9.75		
												3	16.79	8.39	89.1	8.49E+04	(R)KSHDNALLFTDMR(F)	6.30	5.41	0.99	516.5946	6.75		
												5	10.20	10.20	73.0	1.23E+05	(K)TAVIMAHELSHNLGMYHDGK(N)	6.37	5.40	0.99	445.6208	6.18		
												4	11.59	11.59	81.9	1.45E+05	(K)TAVIMAHELSHNLGMYHDGK(N)	6.35	10.81	0.99	556.7745	6.18		
												2	16.09	16.09	74.8	2.04E+06	(R)NQCISLFGSR(A)	6.62	0.00	0.96	591.2936	9.75		
												2	13.55	13.55	81.8	1.11E+06	(K)CIFNPPLR(K)	6.72	0.00	0.99	508.7735	9.75		
												2	16.71	16.71	84.9	8.26E+04	(K)SHDNALLFTDMR(F)	6.73	5.40	0.93	710.3409	5.19		
												3	16.13	16.13	92.6	6.32E+04	(K)SHDNALLFTDMR(F)	6.72	0.00	0.99	473.8963	5.19		
												3	19.91	11.10	96.6	5.99E+04	(K)IFIELVIIVDHSMAK(K)	10.38	21.11	0.99	576.6648	5.32		
												3	20.84	17.82	83.9	4.61E+05	(R)FDLNTLGITFLAGMCQAYR(S)	10.92	3.37	1.00	731.0296	5.83		
												2	23.11	23.11	98.2	2.63E+05	(R)FDLNTLGITFLAGMCQAYR(S)	10.92	3.37	1.00	1096.0390	5.83		
1	1.2	6	5	77.37	9.0	3.02E+05	70876.8	5.45	*Macrovipera lebetina*	Q4VM08	Zinc metalloproteinase-disintegrin-like VLAIP-A	3	14.62	14.62	76.4	8.70E+05	(R)KIPCAPQDVK(C)	4.63	5.40	1.00	385.8797	8.59	1.09%	0.36%
												2	17.95	17.95	93.5	4.91E+05	(R)KIPCAPQDVK(C)	4.63	5.40	0.99	578.3148	8.59		
												2	15.97	15.97	81.8	2.76E+05	(K)NPCQIYYTPSDENK(G)	5.37	5.40	0.99	864.8843	4.37		
												2	19.26	19.26	84.2	3.68E+04	(R)LYCFDNLPEHK(N)	6.18	5.40	0.98	718.3371	5.32		
												2	12.98	5.61	85.3	1.34E+05	(K)VTLDLFGK(W)	7.80	0.00	1.00	446.7607	5.81		
												3	11.21	3.96	83.6	5.63E+03	(R)IYEIVNILNVIYR(V)	10.73	0.00	0.72	541.3160	6.00		
1	1.3	4	3	49.70	28.3	3.66E+05	12226.6	9.44	*Naja naja*	Unigene31385_Nn	Zinc metalloproteinase-disintegrin VLAIP-A	3	14.62	14.62	76.4	8.70E+05	(R)KIPCAPQDVK(C)	4.63	5.40	1.00	385.8797	8.59	1.32%	0.44%
												2	17.95	17.95	93.5	4.91E+05	(R)KIPCAPQDVK(C)	4.63	5.40	0.99	578.3148	8.59		
												2	19.26	19.26	84.2	3.68E+04	(R)LYCFDNLPEHK(N)	6.18	5.40	0.98	718.3371	5.32		
												2	12.49	12.49	73.6	6.49E+04	(K)QCISLFGSR(A)	6.62	10.81	0.92	534.2707	9.75		
1	1.4	4	3	47.35	7.8	3.65E+05	55887.4	5.44	*Daboia russelii*	CL3662.contig2_DrSL	Zinc metalloproteinase-disintegrin VLAIP-A	3	16.49	16.49	80.2	8.30E+05	(K)LTQGSQCADEECCDQCK(F)	3.92	4.09	0.90	696.9309	3.91	1.32%	0.44%
												2	23.16	23.16	95.5	4.90E+05	(K)LTQGSQCADEECCDQCK(F)	3.92	4.09	0.97	1044.8900	3.91		
												2	12.98	5.61	85.3	1.34E+05	(K)VTLDLFGK(W)	7.80	0.00	1.00	446.7607	5.81		
												3	11.21	3.96	83.6	5.63E+03	(R)IYEIVNILNVIYR(V)	10.73	0.00	0.72	541.3160	6.00		
2	2.1	13	12	209.80	22.0	7.28E+04	98156.9	7.36	*Daboia russelii*	CL3655.contig2_DrSL	phosphodiesterase 1	2	16.68	16.68	87.6	2.09E+05	(K)AATYFWPGSEVK(I)	7.38	10.81	1.00	678.3359	6.04	0.26%	0.09%
												2	17.53	17.53	95.0	1.54E+05	(K)DFYTFDSEGIVR(N)	7.78	5.40	0.99	724.8392	4.03		
												4	10.51	2.56	73.1	2.12E+05	(K)AERPDFITLYIEEPDTTGHK(F)	7.80	0.00	0.99	583.7931	4.50		
												3	11.68	11.68	70.8	8.90E+04	(R)LWNYFHGTLLPK(Y)	7.98	5.40	0.95	496.9396	8.60		
												2	16.65	16.65	73.9	4.71E+04	(K)VDFFMYEGPAPR(I)	8.07	0.00	1.00	714.8347	4.37		
												2	26.30	21.52	97.7	2.82E+04	(R)NPAWWGGQPIWHTVTYQGLK(A)	8.70	0.00	0.97	1170.0872	8.60		
												2	14.18	9.11	93.2	3.94E+04	(K)TFLPIFVNPVN(-)	9.72	5.40	0.99	630.8545	5.18		
												2	18.87	14.81	90.7	5.68E+03	(K)FGPVSGEIIMALQMADR(T)	9.78	0.00	0.05	917.9643	4.37		
												2	17.47	17.47	71.8	9.36E+03	(K)YISAYSQDILMPLWSSYTINK(S)	9.93	0.00	0.93	1247.1206	5.83		
												3	19.69	19.69	87.0	6.66E+04	(K)DQCASSSAAQCPAGFEQSPLILFSMDGFR(A)	10.37	0.00	0.97	1059.8080	4.03		
												3	20.77	20.77	94.9	3.71E+04	(K)GKNEVTSFENIEVYNLMCDLLK(L)	10.53	5.40	0.97	872.7607	4.41		
												3	19.47	10.78	99.9	3.55E+04	(K)NEVTSFENIEVYNLMCDLLK(L)	11.10	4.39	0.97	811.0576	4.00		
												2	16.18	9.31	78.0	1.35E+04	(K)NEVTSFENIEVYNLMCDLLK(L)	11.10	4.39	0.90	1216.0786	4.00		
3	3.1	10	8	135.70	42.3	1.73E+06	29507.5	9.78	*Daboia siamensis*	P18965	Factor V activator RVV-V gamma	3	14.54	14.54	81.2	7.23E+05	(R)NEDEQIRVPR(G)	4.82	0.00	1.00	419.2203	4.68	6.24%	2.08%
												2	16.32	10.65	89.6	1.88E+06	(R)TLCAGILK(G)	5.82	5.40	0.97	438.2572	8.41		
												2	12.72	12.72	75.8	8.53E+05	(R)EWVLTAAHCDR(R)	5.87	5.40	0.99	679.3220	5.32		
												3	17.67	17.67	95.2	1.06E+06	(R)RPVTYSTHIAPVSLPSR(S)	5.90	0.00	0.98	627.6860	10.84		
												3	20.50	8.62	94.4	5.91E+06	(K)ISTTEDTYPDVPHCTNIFIVK(H)	7.57	5.40	0.96	817.4067	4.54		
												2	16.93	11.27	93.0	6.81E+05	(K)ISTTEDTYPDVPHCTNIFIVK(H)	7.58	5.40	0.99	1225.6024	4.54		
												3	15.84	15.84	82.1	6.10E+05	(K)FPNGLDKDIMLIR(L)	7.98	0.00	1.00	511.2837	5.96		
												2	18.05	18.05	83.6	4.92E+06	(K)WCEPLYPWVPADSR(T)	8.45	16.21	1.00	888.3990	4.37		
												3	20.97	15.05	95.4	6.52E+05	(K)WCEPLYPWVPADSR(T)	8.47	16.21	0.99	592.6027	4.37		
												2	17.14	17.14	78.6	1.24E+04	(K)VFDYNNWIQSIIAGNR(T)	9.15	0.00	0.91	955.4815	5.80		
3	3.2	9	6	107.87	30.2	1.82E+06	27523.2	9.03	*Naja naja*	CL31.contig2_Nn	RVV-V gamma-like protein	2	16.32	10.65	89.6	1.88E+06	(R)TLCAGILK(G)	5.82	5.40	0.97	438.2572	8.41	6.58%	2.19%
												3	17.67	17.67	95.2	1.06E+06	(R)RPVTYSTHIAPVSLPSR(S)	5.90	0.00	0.98	627.6860	10.84		
												2	13.29	4.07	77.6	1.37E+05	(K)SFTPWDK(D)	6.18	5.40	0.98	440.7146	5.55		
												3	20.50	8.62	94.4	5.91E+06	(K)ISTTEDTYPDVPHCTNIFIVK(H)	7.57	5.40	0.96	817.4067	4.54		
												2	16.93	11.27	93.0	6.81E+05	(K)ISTTEDTYPDVPHCTNIFIVK(H)	7.58	5.40	0.99	1225.6024	4.54		
												2	18.05	18.05	83.6	4.92E+06	(K)WCEPLYPWVPADSR(T)	8.45	16.21	1.00	888.3990	4.37		
												3	20.97	15.05	95.4	6.52E+05	(K)WCEPLYPWVPADSR(T)	8.47	16.21	0.99	592.6027	4.37		
												3	19.12	14.94	80.9	7.95E+05	(K)SFTPWDKDIMLIR(L)	8.60	10.81	1.00	541.2846	5.68		
												2	18.87	14.92	75.6	3.65E+05	(K)SFTPWDKDIMLIR(L)	8.60	10.81	0.99	811.4205	5.68		
3	3.3	5	4	66.46	23.8	6.68E+05	28908.7	9.10	*Daboia russelii*	CL2958.contig11_DrSL	serine beta-fibrinogenase-like protein	3	14.54	14.54	81.2	7.23E+05	(R)NEDEQIRVPR(G)	4.82	0.00	1.00	419.2203	4.68	2.41%	0.80%
												2	12.72	12.72	75.8	8.53E+05	(R)EWVLTAAHCDR(R)	5.87	5.40	0.99	679.3220	5.32		
												3	15.84	15.84	82.1	6.10E+05	(K)FPNGLDKDIMLIR(L)	7.98	0.00	1.00	511.2837	5.96		
												3	23.36	13.57	98.0	9.92E+05	(R)LNSPVTYNTHIAPFSLPSSPPTVGSVCR(I)	8.07	0.00	0.99	1000.1764	8.75		
												4	15.75	10.55	87.5	1.63E+05	(R)LNSPVTYNTHIAPFSLPSSPPTVGSVCR(I)	8.07	5.40	0.94	750.3847	8.75		
4	4.1	9	7	120.42	30.8	8.66E+05	29126.9	7.94	*Daboia russelii*	CL2958.contig6_DrSL	Serine protease VLSP-1	2	14.49	14.49	90.6	9.13E+05	(R)AAYGGLPEK(S)	4.82	0.00	1.00	453.2428	6.04	3.13%	1.04%
												2	18.64	18.64	94.1	1.33E+06	(K)IYDYSVCR(A)	5.10	0.00	0.99	538.2498	5.83		
												2	16.32	10.65	89.6	1.88E+06	(R)TLCAGILK(G)	5.82	5.40	0.97	438.2572	8.41		
												2	13.29	4.07	77.6	1.37E+05	(K)SFTPWDK(D)	6.18	5.40	0.98	440.7146	5.55		
												3	15.20	15.20	83.7	1.22E+06	(R)HPCAQAELPAFYTK(V)	6.28	5.41	1.00	544.9362	6.75		
												3	23.36	13.57	98.0	9.92E+05	(R)LNSPVTYNTHIAPFSLPSSPPTVGSVCR(I)	8.07	0.00	0.99	1000.1764	8.75		
												4	15.75	10.55	87.5	1.63E+05	(R)LNSPVTYNTHIAPFSLPSSPPTVGSVCR(I)	8.07	5.40	0.94	750.3847	8.75		
												3	19.12	14.94	80.9	7.95E+05	(K)SFTPWDKDIMLIR(L)	8.60	10.81	1.00	541.2846	5.68		
												2	18.87	14.92	75.6	3.65E+05	(K)SFTPWDKDIMLIR(L)	8.60	10.81	0.99	811.4205	5.68		
5	5.1	22	5	105.42	67.3	3.50E+06	16276.8	4.51	*Daboia siamensis*	P31100	Acidic phospholipase A2 RV-7	2	22.59	22.59	90.3	3.62E+06	(R)CCFVHDCCYGTVNDCNPK(T)	5.23	5.40	0.89	1153.4356	5.21	12.64 %	4.21%
												2	12.59	12.59	84.7	3.71E+06	(K)NYEYYSISHCTEESEQC(-)	5.88	27.02	1.00	1099.9221	4.09		
												2	21.32	21.32	89.0	9.11E+06	(R)AAAICLGQNVNTYDK(N)	6.30	27.02	0.98	819.4036	5.88		
												3	17.62	17.62	84.2	2.27E+06	(R)AAAICLGQNVNTYDK(N)	6.12	27.02	1.00	546.6051	5.88		
												2	21.75	21.75	100.0	1.23E+06	(K)EVVHSYAIYGCYCGWGGQGR(A)	6.73	5.40	1.00	1160.0119	6.85		

												2	12.93	12.93	84.5	8.38E+05	(K)NYEYYSISHCTEESEQC(-)	7.62	145.90	1.00	1099.9189	4.09		
												2	18.41	18.41	81.4	1.43E+06	(R)AAAICLGQNVNTYDK(N)	8.15	113.47	0.95	819.4039	5.88		
												2	18.59	18.59	91.4	2.90E+05	(K)EVVHSYAIYGCYCGWGGQGR(A)	8.33	86.46	0.99	1160.0083	6.85		
												3	23.39	23.39	94.9	2.46E+06	(K)EVVHSYAIYGCYCGWGGQGR(A)	10.08	220.54	1.00	773.6760	6.85		
												2	24.84	24.84	94.7	7.08E+05	(K)TATYSYSFENGDIVCGDNDLCLR(T)	8.97	0.00	0.51	1335.5735	3.84		
												3	14.92	14.92	75.7	5.96E+03	(R)AAAICLGQNVNTYDK(N)	11.98	0.00	0.79	546.6042	5.88		
												2	20.28	20.28	84.6	4.41E+06	(R)AAAICLGQNVNTYDK(N)	12.02	196.23	0.94	819.4028	5.88		
												3	19.66	19.66	85.4	1.42E+07	(K)EVVHSYAIYGCYCGWGGQGR(A)	12.73	238.02	1.00	773.6749	6.85		
												2	25.10	25.10	94.9	2.72E+04	(K)TATYSYSFENGDIVCGDNDLCLR(T)	13.15	0.00	0.87	1335.5749	3.84		
												2	19.72	19.72	87.1	2.46E+06	(R)AAAICLGQNVNTYDK(N)	12.40	46.64	0.91	819.4028	5.88		
												3	24.15	24.15	94.3	6.22E+04	(K)TATYSYSFENGDIVCGDNDLCLR(T)	14.80	0.00	0.85	890.7210	3.84		
												2	22.64	22.64	92.9	2.72E+04	(K)TATYSYSFENGDIVCGDNDLCLR(T)	13.15	0.00	0.87	1335.5749	3.84		
												3	22.17	22.17	95.3	1.97E+07	(K)EVVHSYAIYGCYCGWGGQGR(A)	14.77	222.12	1.00	773.6749	6.85		
												2	21.41	21.41	85.9	1.80E+04	(R)AAAICLGQNVNTYDK(N)	14.13	0.13	0.79	819.4028	5.88		
												3	23.94	23.94	95.0	4.08E+04	(K)TATYSYSFENGDIVCGDNDLCLR(T)	16.25	0.00	0.85	890.7210	3.84		
												3	23.26	23.26	97.1	1.04E+07	(K)EVVHSYAIYGCYCGWGGQGR(A)	16.27	168.13	0.99	773.6749	6.85		
												3	23.58	23.58	93.6	5.17E+04	(K)TATYSYSFENGDIVCGDNDLCLR(T)	16.97	0.13	0.87	890.7210	3.84		
6	6.1	6	3	67.55	36.5	1.07E+06	14867.6	5.36	*Daboia siamensis*	Q4PRD1	Snaclec coagulation factor X-activating enzyme light chain 1	2	19.07	19.07	86.6	4.73E+05	(K)SMTCNFIAPVVCK(F)	6.93	10.81	1.00	763.8645	8.47	3.85%	1.28%
												3	13.58	13.58	75.9	1.35E+04	(K)SMTCNFIAPVVCK(F)	6.90	5.40	0.95	509.5785	8.47		
												3	21.68	21.68	84.4	2.85E+06	(-)VLDCPSGWLSYEQHCYK(G)	7.20	5.41	0.98	714.6554	5.32		
												2	22.26	22.26	89.3	6.70E+05	(-)VLDCPSGWLSYEQHCYK(G)	7.18	5.41	1.00	1071.4780	5.32		
												3	21.02	12.15	94.5	1.98E+06	(K)ALAEESYCLIMITHEK(E)	7.70	0.00	0.98	636.6487	4.75		
												2	26.22	22.57	100.0	4.12E+05	(K)ALAEESYCLIMITHEK(E)	7.70	0.00	1.00	954.4673	4.75		
6	6.2	3	2	34.57	20.9	4.80E+05	17366.4	6.03	*Daboia siamensis*	Q4PRD0	Snaclec 3	2	23.52	23.52	86.7	5.22E+05	(K)VFTEEMNWADAEK(F)	6.98	16.21	0.98	785.3510	4.00	1.73%	0.58%
												4	11.05	11.05	78.4	4.36E+05	(K)GSHLLSLHNIAEADFVLK(K)	7.98	5.40	0.99	491.7727	5.99		
												3	10.21	4.41	83.3	4.83E+05	(K)GSHLLSLHNIAEADFVLK(K)	7.98	5.40	0.99	655.3605	5.99		
7	7.1	4	3	64.03	46.6	8.26E+05	10503.8	9.75	*Daboia siamensis*	A8Y7N7	Kunitz-type serine protease inhibitor C4	2	14.95	5.90	79.6	1.67E+05	(R)SFYYDSESK(K)	4.92	5.41	0.99	563.2422	4.37	2.98%	0.99%
												3	24.19	17.41	88.5	1.83E+06	(K)EFIYGGCHGNANNFPTR(D)	5.80	10.81	0.97	651.9630	6.85		
												2	29.22	29.22	100.0	4.40E+05	(K)EFIYGGCHGNANNFPTR(D)	5.80	10.81	0.97	977.4406	6.85		
												2	19.86	19.86	85.7	8.68E+05	(K)FCYLPADPGECMAYIR(S)	7.98	0.00	0.98	981.9337	4.37		
8	8.1	8	3	57.91	32.6	5.22E+06	16410.4	9.99	*Daboia siamensis*	Q02471	Basic phospholipase A2 RV-4	2	12.51	12.51	75.5	3.97E+03	(R)TICECDR(V)	2.32	0.00	0.99	477.1949	4.37	18.82 %	6.27%
												2	20.45	20.45	77.7	4.11E+06	(R)CCFVHDCCYGGVK(G)	5.02	5.40	0.85	831.3244	6.74		
												3	24.95	24.95	94.8	4.32E+06	(K)LGAFSVWNYISYGCYCGWGGQGTPK(D)	10.13	79.10	0.98	943.4274	8.43		
												2	22.20	22.20	85.6	1.76E+06	(K)LGAFSVWNYISYGCYCGWGGQGTPK(D)	9.95	35.80	0.99	1414.6319	8.43		
												4	13.92	13.92	75.0	1.70E+04	(K)LGAFSVWNYISYGCYCGWGGQGTPK(D)	9.93	0.00	0.75	707.8229	8.43		
												3	23.48	23.48	91.3	1.04E+07	(K)LGAFSVWNYISYGCYCGWGGQGTPK(D)	11.97	117.23	0.98	943.4271	8.43		
												3	21.48	21.48	88.1	1.55E+07	(K)LGAFSVWNYISYGCYCGWGGQGTPK(D)	14.32	205.77	0.99	943.4256	8.43		
												3	17.59	17.59	73.7	5.61E+06	(K)LGAFSVWNYISYGCYCGWGGQGTPK(D)	15.58	113.66	0.88	943.4256	8.43		
9	9.1	4	3	55.76	21.5	1.49E+06	18452.4	5.38	*Daboia siamensis*	K9JBV0	P68 alpha subunit	2	12.66	12.66	71.6	3.01E+05	(K)NCFGLEK(Q)	5.00	0.00	0.99	434.2068	6.00	5.37%	1.79%
												2	22.17	22.17	100.0	2.11E+06	(K)TPADYVWIGLR(N)	8.25	0.00	0.96	645.8497	5.50		
												2	20.15	20.15	91.2	2.81E+06	(R)TWFNLSCGDDYPFVCK(F)	8.88	0.00	0.93	1004.9351	4.21		
												3	20.93	20.93	92.3	7.28E+05	(R)TWFNLSCGDDYPFVCK(F)	8.88	0.00	0.99	670.2925	4.21		
10	10.1	4	3	33.60	16.4	5.46E+05	18793.1	5.84	*Daboia siamensis*	Q4PRD2	Snaclec coagulation factor X-activating enzyme light chain 2	3	22.11	22.11	92.0	3.71E+05	(K)DKEQECSSEWSDGSSVSYDK(L)	5.27	32.42	0.99	774.9841	4.02	1.97%	0.66%
												2	18.86	18.86	78.8	1.94E+05	(K)DKEQECSSEWSDGSSVSYDK(L)	5.27	32.42	0.97	1161.9719	4.02		
												2	11.49	1.03	76.1	9.72E+05	(K)CFVLEK(E)	5.45	0.00	1.00	398.2097	6.00		
												2	19.90	15.78	94.0	6.46E+05	(-)LDCPPDSSLYR(Y)	5.57	5.40	1.00	661.8084	4.21		
11	11.1	3	2	47.10	38.0	1.01E+06	9712.8	6.82	*Daboia siamensis*	A8Y7P4	Kunitz-type serine protease inhibitor B4	2	17.60	17.60	86.3	7.73E+05	(R)IYYNLESNK(C)	5.47	5.40	1.00	572.2904	6.00	3.64%	1.21%
												3	24.37	24.37	94.8	1.17E+06	(K)CEVFFYGGCGGNDNNFSTWDECR(H)	8.05	10.81	0.89	931.0273	3.91		
												2	29.50	29.50	100.0	1.08E+06	(K)CEVFFYGGCGGNDNNFSTWDECR(H)	8.05	10.81	0.90	1396.0350	3.91		
11	11.2	3	2	36.60	38.3	1.25E+06	7191.8	9.69	*Daboia siamensis*	P00990	Kunitz-type serine protease inhibitor 2	3	19.00	7.30	92.8	2.09E+06	(-)HDRPTFCNLAPESGR(C)	5.18	0.00	1.00	586.2821	6.75	4.49%	1.50%
												4	15.33	15.33	86.5	8.72E+05	(-)HDRPTFCNLAPESGR(C)	5.18	0.00	0.99	439.9627	6.75		
												2	17.60	17.60	86.3	7.73E+05	(R)IYYNLESNK(C)	5.47	5.40	1.00	572.2904	6.00		
12	12.1	2	2	42.36	31.1	3.51E+06	10352.6	10.41	*Daboia siamensis*	A8Y7N4	Kunitz-type serine protease inhibitor C1	2	18.94	14.19	93.1	2.52E+06	(R)IYYNPDSNK(C)	4.63	5.40	0.97	557.2675	5.83	12.67 %	4.22%
												2	23.42	23.42	94.3	4.50E+06	(K)CEVFFYGGCGGNDNNFETR(K)	7.30	16.21	0.95	1121.9520	4.14		
13	13.1	3	2	36.42	33.0	1.25E+06	13010.7	7.20	*Daboia siamensis*	P0DL42	Snake venom vascular endothelial growth factor toxin VR-1	2	12.06	12.06	70.6	4.30E+05	(K)HTADIQIMR(M)	4.98	5.41	0.99	542.7844	6.74	4.51%	1.50%
												3	24.36	19.46	98.4	3.05E+06	(R)ETLVSILQEHPDEISDIFRPSCVAVLR(C)	9.43	10.81	1.00	1041.8804	4.50		
												5	14.05	4.75	80.8	2.67E+05	(R)ETLVSILQEHPDEISDIFRPSCVAVLR(C)	9.43	10.81	1.00	625.5311	4.50		
14	14.1	3	2	31.84	5.5	2.62E+04	77077.2	5.92	*Boiga irregularis*	A0A0B8RNS9	Xaa-Pro aminopeptidase 2	3	12.84	12.84	78.7	1.34E+04	(R)GDDIPYTPVFYAYTLLTK(T)	10.90	0.00	0.87	693.0236	4.21	0.09%	0.03%
												2	10.34	10.34	73.9	1.55E+04	(R)GDDIPYTPVFYAYTLLTK(T)	10.90	0.00	0.99	1039.0287	4.21		
												3	19.00	14.83	85.8	4.97E+04	(K)KPTAILLSGLEETAWLFNLR(G)	11.08	8.27	0.87	758.0967	6.14		
14	14.2	3	2	31.38	5.4	1.62E+04	74587.3	5.63	*Daboia russelii*	Unigene32033_DrSL	xaa-Pro aminopeptidase 2-like	3	12.84	12.84	78.7	1.34E+04	(R)GDDIPYTPVFYAYTLLTK(T)	10.90	0.00	0.87	693.0236	4.21	0.06%	0.02%
												2	10.34	10.34	73.9	1.55E+04	(R)GDDIPYTPVFYAYTLLTK(T)	10.90	0.00	0.99	1039.0287	4.21		
												3	18.54	18.54	92.1	1.96E+04	(R)TLDLLEVNLVDLVWGSER(L)	11.55	1.34	0.95	691.0414	3.91		
15	15.1	2	2	27.91	8.8	5.25E+05	18935.2	5.44	*Daboia siamensis*	K9JDJ1	factor X activator light chain 2	2	16.42	3.16	93.7	7.84E+04	(K)LLSNITEK(F)	5.02	5.40	0.99	459.2700	6.00	1.90%	0.63%
												2	11.49	1.03	76.1	9.72E+05	(K)CFVLEK(E)	5.45	0.00	1.00	398.2097	6.00		
16	16.1	3	2	26.51	12.7	1.33E+05	29180.4	9.89	*Daboia siamensis*	E5L0E3	Alpha-fibrinogenase-like	2	11.33	11.33	76.4	5.28E+04	(R)VIYRPLPEQSR(T)	4.92	5.41	0.98	679.3839	8.72	0.48%	0.16%
												3	15.18	5.03	85.9	1.82E+05	(K)VFDYTDWIHSIIAGNTAATCPS(-)	10.10	5.15	1.00	813.7158	4.20		
												2	13.08	7.26	81.0	1.63E+05	(K)VFDYTDWIHSIIAGNTAATCPS(-)	10.10	5.15	1.00	1220.0682	4.20		
						2.77E+07																	100.00 %	33.33%

Daboia siamensis Taiwan venom 2

Group	Subgroup	Spectra	Distinct Peptides	Distinct Summed MS/MS Search Score	% AA Coverage	Mean Protein Spectral Intensity	Protein MW (Da)	Protein pI	Species	Database Accession	Protein Name	z	Score	Fwd-Rev Score	SPI (%)	Spectrum Intensity	Sequence	RT (min)	Peak Width (sec)	Averagine Chi Squared	m/z Measured (Da)	Peptide pI	Relative abundance (n=1)	Relative abundance (% overall)
1	1.1	22	16	290.56	27.9	6.62E+05	71929	5.98	*Daboia siamensis*	Q7LZ61	Coagulation factor X-activating enzyme heavy chain	4	17.18	17.18	83.6	2.13E+06	(R)ARDECDVPEHCTGQSAECPR(D)	4.18	5.40	0.97	594.2479	4.50	2.67%	0.89%
												3	24.20	24.20	100.0	6.49E+05	(R)ARDECDVPEHCTGQSAECPR(D)	4.18	5.40	0.98	791.9935	4.50		
												2	14.74	14.74	77.8	8.23E+04	(R)ARDECDVPEHCTGQSAECPR(D)	4.18	5.40	1.00	1187.4833	4.50		
												3	24.39	24.39	96.2	1.24E+06	(R)DECDVPEHCTGQSAECPR(D)	4.38	0.00	0.98	716.2822	4.17		
												2	26.92	26.92	98.5	3.79E+05	(R)DECDVPEHCTGQSAECPR(D)	4.38	0.00	0.97	1073.9166	4.17		
												3	14.66	14.66	76.7	1.40E+06	(R)KIPCAPQDVK(C)	4.58	5.40	0.99	385.8798	8.59		
												2	18.45	18.45	93.5	7.81E+05	(R)KIPCAPQDVK(C)	4.58	5.40	1.00	578.3148	8.59		
												2	15.73	15.73	81.1	3.84E+05	(K)GSYYGYCR(K)	4.65	0.00	1.00	513.2115	8.50		
												2	19.25	19.25	94.8	7.41E+05	(K)IPCAPQDVK(C)	4.73	0.00	0.99	514.2678	5.84		
												2	19.07	11.87	96.5	4.00E+05	(R)SVEIVQEQGNR(N)	4.75	5.40	0.88	629.8255	4.53		
												3	16.11	16.11	83.3	1.10E+06	(K)LKPGAECGNGLCCYQCK(I)	4.85	5.40	0.85	672.2934	8.50		
												2	15.72	15.72	83.7	1.04E+05	(K)ASQLVSTSAQFNK(I)	5.42	5.40	0.98	690.8610	8.80		
												2	20.21	12.38	88.6	1.31E+06	(R)LFCLNNSPR(N)	6.05	5.40	1.00	560.7840	9.75		
												3	17.37	10.21	91.3	1.35E+05	(R)KSHDNALLFTDMR(F)	6.28	5.40	1.00	516.5948	6.75		
												5	13.04	13.04	78.1	2.45E+05	(K)TAVIMAHELSHNLGMYHDGK(N)	6.33	10.81	0.94	445.6196	6.18		

												4	11.50	11.50	79.5	2.05E+05	(K)TAVIMAHELSHNLGMYHDGK(N)	6.35	10.81	0.99	556.7719	6.18		
												2	16.72	16.72	76.4	1.84E+06	(R)NQCISLFGSR(A)	6.63	0.00	0.97	591.2935	9.75		
												2	13.57	13.57	82.4	8.10E+05	(K)CIFNPPLR(K)	6.72	10.81	1.00	508.7721	9.75		
												3	13.01	13.01	75.6	8.58E+04	(K)SHDNALLFTDMR(F)	6.78	5.40	0.90	473.8955	5.19		
												3	19.36	10.96	94.6	5.65E+04	(K)IFIELVIIVDHSMAK(K)	10.38	19.07	0.93	576.6648	5.32		
												3	21.83	18.01	87.4	3.30E+05	(R)FDLNTLGITFLAGMCQAYR(S)	10.92	4.96	1.00	731.0293	5.83		
												2	21.80	21.80	97.5	1.48E+05	(R)FDLNTLGITFLAGMCQAYR(S)	10.92	4.96	0.99	1096.0377	5.83		
1	1.2	8	6	102.45	9.0	4.35E+05	70877	5.45	*Macrovipera lebetina*	Q4VM08	Zinc metalloproteinase-disintegrin-like VLAIP-A	3	14.66	14.66	76.7	1.40E+06	(R)KIPCAPQDVK(C)	4.58	5.40	0.99	385.8798	8.59	1.76%	0.59%
												2	18.45	18.45	93.5	7.81E+05	(R)KIPCAPQDVK(C)	4.58	5.40	1.00	578.3148	8.59		
												2	19.25	19.25	94.8	7.41E+05	(K)IPCAPQDVK(C)	4.73	0.00	0.99	514.2678	5.84		
												2	15.91	15.91	77.1	2.62E+05	(K)NPCQIYYTPSDENK(G)	5.37	0.00	0.98	864.8826	4.37		
												2	19.31	19.31	83.2	1.22E+05	(R)LYCFDNLPEHK(N)	6.18	0.00	1.00	718.3401	5.32		
												2	13.64	6.52	80.2	1.50E+05	(K)VTLDLFGK(W)	7.80	0.00	1.00	446.7618	5.81		
												3	15.89	6.32	86.4	7.07E+03	(R)IYEIVNILNVIYR(V)	10.70	0.00	0.78	541.3166	6.00		
												2	15.58	8.13	80.7	1.83E+04	(R)IYEIVNILNVIYR(V)	10.75	0.00	0.99	811.4686	6.00		
1	1.3	5	3	53.22	7.8	4.36E+05	55887	5.44	*Daboia russelii*	CL3662.contig2_DrSL	Zinc metalloproteinase-disintegrin VLAIP-A	3	14.07	14.07	73.0	1.24E+06	(K)LTQGSQCADEECCDQCK(F)	3.92	2.62	0.94	696.9302	3.91	1.76%	0.59%
												2	23.69	23.69	92.2	7.67E+05	(K)LTQGSQCADEECCDQCK(F)	3.92	3.96	0.97	1044.8899	3.91		
												2	13.64	6.52	80.2	1.50E+05	(K)VTLDLFGK(W)	7.80	0.00	1.00	446.7618	5.81		
												3	15.89	6.32	86.4	7.07E+03	(R)IYEIVNILNVIYR(V)	10.70	0.00	0.78	541.3166	6.00		
												2	15.58	8.13	80.7	1.83E+04	(R)IYEIVNILNVIYR(V)	10.75	0.00	0.99	811.4686	6.00		
2	2.1	14	14	243.81	24.4	8.16E+04	98157	7.36	*Daboia russelii*	CL3655.contig2_DrSL	phosphodiesterase 1	2	14.23	14.23	81.9	1.16E+05	(R)MANVLCSCSEDCLEK(K)	6.18	0.00	0.90	908.3783	4.14	0.33%	0.11%
												2	18.04	18.04	90.6	2.20E+05	(K)AATYFWPGSEVK(I)	7.42	5.40	1.00	678.3375	6.04		
												2	17.57	12.62	86.1	1.43E+05	(K)DFYTFDSEGIVR(N)	7.78	5.40	0.97	724.8404	4.03		
												4	11.62	3.81	72.4	1.82E+05	(K)AERPDFITLYIEEPDTTGHK(F)	7.80	0.00	0.97	583.7943	4.50		
												2	13.42	6.76	80.9	4.50E+04	(R)TLGMLMEGLK(Q)	8.00	5.40	1.00	546.7936	5.66		
												3	12.64	12.64	71.2	4.69E+04	(R)LWNYFHGTLLPK(Y)	8.02	5.40	0.99	496.9381	8.60		
												2	19.21	19.21	90.6	5.12E+04	(K)VDFFMYEGPAPR(I)	8.07	0.00	1.00	714.8340	4.37		
												2	22.49	22.49	93.6	2.15E+04	(R)NPAWWGGQPIWHTVTYQGLK(A)	8.73	5.40	0.95	1170.0867	8.60		
												2	17.18	17.18	72.4	9.64E+04	(R)AGYLETWDSLMPNINK(L)	9.15	0.00	0.99	926.4519	4.37		
												2	11.49	6.29	81.4	6.78E+04	(K)TFLPIFVNPVN(-)	9.78	0.00	0.99	630.8538	5.18		
												2	20.50	20.50	88.5	1.34E+04	(K)FGPVSGEIIMALQMADR(T)	9.70	0.00	0.95	917.9641	4.37		
												3	21.44	21.44	88.5	5.78E+04	(K)DQCASSSAAQCPAGFEQSPLILFSMDGFR(A)	10.38	0.00	0.97	1059.8092	4.03		
												3	23.01	23.01	100.0	3.51E+04	(K)GKNEVTSFENIEVYNLMCDLLK(L)	10.53	0.00	0.97	872.7626	4.41		
												3	20.97	12.13	100.0	4.64E+04	(K)NEVTSFENIEVYNLMCDLLK(L)	11.12	6.56	1.00	811.0557	4.00		
3	3.1	8	7	124.24	38.4	2.21E+06	29508	9.78	*Daboia siamensis*	P18965	Factor V activator RVV-V gamma	2	16.37	10.63	90.7	1.53E+06	(R)TLCAGILK(G)	5.82	0.00	0.99	438.2566	8.41	8.93%	2.98%
												2	12.81	12.81	76.4	9.39E+05	(R)EWVLTAAHCDR(R)	5.85	5.40	1.00	679.3222	5.32		
												3	19.18	19.18	95.4	9.08E+05	(R)RPVTYSTHIAPVSLPSR(S)	5.92	0.00	0.99	627.6868	10.84		
												3	20.49	8.27	94.2	6.90E+06	(K)ISTTEDTYPDVPHCTNIFIVK(H)	7.58	10.81	0.95	817.4062	4.54		
												2	17.41	11.77	87.3	9.98E+05	(K)ISTTEDTYPDVPHCTNIFIVK(H)	7.58	5.40	0.99	1225.6014	4.54		
												3	16.00	16.00	84.3	7.27E+05	(K)FPNGLDKDIMLIR(L)	7.98	0.00	1.00	511.2839	5.96		
												2	16.91	16.91	79.0	5.68E+06	(K)WCEPLYPWVPADSR(T)	8.47	16.21	1.00	888.4001	4.37		
												2	22.48	22.48	89.5	3.14E+04	(K)VFDYNNWIQSIIAGNR(T)	9.73	10.81	0.90	955.4810	5.80		
3	3.2	4	3	50.61	20.0	6.41E+05	28909	9.10	*Daboia russelii*	CL2958.contig11_DrSL	serine beta-fibrinogenase-like protein	2	12.81	12.81	76.4	9.39E+05	(R)EWVLTAAHCDR(R)	5.85	5.40	1.00	679.3222	5.32	2.59%	0.86%
												3	16.00	16.00	84.3	7.27E+05	(K)FPNGLDKDIMLIR(L)	7.98	0.00	1.00	511.2839	5.96		
												3	21.80	12.11	96.8	7.70E+05	(R)LNSPVTYNTHIAPFSLPSSPPTVGSVCR(I)	8.07	0.00	0.99	1000.1751	8.75		
												4	17.09	17.09	82.9	1.28E+05	(R)LNSPVTYNTHIAPFSLPSSPPTVGSVCR(I)	8.08	5.40	0.92	750.3832	8.75		
4	4.1	22	5	103.80	67.3	2.58E+06	16277	4.51	*Daboia siamensis*	P31100	Acidic phospholipase A2 RV-7	2	21.59	21.59	90.3	4.24E+06	(R)CCFVHDCCYGTVNDCNPK(T)	5.23	16.21	0.85	1153.4363	5.21	10.43%	3.48%
												2	12.61	12.61	84.8	2.64E+06	(K)NYEYYSISHCTEESEQC(-)	5.82	10.81	0.98	1099.9221	4.09		
												3	11.02	11.02	74.6	1.85E+06	(K)NYEYYSISHCTEESEQC(-)	5.98	48.64	0.99	733.6173	4.09		
												3	17.62	17.62	84.2	1.87E+06	(R)AAAICLGQNVNTYDK(N)	6.12	27.02	0.99	546.6056	5.88		
												3	23.70	23.70	95.9	6.65E+06	(K)EVVHSYAIYGCYCGWGGQGR(A)	6.82	21.61	0.95	773.6783	6.85		
												2	23.00	23.00	100.0	1.52E+06	(K)EVVHSYAIYGCYCGWGGQGR(A)	6.83	27.02	1.00	1160.0109	6.85		
												2	14.83	14.83	70.2	1.10E+06	(R)AAAICLGQNVNTYDK(N)	8.12	113.48	0.97	819.4027	5.88		
												3	21.81	21.81	97.3	6.03E+05	(K)EVVHSYAIYGCYCGWGGQGR(A)	8.60	102.68	1.00	773.6746	6.85		
												3	13.43	13.43	76.4	1.11E+04	(R)AAAICLGQNVNTYDK(N)	11.48	0.13	0.80	546.6044	5.88		
												2	20.87	20.87	88.3	6.51E+06	(R)AAAICLGQNVNTYDK(N)	11.92	189.20	0.96	819.4027	5.88		
												3	23.38	23.38	94.7	7.39E+06	(K)EVVHSYAIYGCYCGWGGQGR(A)	11.95	144.43	1.00	773.6746	6.85		
												3	24.31	24.31	97.0	5.74E+04	(K)TATYSYSFENGDIVCGDNDLCLR(T)	13.98	0.00	0.89	890.7199	3.84		
												2	25.03	25.03	97.2	1.93E+04	(K)TATYSYSFENGDIVCGDNDLCLR(T)	13.85	0.00	0.87	1335.5750	3.84		
												2	17.44	17.44	81.2	1.09E+07	(R)AAAICLGQNVNTYDK(N)	13.13	211.96	0.86	819.4027	5.88		
												3	24.37	24.37	96.8	5.74E+04	(K)TATYSYSFENGDIVCGDNDLCLR(T)	13.98	0.00	0.89	890.7205	3.84		
												2	23.90	23.90	92.0	1.93E+04	(K)TATYSYSFENGDIVCGDNDLCLR(T)	13.85	0.00	0.87	1335.5750	3.84		
												3	20.14	20.14	88.4	1.13E+07	(K)EVVHSYAIYGCYCGWGGQGR(A)	14.87	210.12	0.99	773.6746	6.85		
												2	19.95	19.95	87.4	2.80E+04	(R)AAAICLGQNVNTYDK(N)	13.68	0.25	0.80	819.4027	5.88		
												3	23.88	23.88	94.1	4.56E+04	(K)TATYSYSFENGDIVCGDNDLCLR(T)	14.97	0.00	0.88	890.7205	3.84		
												3	21.80	21.80	72.2	2.56E+04	(K)EVVHSYAIYGCYCGWGGQGR(A)	15.37	0.25	0.95	773.6746	6.85		
												3	22.51	22.51	89.8	2.17E+04	(K)TATYSYSFENGDIVCGDNDLCLR(T)	17.20	0.00	0.88	890.7205	3.84		
												3	15.63	15.63	82.7	1.09E+04	(K)TATYSYSFENGDIVCGDNDLCLR(T)	21.32	0.00	0.86	890.7205	3.84		
5	5.1	6	5	82.01	25.0	7.30E+05	29127	7.94	*Daboia russelii*	CL2958.contig6_DrSL	Serine protease VLSP-1	2	18.64	18.64	94.2	8.04E+05	(K)IYDYSVCR(A)	5.07	5.40	1.00	538.2501	5.83	2.94%	0.98%
												2	16.37	10.63	90.7	1.53E+06	(R)TLCAGILK(G)	5.82	0.00	0.99	438.2566	8.41		
												2	13.33	4.04	78.7	1.47E+05	(K)SFTPWDK(D)	6.18	0.00	0.99	440.7152	5.55		
												3	11.87	11.87	75.1	1.00E+06	(R)HPCAQAELPAFYTK(V)	6.28	5.40	1.00	544.9350	6.75		
												3	21.80	12.11	96.8	7.70E+05	(R)LNSPVTYNTHIAPFSLPSSPPTVGSVCR(I)	8.07	0.00	0.99	1000.1751	8.75		
												4	17.09	17.09	82.9	1.28E+05	(R)LNSPVTYNTHIAPFSLPSSPPTVGSVCR(I)	8.08	5.40	0.92	750.3832	8.75		
6	6.1	9	4	69.34	39.8	2.75E+06	16410	9.99	*Daboia siamensis*	Q02471	Basic phospholipase A2 RV-4	2	11.93	4.06	77.2	3.99E+03	(R)TICECDR(V)	2.30	0.00	1.00	477.1947	4.37	11.09%	3.70%
												2	20.48	20.48	77.6	4.25E+06	(R)CCFVHDCCYGGVK(G)	4.98	5.40	0.86	831.3243	6.74		
												3	12.37	12.37	73.9	7.50E+04	(K)LAIYSYSFQR(G)	6.95	10.81	1.00	416.5536	8.59		
												3	23.68	23.68	86.0	2.97E+06	(K)LGAFSVWNYISYGCYCGWGGQGTPK(D)	10.05	66.57	0.98	943.4251	8.43		
												2	24.56	24.56	87.9	1.42E+06	(K)LGAFSVWNYISYGCYCGWGGQGTPK(D)	9.92	35.61	0.99	1414.6327	8.43		
												4	12.74	12.74	72.6	1.23E+04	(K)LGAFSVWNYISYGCYCGWGGQGTPK(D)	9.87	0.00	0.70	707.8216	8.43		
												3	22.91	22.91	87.3	8.59E+06	(K)LGAFSVWNYISYGCYCGWGGQGTPK(D)	11.90	115.67	0.98	943.4251	8.43		
												3	23.40	23.40	92.4	7.41E+06	(K)LGAFSVWNYISYGCYCGWGGQGTPK(D)	13.97	145.40	0.90	943.4251	8.43		
												3	18.01	18.01	79.1	7.55E+03	(K)LGAFSVWNYISYGCYCGWGGQGTPK(D)	14.90	0.00	0.62	943.4251	8.43		
7	7.1	5	3	65.90	36.5	1.20E+06	14868	5.36	*Daboia siamensis*	Q4PRD1	Snaclec coagulation factor X-activating enzyme light chain 1	2	18.10	18.10	86.5	4.37E+05	(K)SMTCNFIAPVVCK(F)	7.02	5.40	0.99	763.8649	8.47	4.83%	1.61%
												3	21.70	21.70	84.5	2.95E+06	(-)VLDCPSGWLSYEQHCYK(G)	7.18	10.81	0.99	714.6557	5.32		
												2	22.33	22.33	89.8	6.98E+05	(-)VLDCPSGWLSYEQHCYK(G)	7.18	10.81	1.00	1071.4777	5.32		
												3	22.46	12.39	93.0	1.64E+06	(K)ALAEESYCLIMITHEK(E)	7.72	5.40	0.99	636.6488	4.75		
												2	25.47	21.80	100.0	2.66E+05	(K)ALAEESYCLIMITHEK(E)	7.72	0.00	1.00	954.4678	4.75		
8	8.1	4	3	65.58	46.6	9.50E+05	10504	9.75	*Daboia siamensis*	A8Y7N7	Kunitz-type serine protease inhibitor C4	2	15.00	5.87	80.9	5.01E+05	(R)SFYYDSESK(K)	4.92	0.00	1.00	563.2433	4.37	3.83%	1.28%
												3	22.77	15.98	90.1	1.97E+06	(K)EFIYGGCHGNANNFPTR(D)	5.82	10.81	0.93	651.9627	6.85		
												2	29.22	29.22	100.0	4.61E+05	(K)EFIYGGCHGNANNFPTR(D)	5.82	10.81	0.94	977.4402	6.85		
												2	21.36	21.36	85.9	8.68E+05	(K)FCYLPADPGECMAYIR(S)	8.00	5.40	0.96	981.9328	4.37		
9	9.1	4	3	53.44	44.0	1.02E+06	13011	7.20	*Daboia siamensis*	P0DL42	Snake venom vascular endothelial growth factor toxin VR-1	3	18.60	18.60	70.7	5.54E+04	(K)FMEHTACECRPR(W)	4.15	5.40	0.99	531.8962	6.76	4.13%	1.38%
												2	11.23	11.23	72.2	5.85E+05	(K)HTADIQIMR(M)	4.92	0.00	0.99	542.7848	6.74		
												3	23.61	18.69	98.5	3.15E+06	(R)ETLVSILQEHPDEISDIFRPSCVAVLR(C)	9.45	5.40	1.00	1041.8815	4.50		
												5	13.88	4.57	82.0	3.04E+05	(R)ETLVSILQEHPDEISDIFRPSCVAVLR(C)	9.45	5.40	1.00	625.5317	4.50		
10	10.1	4	3	33.59	16.4	5.66E+05	18793	5.84	*Daboia siamensis*	Q4PRD2	Snaclec coagulation factor X-activating enzyme light chain 2	3	21.23	21.23	83.1	3.70E+05	(K)DKEQECSSEWSDGSSVSYDK(L)	5.28	32.43	0.98	774.9855	4.02	2.28%	0.76%
												2	22.06	22.06	90.3	1.70E+05	(K)DKEQECSSEWSDGSSVSYDK(L)	5.30	32.43	0.97	1161.9720	4.02		

												2	11.53	1.05	77.1	8.09E+05	(K)CFVLEK(E)	5.47	0.00	1.00	398.2087	6.00		
												2	19.45	15.36	94.9	9.15E+05	(-)LDCPPDSSLYR(Y)	5.57	5.40	1.00	661.8090	4.21		
11	11.1	3	2	49.01	38.0	9.30E+05	9712.8	6.82	*Daboia siamensis*	A8Y7P4	Kunitz-type serine protease inhibitor B4	2	19.51	19.51	92.5	1.01E+06	(R)IYYNLESNK(C)	5.47	0.00	1.00	572.2897	6.00	3.75%	1.25%
												2	29.50	29.50	100.0	8.96E+05	(K)CEVFFYGGCGGNDNNFSTWDECR(H)	8.05	10.81	0.91	1396.0343	3.91		
												3	23.23	23.23	95.1	8.85E+05	(K)CEVFFYGGCGGNDNNFSTWDECR(H)	8.05	10.81	0.90	931.0264	3.91		
11	11.2	4	2	38.56	38.3	2.52E+06	7191.8	9.69	*Daboia siamensis*	P00990	Kunitz-type serine protease inhibitor 2	3	19.05	6.96	94.3	4.98E+06	(-)HDRPTFCNLAPESGR(C)	5.13	5.40	0.97	586.2825	6.75	10.15%	3.38%
												4	15.25	15.25	86.2	2.47E+06	(-)HDRPTFCNLAPESGR(C)	5.12	5.40	1.00	439.9631	6.75		
												2	19.03	13.86	100.0	1.61E+06	(-)HDRPTFCNLAPESGR(C)	5.12	5.40	0.99	878.9179	6.75		
												2	19.51	19.51	92.5	1.01E+06	(R)IYYNLESNK(C)	5.47	0.00	1.00	572.2897	6.00		
12	12.1	3	2	43.22	17.0	2.06E+06	18452	5.38	*Daboia siamensis*	K9JBV0	P68 alpha subunit	2	22.17	18.68	100.0	2.45E+06	(K)TPADYVWIGLR(N)	8.25	0.00	0.92	645.8493	5.50	8.30%	2.77%
												2	20.14	20.14	91.1	2.92E+06	(R)TWFNLSCGDDYPFVCK(F)	8.90	5.40	0.97	1004.9352	4.21		
												3	21.05	21.05	94.2	8.00E+05	(R)TWFNLSCGDDYPFVCK(F)	8.90	5.40	1.00	670.2923	4.21		
13	13.1	3	2	41.06	31.1	3.01E+06	10353	10.41	*Daboia siamensis*	A8Y7N4	Kunitz-type serine protease inhibitor C1	2	17.64	12.83	91.2	3.19E+06	(R)IYYNPDSNK(C)	4.60	5.40	0.99	557.2667	5.83	12.14%	4.05%
												2	23.42	23.42	94.3	4.39E+06	(K)CEVFFYGGCGGNDNNFETR(K)	7.28	16.21	0.95	1121.9529	4.14		
												3	17.64	17.64	84.5	1.45E+06	(K)CEVFFYGGCGGNDNNFETR(K)	7.30	16.21	0.92	748.3051	4.14		
14	14.1	2	2	37.04	5.5	3.61E+04	77077	5.92	*Boiga irregularis*	A0A0B8RNS 9	Xaa-Pro aminopeptidase 2	3	16.64	11.20	83.4	7.70E+03	(R)GDDIPYTPVFYAYTLLTK(T)	10.88	0.00	0.54	693.0245	4.21	0.15%	0.05%
												3	20.40	20.40	88.7	6.45E+04	(K)KPTAILLSGLEETAWLFNLR(G)	11.10	9.74	0.93	758.0955	6.14		
15	15.1	2	2	25.71	8.8	4.81E+05	18935	5.44	*Daboia siamensis*	K9JDJ1	factor X activator light chain 2	2	14.18	2.42	94.3	1.52E+05	(K)LLSNITEK(F)	5.02	0.00	1.00	459.2698	6.00	1.94%	0.65%
												2	11.53	1.05	77.1	8.09E+05	(K)CFVLEK(E)	5.47	0.00	1.00	398.2087	6.00		
16	16.1	3	2	25.08	12.7	1.40E+05	29180	9.89	*Daboia siamensis*	E5L0E3	Alpha-fibrinogenase-like	2	10.52	10.52	76.1	1.12E+05	(R)VIYRPLPEQSR(T)	4.92	0.00	0.98	679.3861	8.72	0.56%	0.19%
												3	14.25	2.95	88.2	1.67E+05	(K)VFDYTDWIHSIIAGNTAATCPS(-)	10.10	4.39	0.99	813.7153	4.20		
												2	14.56	9.59	87.2	1.41E+05	(K)VFDYTDWIHSIIAGNTAATCPS(-)	10.10	4.39	1.00	1220.0663	4.20		
17	17.1	1	1	22.12	5.8	2.86E+05	28982	7.11	*Macrovipera lebetina*	E0Y419	Beta-fibrinogenase	2	22.12	22.12	94.4	2.86E+05	(R)TLCAGILQGGIDTCK(G)	6.83	5.40	0.99	803.9029	5.50	1.15%	0.38%
18	18.1	3	2	22.12	12.8	3.33E+05	17366	6.03	*Daboia siamensis*	Q4PRD0	Snaclec 3	5	11.09	11.09	91.4	1.22E+05	(K)GSHLLSLHNIAEADFVLKK(T)	7.43	0.00	1.00	419.2394	6.92	1.34%	0.45%
												4	11.03	11.03	79.4	4.40E+05	(K)GSHLLSLHNIAEADFVLK(K)	8.00	5.40	0.98	491.7727	5.99		
												3	10.46	10.46	75.5	4.38E+05	(K)GSHLLSLHNIAEADFVLK(K)	8.00	5.40	0.98	655.3601	5.99		
19	19.1	2	1	21.02	11.9	7.32E+05	13625	9.69	*Daboia russelii*	P30894	Venom nerve growth factor	2	21.02	14.99	86.7	7.80E+05	(K)HWNSYCTTTDTFVR(A)	5.92	0.00	1.00	894.3988	6.74	2.95%	0.98%
												3	16.98	16.98	70.9	6.83E+05	(K)HWNSYCTTTDTFVR(A)	5.92	0.00	0.99	596.6017	6.74		
						2.48E+07																	100.00%	33.33%

Daboia siamensis Taiwan venom 3

Group	Subgroup	Spectra	Distinct Peptides	Distinct Summed MS/MS Search Score	% AA Coverage	Mean Protein Spectral Intensity	Protein MW (Da)	Protein pI	Species	Database Accession	Protein Name	z	Score	Fwd-Rev Score	SPI (%)	Spectrum Intensity	Sequence	RT (min)	Peak Width (sec)	Averagine Chi Squared	m/z Measured (Da)	Peptide pI	Relative abundance (n=1)	Relative abundance (% overall)
1	1.1	24	16	294.74	25.3	5.98E+05	71928.8	5.98	*Daboia siamensis*	Q7LZ61	Coagulation factor X-activating enzyme heavy chain	4	15.65	15.65	77.3	1.87E+06	(R)ARDECDVPEHCTGQSAECPR(D)	4.18	0.00	0.97	594.2483	4.50	2.21%	0.74%
												3	24.20	24.20	100.0	5.92E+05	(R)ARDECDVPEHCTGQSAECPR(D)	4.18	0.00	0.98	791.9935	4.50		
												2	13.04	13.04	72.8	7.40E+04	(R)ARDECDVPEHCTGQSAECPR(D)	4.18	0.00	0.99	1187.4845	4.50		
												3	25.12	25.12	96.2	1.34E+06	(R)DECDVPEHCTGQSAECPR(D)	4.37	0.00	0.98	716.2817	4.17		
												2	27.91	27.91	98.3	4.31E+05	(R)DECDVPEHCTGQSAECPR(D)	4.37	0.00	0.97	1073.9167	4.17		
												3	14.79	14.79	78.2	2.92E+05	(R)KIPCAPQDVK(C)	4.62	5.40	0.99	385.8792	8.59		
												2	18.45	18.45	93.4	1.56E+05	(R)KIPCAPQDVK(C)	4.62	5.40	1.00	578.3145	8.59		
												2	15.30	15.30	81.8	5.08E+05	(K)GSYYGYCR(K)	4.63	0.00	1.00	513.2125	8.50		
												2	19.25	19.25	94.9	1.43E+06	(K)IPCAPQDVK(C)	4.73	0.00	0.99	514.2683	5.84		
												2	19.11	11.89	97.3	1.36E+06	(R)SVEIVQEQGNR(N)	4.73	0.00	1.00	629.8256	4.53		
												2	19.99	19.99	90.1	1.33E+05	(K)ASQLVSTSAQFNK(I)	5.38	5.40	0.98	690.8619	8.80		
												2	13.50	7.32	79.2	2.22E+05	(K)CIFNPPLRK(D)	5.85	10.81	1.00	572.8198	11.00		
												2	19.42	11.57	87.5	1.98E+06	(R)LFCLNNSPR(N)	6.02	5.40	0.99	560.7852	9.75		
												3	16.28	9.63	88.2	1.06E+05	(R)KSHDNALLFTDMR(F)	6.27	0.00	0.99	516.5942	6.75		
												4	12.11	12.11	82.0	2.74E+05	(K)TAVIMAHELSHNLGMYHDGK(N)	6.33	10.94	0.99	556.7737	6.18		
												5	10.01	10.01	80.0	3.22E+05	(K)TAVIMAHELSHNLGMYHDGK(N)	6.32	16.34	0.91	445.6209	6.18		
												2	16.24	16.24	76.7	1.33E+06	(R)NQCISLFGSR(A)	6.62	5.40	1.00	591.2928	9.75		
												2	13.56	13.56	82.1	7.05E+05	(K)CIFNPPLR(K)	6.68	10.81	0.99	508.7726	9.75		
												2	16.07	16.07	83.4	2.13E+05	(K)SHDNALLFTDMR(F)	6.72	0.00	0.99	710.3414	5.19		
												3	15.17	15.17	93.1	1.91E+05	(K)SHDNALLFTDMR(F)	6.72	0.00	0.99	473.8965	5.19		
												3	18.70	10.02	98.0	2.16E+05	(K)IFIELVIIVDHSMAK(K)	10.40	31.41	1.00	576.6647	5.32		
												2	20.13	13.85	89.0	2.69E+04	(K)IFIELVIIVDHSMAK(K)	10.37	10.04	0.75	864.4926	5.32		
												3	22.05	18.12	87.7	3.58E+05	(R)FDLNTLGITFLAGMCQAYR(S)	10.90	4.39	1.00	731.0297	5.83		
												2	23.22	23.22	100.0	2.17E+05	(R)FDLNTLGITFLAGMCQAYR(S)	10.90	4.39	1.00	1096.0395	5.83		
1	1.2	8	6	101.02	9.0	3.21E+05	70876.8	5.45	*Macrovipera lebetina*	Q4VM08	Zinc metalloproteinase-disintegrin-like VLAIP-A	3	14.79	14.79	78.2	2.92E+05	(R)KIPCAPQDVK(C)	4.62	5.40	0.99	385.8792	8.59	1.19%	0.40%
												2	18.45	18.45	93.4	1.56E+05	(R)KIPCAPQDVK(C)	4.62	5.40	1.00	578.3145	8.59		
												2	19.25	19.25	94.9	1.43E+06	(K)IPCAPQDVK(C)	4.73	0.00	0.99	514.2683	5.84		
												2	16.08	16.08	76.9	2.16E+05	(K)NPCQIYYTPSDENK(G)	5.35	0.00	0.98	864.8837	4.37		
												2	18.59	18.59	82.7	3.18E+05	(R)LYCFDNLPEHK(N)	6.17	0.00	1.00	718.3401	5.32		
												2	12.88	5.61	77.8	1.02E+05	(K)VTLDLFGK(W)	7.75	5.40	1.00	446.7604	5.81		
												2	15.58	8.39	77.0	4.34E+04	(R)IYEIVNILNVIYR(V)	10.72	0.00	0.90	811.4697	6.00		
												3	15.77	15.77	86.9	9.36E+03	(R)IYEIVNILNVIYR(V)	10.72	0.00	0.81	541.3154	6.00		
1	1.3	6	4	63.68	9.4	4.85E+05	55887.4	5.44	*Daboia russelii*	CL3662.contig2_DrSL	Zinc metalloproteinase-disintegrin VLAIP-A	3	16.70	16.70	84.2	1.59E+06	(K)LTQGSQCADEECCDQCK(F)	3.92	3.20	0.95	696.9297	3.91	1.79%	0.60%
												2	23.21	23.21	91.7	9.89E+05	(K)LTQGSQCADEECCDQCK(F)	3.92	4.28	0.98	1044.8892	3.91		
												2	11.82	4.48	83.5	1.77E+05	(K)INVLPEAK(V)	5.63	0.00	1.00	442.2667	6.00		
												2	12.88	5.61	77.8	1.02E+05	(K)VTLDLFGK(W)	7.75	5.40	1.00	446.7604	5.81		
												2	15.58	8.39	77.0	4.34E+04	(R)IYEIVNILNVIYR(V)	10.72	0.00	0.90	811.4697	6.00		
												3	15.77	15.77	86.9	9.36E+03	(R)IYEIVNILNVIYR(V)	10.72	0.00	0.81	541.3154	6.00		
2	2.1	13	11	194.73	19.5	9.40E+04	98156.9	7.36	*Daboia russelii*	CL3655.contig2_DrSL	phosphodiesterase 1	2	12.06	2.77	83.9	1.01E+05	(K)SIPFEAR(V)	5.47	5.41	0.92	410.2211	5.72	0.35%	0.12%
												2	12.92	12.92	75.6	2.51E+05	(K)QPLPETLQLK(T)	6.55	5.40	1.00	583.8446	6.00		
												2	18.94	18.94	88.0	2.09E+05	(K)AATYFWPGSEVK(I)	7.33	5.40	1.00	678.3371	6.04		
												2	17.52	17.52	84.1	1.19E+05	(K)DFYTFDSEGIVR(N)	7.73	5.40	0.99	724.8400	4.03		
												3	18.49	18.49	82.6	1.49E+05	(R)NPAWWGGQPIWHTVTYQGLK(A)	8.70	0.00	1.00	780.3968	8.60		
												2	26.33	26.33	97.6	4.68E+04	(R)NPAWWGGQPIWHTVTYQGLK(A)	8.70	0.00	0.99	1170.0880	8.60		
												3	10.57	4.46	77.4	5.79E+04	(R)NGLNVISGPIFDYNYDGHFDSYDTIK(E)	8.90	5.40	0.98	988.7943	4.13		
												2	12.52	6.93	85.8	1.00E+05	(K)TFLPIFVNPVN(-)	9.68	0.00	1.00	630.8534	5.18		
												2	22.27	22.27	98.0	1.09E+04	(K)FGPVSGEIIMALQMADR(T)	9.85	0.00	0.75	917.9640	4.37		
												3	21.14	21.14	90.1	7.93E+04	(K)DQCASSSAAQCPAGFEQSPLILFSMDGFR(A)	10.35	4.64	0.99	1059.8070	4.03		
												3	20.22	20.22	94.9	4.98E+04	(K)GKNEVTSFENIEVYNLMCDLLK(L)	10.52	5.40	0.98	872.7617	4.41		
												3	20.24	15.48	96.9	3.41E+04	(K)NEVTSFENIEVYNLMCDLLK(L)	11.08	5.40	0.97	811.0564	4.00		
												2	19.99	12.52	93.0	1.44E+04	(K)NEVTSFENIEVYNLMCDLLK(L)	11.08	5.40	0.96	1216.0776	4.00		
3	3.1	8	6	104.83	32.3	2.48E+06	29507.5	9.78	*Daboia siamensis*	P18965	Factor V activator RVV-V gamma	2	16.32	11.34	89.4	2.49E+06	(R)TLCAGILK(G)	5.75	5.40	0.98	438.2566	8.41	9.16%	3.05%
												2	12.78	12.78	76.5	1.13E+06	(R)EWVLTAAHCDR(R)	5.78	5.40	0.99	679.3220	5.32		
												3	17.66	17.66	95.2	2.65E+06	(R)RPVTYSTHIAPVSLPSR(S)	5.90	0.00	0.99	627.6870	10.84		
												2	12.10	12.10	80.0	3.76E+05	(R)RPVTYSTHIAPVSLPSR(S)	5.90	0.00	0.98	941.0244	10.84		
												3	20.50	8.70	94.4	6.06E+06	(K)ISTTEDTYPDVPHCTNIFIVK(H)	7.57	5.40	0.95	817.4066	4.54		
												2	17.82	12.70	86.7	6.42E+05	(K)ISTTEDTYPDVPHCTNIFIVK(H)	7.57	5.40	1.00	1225.6026	4.54		
												3	19.53	19.53	91.8	3.48E+05	(K)FPNGLDKDIMLIR(L)	7.97	5.40	1.00	511.2827	5.96		
												2	18.04	18.04	83.3	6.12E+06	(K)WCEPLYPWVPADSR(T)	8.45	16.21	0.99	888.4005	4.37		
3	3.2	4	3	53.31	20.0	5.82E+05	28908.7	9.10	*Daboia russelii*	CL2958.contig11_DrSL	serine beta-fibrinogenase-like protein	2	12.78	12.78	76.5	1.13E+06	(R)EWVLTAAHCDR(R)	5.78	5.40	0.99	679.3220	5.32	2.15%	0.72%
												3	19.53	19.53	91.8	3.48E+05	(K)FPNGLDKDIMLIR(L)	7.97	5.40	1.00	511.2827	5.96		
												3	21.00	11.30	95.7	7.34E+05	(R)LNSPVTYNTHIAPFSLPSSPPTVGSVCR(I)	8.03	10.81	0.96	1000.1757	8.75		

												4	13.17	4.01	74.2	1.15E+05	(R)LNSPVTYNTHIAPFSLPSSPPTVGSVCR(I)	8.03	10.81	0.98	750.3821	8.75		
4	4.1	24	5	104.76	67.3	2.58E+06	16276.8	4.51	*Daboia siamensis*	P31100	Acidic phospholipase A2 RV-7	2	21.60	21.60	90.3	4.08E+06	(R)CCFVHDCCYGTVNDCNPK(T)	5.22	21.61	0.88	1153.4361	5.21	9.53%	3.18%
												2	12.69	12.69	86.1	3.62E+06	(K)NYEYYSISHCTEESEQC(-)	5.85	27.02	0.99	1099.9214	4.09		
												3	17.62	17.62	84.5	2.12E+06	(R)AAAICLGQNVNTYDK(N)	6.12	27.15	1.00	546.6057	5.88		
												2	21.75	21.75	100.0	2.34E+06	(K)EVVHSYAIYGCYCGWGGQGR(A)	6.77	32.42	1.00	1160.0110	6.85		
												2	20.92	20.92	85.3	1.58E+06	(R)AAAICLGQNVNTYDK(N)	8.03	113.48	0.98	819.3999	5.88		
												3	20.69	20.69	92.2	1.88E+06	(K)EVVHSYAIYGCYCGWGGQGR(A)	9.00	211.07	1.00	773.6750	6.85		
												2	15.84	15.84	82.4	3.20E+05	(K)EVVHSYAIYGCYCGWGGQGR(A)	8.27	91.86	0.97	1160.0072	6.85		
												2	20.55	20.55	87.0	4.35E+06	(R)AAAICLGQNVNTYDK(N)	11.80	228.70	0.99	819.4028	5.88		
												2	24.87	24.87	94.8	7.29E+05	(K)TATYSYSFENGDIVCGDNDLCLR(T)	8.97	0.00	0.37	1335.5737	3.84		
												2	12.96	12.96	79.2	1.74E+04	(K)NYEYYSISHCTEESEQC(-)	10.93	4.39	0.98	1099.9176	4.09		
												3	15.34	15.34	79.4	6.75E+03	(R)AAAICLGQNVNTYDK(N)	11.63	0.00	0.80	546.6044	5.88		
												3	23.12	23.12	94.9	7.41E+06	(K)EVVHSYAIYGCYCGWGGQGR(A)	12.25	210.19	1.00	773.6750	6.85		
												2	19.15	19.15	80.9	7.56E+06	(R)AAAICLGQNVNTYDK(N)	12.77	159.31	0.93	819.4032	5.88		
												3	12.83	12.83	71.5	1.02E+04	(R)AAAICLGQNVNTYDK(N)	12.32	0.13	0.80	546.6044	5.88		
												3	23.30	23.30	92.5	1.16E+05	(K)TATYSYSFENGDIVCGDNDLCLR(T)	15.73	0.13	0.83	890.7197	3.84		
												2	24.99	24.99	96.7	3.59E+04	(K)TATYSYSFENGDIVCGDNDLCLR(T)	14.32	0.13	0.88	1335.5750	3.84		
												2	20.91	20.91	90.8	4.64E+04	(R)AAAICLGQNVNTYDK(N)	13.33	0.25	1.00	819.4032	5.88		
												3	24.29	24.29	97.3	1.65E+07	(K)EVVHSYAIYGCYCGWGGQGR(A)	14.92	217.88	1.00	773.6750	6.85		
												3	23.94	23.94	95.0	1.16E+05	(K)TATYSYSFENGDIVCGDNDLCLR(T)	15.73	0.13	0.83	890.7197	3.84		
												2	23.16	23.16	91.0	1.04E+04	(K)TATYSYSFENGDIVCGDNDLCLR(T)	15.28	0.00	0.85	1335.5750	3.84		
												2	20.21	20.21	84.5	7.63E+03	(R)AAAICLGQNVNTYDK(N)	15.23	0.00	0.80	819.4032	5.88		
												3	22.08	22.08	94.5	8.96E+06	(K)EVVHSYAIYGCYCGWGGQGR(A)	16.38	156.44	0.99	773.6750	6.85		
												3	23.33	23.33	93.4	3.82E+04	(K)TATYSYSFENGDIVCGDNDLCLR(T)	17.03	0.00	0.86	890.7209	3.84		
												3	20.17	20.17	84.4	1.80E+04	(K)TATYSYSFENGDIVCGDNDLCLR(T)	21.35	0.00	0.87	890.7209	3.84		
5	5.1	10	5	79.60	44.9	2.95E+06	16410.4	9.99	*Daboia siamensis*	Q02471	Basic phospholipase A2 RV-4	2	11.69	11.69	71.2	7.69E+03	(R)TICECDR(V)	2.27	0.58	1.00	477.1943	4.37	10.91%	3.64%
												2	11.25	11.25	71.3	1.78E+05	(R)GNIVCGR(N)	3.65	8.68	0.96	388.1978	9.75		
												2	20.47	20.47	77.8	2.28E+05	(R)CCFVHDCCYGGVK(G)	6.25	21.75	1.00	831.3237	6.74		
												3	11.77	11.77	71.9	1.18E+05	(K)LAIYSYSFQR(G)	6.88	16.21	1.00	416.5532	8.59		
												3	20.98	20.98	76.3	5.09E+06	(K)LGAFSVWNYISYGCYCGWGGQGTPK(D)	10.05	82.40	0.98	943.4277	8.43		
												2	24.17	24.17	83.9	2.16E+06	(K)LGAFSVWNYISYGCYCGWGGQGTPK(D)	9.88	50.99	0.98	1414.6357	8.43		
												3	24.14	24.14	90.2	8.74E+06	(K)LGAFSVWNYISYGCYCGWGGQGTPK(D)	12.02	120.76	0.98	943.4274	8.43		
												2	16.69	16.69	70.1	1.17E+05	(K)LGAFSVWNYISYGCYCGWGGQGTPK(D)	10.85	20.60	0.99	1414.6325	8.43		
												3	24.42	24.42	90.5	5.74E+06	(K)LGAFSVWNYISYGCYCGWGGQGTPK(D)	13.23	62.96	0.99	943.4254	8.43		
												3	22.01	22.01	89.3	7.13E+06	(K)LGAFSVWNYISYGCYCGWGGQGTPK(D)	15.55	126.37	0.95	943.4254	8.43		
6	6.1	5	4	54.61	32.2	5.32E+05	18793.1	5.84	*Daboia siamensis*	Q4PRD2	Snaclec coagulation factor X-activating enzyme light chain 2	3	20.90	20.90	83.6	3.57E+05	(K)DKEQECSSEWSDGSSVSYDK(L)	5.27	27.02	0.97	774.9853	4.02	1.97%	0.66%
												2	24.45	24.45	100.0	1.43E+05	(K)DKEQECSSEWSDGSSVSYDK(L)	5.27	27.02	0.97	1161.9713	4.02		
												2	11.43	0.99	76.4	9.76E+05	(K)CFVLEK(E)	5.38	5.40	1.00	398.2083	6.00		
												2	19.41	15.32	94.1	1.08E+06	(-)LDCPPDSSLYR(Y)	5.55	5.40	1.00	661.8088	4.21		
												3	18.73	18.73	92.7	1.06E+05	(R)FCMEHPNNGHLVSIESMEEAEFVAK(L)	7.62	0.00	0.95	969.1070	4.62		
7	7.1	5	4	68.59	19.6	8.51E+05	29126.9	7.94	*Daboia russelii*	CL2958.contig6_DrSL	Serine protease VLSP-1	2	18.14	18.14	94.2	6.57E+05	(K)IYDYSVCR(A)	5.00	0.00	1.00	538.2505	5.83	3.15%	1.05%
												2	16.32	11.34	89.4	2.49E+06	(R)TLCAGILK(G)	5.75	5.40	0.98	438.2566	8.41		
												2	13.13	4.06	74.8	2.58E+05	(K)SFTPWDK(D)	6.17	0.00	0.99	440.7148	5.55		
												3	21.00	11.30	95.7	7.34E+05	(R)LNSPVTYNTHIAPFSLPSSPPTVGSVCR(I)	8.03	10.81	0.96	1000.1757	8.75		
												4	13.17	4.01	74.2	1.15E+05	(R)LNSPVTYNTHIAPFSLPSSPPTVGSVCR(I)	8.03	10.81	0.98	750.3821	8.75		
8	8.1	6	3	67.44	36.5	1.52E+06	14867.6	5.36	*Daboia siamensis*	Q4PRD1	Snaclec coagulation factor X-activating enzyme light chain 1	2	18.91	18.91	84.0	5.30E+05	(K)SMTCNFIAPVVCK(F)	6.93	16.21	1.00	763.8646	8.47	5.63%	1.88%
												3	21.82	21.82	86.8	3.29E+06	(-)VLDCPSGWLSYEQHCYK(G)	7.12	5.40	0.98	714.6562	5.32		
												2	22.31	22.31	89.6	7.78E+05	(-)VLDCPSGWLSYEQHCYK(G)	7.12	5.40	1.00	1071.4788	5.32		
												3	22.46	12.47	93.2	3.65E+06	(K)ALAEESYCLIMITHEK(E)	7.70	5.40	0.90	636.6505	4.75		
												2	26.22	21.64	100.0	8.85E+05	(K)ALAEESYCLIMITHEK(E)	7.70	0.00	1.00	954.4689	4.75		
												3	18.45	11.66	81.4	4.77E+03	(K)ALAEESYCLIMITHEK(E)	10.35	0.00	0.57	636.6487	4.75		
8	8.2	5	3	66.98	28.7	1.16E+06	17267.5	5.63	*Daboia siamensis*	Q4PRC9	Snaclec 4	2	18.91	18.91	84.0	5.30E+05	(K)SMTCNFIAPVVCK(F)	6.93	16.21	1.00	763.8646	8.47	4.28%	1.43%
												2	21.85	21.85	87.8	7.17E+05	(K)VFTEEMNWADAEK(F)	6.97	16.21	0.99	785.3516	4.00		
												3	22.46	12.47	93.2	3.65E+06	(K)ALAEESYCLIMITHEK(V)	7.70	5.40	0.90	636.6505	4.75		
												2	26.22	21.64	100.0	8.85E+05	(K)ALAEESYCLIMITHEK(V)	7.70	0.00	1.00	954.4689	4.75		
												3	18.45	11.66	81.4	4.77E+03	(K)ALAEESYCLIMITHEK(V)	10.35	0.00	0.57	636.6487	4.75		
9	9.1	5	3	62.75	46.6	1.00E+06	10503.8	9.75	*Daboia siamensis*	A8Y7N7	Kunitz-type serine protease inhibitor C4	2	13.68	5.91	79.2	5.01E+05	(R)SFYYDSESK(K)	4.90	0.00	1.00	563.2414	4.37	3.72%	1.24%
												3	22.76	22.76	89.9	1.84E+06	(K)EFIYGGCHGNANNFPTR(D)	5.78	10.81	0.95	651.9624	6.85		
												2	29.22	29.22	100.0	4.15E+05	(K)EFIYGGCHGNANNFPTR(D)	5.80	10.81	0.94	977.4399	6.85		
												2	19.85	19.85	85.7	1.85E+06	(K)FCYLPADPGECMAYIR(S)	7.97	0.00	0.94	981.9344	4.37		
												3	18.90	18.90	74.1	4.17E+05	(K)FCYLPADPGECMAYIR(S)	7.97	0.00	0.98	654.9581	4.37		
10	10.1	5	3	61.86	39.2	7.88E+05	9712.8	6.82	*Daboia siamensis*	A8Y7P4	Kunitz-type serine protease inhibitor B4	2	13.46	4.18	87.7	2.64E+05	(R)RIYYNLESNK(C)	4.82	0.00	1.00	650.3402	8.50	2.91%	0.97%
												2	19.54	19.54	93.8	1.18E+06	(R)IYYNLESNK(C)	5.43	5.40	1.00	572.2899	6.00		
												3	24.64	24.64	95.0	1.25E+06	(K)CEVFFYGGCGGNDNNFSTWDECR(H)	8.02	10.81	0.89	931.0264	3.91		
												2	28.86	28.86	97.8	1.24E+06	(K)CEVFFYGGCGGNDNNFSTWDECR(H)	8.02	10.81	0.88	1396.0333	3.91		
												3	18.52	18.52	72.6	5.93E+03	(K)CEVFFYGGCGGNDNNFSTWDECR(H)	10.35	0.00	0.65	931.0257	3.91		
10	10.2	5	3	52.06	40.0	1.97E+06	7191.8	9.69	*Daboia siamensis*	P00990	Kunitz-type serine protease inhibitor 2	2	13.46	4.18	87.7	2.64E+05	(R)RIYYNLESNK(C)	4.82	0.00	1.00	650.3402	8.50	7.28%	2.43%
												3	19.06	6.98	94.5	4.41E+06	(-)HDRPTFCNLAPESGR(C)	5.10	5.40	0.94	586.2826	6.75		
												4	15.29	15.29	85.9	2.31E+06	(-)HDRPTFCNLAPESGR(C)	5.10	5.40	1.00	439.9631	6.75		
												2	16.81	12.03	94.9	1.68E+06	(-)HDRPTFCNLAPESGR(C)	5.08	0.00	1.00	878.9182	6.75		
												2	19.54	19.54	93.8	1.18E+06	(R)IYYNLESNK(C)	5.43	5.40	1.00	572.2899	6.00		
10	10.3	3	3	51.27	23.8	1.44E+06	9685.9	9.86	*Daboia siamensis*	A8Y7P6	Kunitz-type serine protease inhibitor B6	2	18.27	18.27	81.7	2.87E+06	(K)FCNLAPESGR(C)	4.90	5.40	0.94	575.7701	6.00	5.32%	1.77%
												2	13.46	4.18	87.7	2.64E+05	(R)RIYYNLESNK(C)	4.82	0.00	1.00	650.3402	8.50		
												2	19.54	19.54	93.8	1.18E+06	(R)IYYNLESNK(C)	5.43	5.40	1.00	572.2899	6.00		
11	11.1	3	3	59.33	42.2	3.18E+06	10352.6	10.41	*Daboia siamensis*	A8Y7N4	Kunitz-type serine protease inhibitor C1	2	17.65	12.79	91.3	1.53E+06	(R)IYYNPDSNK(C)	4.63	0.00	1.00	557.2670	5.83	11.75%	3.92%
												2	18.27	18.27	81.7	2.87E+06	(K)FCNLAPESGR(C)	4.90	5.40	0.94	575.7701	6.00		
												2	23.41	23.41	94.0	5.13E+06	(K)CEVFFYGGCGGNDNNFETR(K)	7.27	16.21	0.93	1121.9540	4.14		
12	12.1	4	3	43.67	20.5	5.40E+05	29180.4	9.89	*Daboia siamensis*	E5L0E3	Alpha-fibrinogenase-like	3	12.03	12.03	75.8	1.81E+06	(R)VIYRPLPEQSR(T)	5.78	5.40	1.00	453.2585	8.72	2.00%	0.67%
												3	16.72	8.94	84.7	4.65E+04	(K)ILPFVPHCANINIVPYTVCR(V)	8.70	0.00	0.98	795.0860	8.75		
												3	14.92	4.28	85.6	1.61E+05	(K)VFDYTDWIHSIIAGNTAATCPS(-)	10.08	5.40	1.00	813.7154	4.20		
												2	14.55	9.81	87.4	1.43E+05	(K)VFDYTDWIHSIIAGNTAATCPS(-)	10.08	5.40	1.00	1220.0687	4.20		
13	13.1	3	2	42.08	21.3	9.29E+05	13625.1	9.69	*Daboia russelii*	P30894	Venom nerve growth factor	2	23.10	23.10	96.5	1.17E+06	(R)INTACVCVISR(K)	5.63	0.00	0.98	646.8281	9.75	3.43%	1.14%
												2	18.98	12.42	85.4	8.99E+05	(K)HWNSYCTTTDTFVR(A)	5.90	0.00	0.99	894.3978	6.74		
												3	17.33	17.33	73.8	7.17E+05	(K)HWNSYCTTTDTFVR(A)	5.90	0.00	0.99	596.6008	6.74		
14	14.1	3	2	36.50	33.0	1.59E+06	13010.7	7.20	*Daboia siamensis*	P0DL42	Snake venom vascular endothelial growth factor toxin VR-1	2	12.74	12.74	71.8	9.37E+05	(K)HTADIQIMR(M)	4.90	0.00	1.00	542.7838	6.74	5.88%	1.96%
												3	23.76	18.79	97.0	3.46E+06	(R)ETLVSILQEHPDEISDIFRPSCVAVLR(C)	9.38	10.81	1.00	1041.8799	4.50		
												5	14.29	4.74	82.4	3.74E+05	(R)ETLVSILQEHPDEISDIFRPSCVAVLR(C)	9.38	10.81	0.99	625.5314	4.50		
15	15.1	3	2	32.88	14.5	1.13E+06	18452.4	5.38	*Daboia siamensis*	K9JBV0	P68 alpha subunit	2	12.70	12.70	73.3	1.83E+05	(K)NCFGLEK(Q)	4.92	10.81	0.86	434.2058	6.00	4.18%	1.39%
												2	20.12	20.12	90.8	2.55E+06	(R)TWFNLSCGDDYPFVCK(F)	8.87	0.00	0.93	1004.9350	4.21		
												3	20.18	20.18	92.2	6.61E+05	(R)TWFNLSCGDDYPFVCK(F)	8.87	0.00	1.00	670.2918	4.21		
16	16.1	1	1	23.09	5.8	2.88E+05	28981.6	7.11	*Macrovipera lebetina*	E0Y419	Beta-fibrinogenase	2	23.09	23.09	94.3	2.88E+05	(R)TLCAGILQGGIDTCK(G)	6.80	21.62	0.98	803.9028	5.50	1.07%	0.36%
17	17.1	1	1	21.27	2.9	3.83E+04	77077.2	5.92	*Boiga irregularis*	A0A0B8RNS9	Xaa-Pro aminopeptidase 2	3	21.27	17.38	93.6	3.83E+04	(K)KPTAILLSGLEETAWLFNLR(G)	11.07	5.40	0.95	758.0952	6.14	0.14%	0.05%
						2.70E+07																	100.00%	33.33%

Daboia siamensis Taiwan venom protein (n=3) sorted according to protein families

ample	Group	Subgroup	Spectra	Distinct Peptides	Distinct Summed MS/MS Search Score	% AA Coverage	Mean Protein Spectral Intensity	Protein MW (Da)	Protein pI	Species	Database Accession	Protein Name	Relative abundance (% overall)	Relative abundance of subtype (% overall)	Protein subtype
Kunitz-type serine protease inhibitor (KSPI)													28.21%	28.21%	5
T1	12	12.1	2	2	42.36	31.1	3.51E+06	10352.6	10.41	*Daboia siamensis*	A8Y7N4	Kunitz-type serine protease inhibitor C1	4.22%	12.18%	1
T2	13	13.1	3	2	41.06	31.1	3.01E+06	10352.6	10.41	*Daboia siamensis*	A8Y7N4	Kunitz-type serine protease inhibitor C1	4.05%		1
T3	11	11.1	3	3	59.33	42.2	3.18E+06	10352.6	10.41	*Daboia siamensis*	A8Y7N4	Kunitz-type serine protease inhibitor C1	3.92%		1
T1	7	7.1	4	3	64.03	46.6	8.26E+05	10503.8	9.75	*Daboia siamensis*	A8Y7N7	Kunitz-type serine protease inhibitor C4	0.99%	3.51%	2
T2	8	8.1	4	3	65.58	46.6	9.50E+05	10503.8	9.75	*Daboia siamensis*	A8Y7N7	Kunitz-type serine protease inhibitor C4	1.28%		2
T3	9	9.1	5	3	62.75	46.6	1.00E+06	10503.8	9.75	*Daboia siamensis*	A8Y7N7	Kunitz-type serine protease inhibitor C4	1.24%		2
T1	11	11.1	3	2	47.10	38.0	1.01E+06	9712.8	6.82	*Daboia siamensis*	A8Y7P4	Kunitz-type serine protease inhibitor B4	1.21%	3.43%	3
T2	11	11.1	3	2	49.01	38.0	9.30E+05	9712.8	6.82	*Daboia siamensis*	A8Y7P4	Kunitz-type serine protease inhibitor B4	1.25%		3
T3	10	10.1	5	3	61.86	39.2	7.88E+05	9712.8	6.82	*Daboia siamensis*	A8Y7P4	Kunitz-type serine protease inhibitor B4	0.97%		3
T3	10	10.3	3	3	51.27	23.8	1.44E+06	9685.9	9.86	*Daboia siamensis*	A8Y7P6	Kunitz-type serine protease inhibitor B6	1.77%	1.77%	4
T1	11	11.2	3	2	36.60	38.3	1.25E+06	7191.8	9.69	*Daboia siamensis*	P00990	Kunitz-type serine protease inhibitor 2	1.50%	7.31%	5
T2	11	11.2	4	2	38.56	38.3	2.52E+06	7191.8	9.69	*Daboia siamensis*	P00990	Kunitz-type serine protease inhibitor 2	3.38%		5
T3	10	10.2	5	3	52.06	40.0	1.97E+06	7191.8	9.69	*Daboia siamensis*	P00990	Kunitz-type serine protease inhibitor 2	2.43%		5
Phospholipase A2 (PLA2)													24.47%	24.47%	2
T1	5	5.1	22	5	105.42	67.3	3.50E+06	16276.8	4.51	*Daboia siamensis*	P31100	Acidic phospholipase A2 RV-7	4.21%	10.87%	1
T2	4	4.1	22	5	103.80	67.3	2.58E+06	16276.8	4.51	*Daboia siamensis*	P31100	Acidic phospholipase A2 RV-7	3.48%		1
T3	4	4.1	24	5	104.76	67.3	2.58E+06	16276.8	4.51	*Daboia siamensis*	P31100	Acidic phospholipase A2 RV-7	3.18%		1
T1	8	8.1	8	3	57.91	32.6	5.22E+06	16410.4	9.99	*Daboia siamensis*	Q02471	Basic phospholipase A2 RV-4	6.27%	13.61%	2
T2	6	6.1	9	4	69.34	39.8	2.75E+06	16410.4	9.99	*Daboia siamensis*	Q02471	Basic phospholipase A2 RV-4	3.70%		2
T3	5	5.1	10	5	79.60	44.9	2.95E+06	16410.4	9.99	*Daboia siamensis*	Q02471	Basic phospholipase A2 RV-4	3.64%		2
Snake venom C-type lectin (Snaclec)													16.52%	16.52%	6
T1	9	9.1	4	3	55.76	21.5	1.49E+06	18452.4	5.38	*Daboia siamensis*	K9JBV0	P68 alpha subunit	1.79%	5.95%	1
T2	12	12.1	3	2	43.22	17.0	2.06E+06	18452.4	5.38	*Daboia siamensis*	K9JBV0	P68 alpha subunit	2.77%		1
T3	15	15.1	3	2	32.88	14.5	1.13E+06	18452.4	5.38	*Daboia siamensis*	K9JBV0	P68 alpha subunit	1.39%		1
T1	15	15.1	2	2	27.91	8.8	5.25E+05	18935.2	5.44	*Daboia siamensis*	K9JDJ1	factor X activator light chain 2	0.63%	1.28%	2
T2	15	15.1	2	2	25.71	8.8	4.81E+05	18935.2	5.44	*Daboia siamensis*	K9JDJ1	factor X activator light chain 2	0.65%		2
T3	8	8.2	5	3	66.98	28.7	1.16E+06	17267.5	5.63	*Daboia siamensis*	Q4PRC9	Snaclec 4	1.43%	1.43%	3
T1	6	6.2	3	2	34.57	20.9	4.80E+05	17366.4	6.03	*Daboia siamensis*	Q4PRD0	Snaclec 3	0.58%	1.03%	4
T2	18	18.1	3	2	22.12	12.8	3.33E+05	17366.4	6.03	*Daboia siamensis*	Q4PRD0	Snaclec 3	0.45%		4
T1	6	6.1	6	3	67.55	36.5	1.07E+06	14867.6	5.36	*Daboia siamensis*	Q4PRD1	Snaclec coagulation factor X-activating enzyme light chain 1	1.28%	4.77%	5
T2	7	7.1	5	3	65.90	36.5	1.20E+06	14867.6	5.36	*Daboia siamensis*	Q4PRD1	Snaclec coagulation factor X-activating enzyme light chain 1	1.61%		5
T3	8	8.1	6	3	67.44	36.5	1.52E+06	14867.6	5.36	*Daboia siamensis*	Q4PRD1	Snaclec coagulation factor X-activating enzyme light chain 1	1.88%		5
T1	10	10.1	4	3	33.60	16.4	5.46E+05	18793.1	5.84	*Daboia siamensis*	Q4PRD2	Snaclec coagulation factor X-activating enzyme light chain 2	0.66%	2.07%	6
T2	10	10.1	4	3	33.59	16.4	5.66E+05	18793.1	5.84	*Daboia siamensis*	Q4PRD2	Snaclec coagulation factor X-activating enzyme light chain 2	0.76%		6
T3	6	6.1	5	4	54.61	32.2	5.32E+05	18793.1	5.84	*Daboia siamensis*	Q4PRD2	Snaclec coagulation factor X-activating enzyme light chain 2	0.66%		6
Snake venom serine protease (SVSP)													17.51%	17.51%	6
T2	17	17.1	1	1	22.12	5.8	2.86E+05	28981.6	7.11	*Macrovipera lebetina*	E0Y419	Beta-fibrinogenase	0.38%	0.74%	1
T3	16	16.1	1	1	23.09	5.8	2.88E+05	28981.6	7.11	*Macrovipera lebetina*	E0Y419	Beta-fibrinogenase	0.36%		1
T1	16	16.1	3	2	26.51	12.7	1.33E+05	29180.4	9.89	*Daboia siamensis*	E5L0E3	Alpha-fibrinogenase-like	0.16%	1.01%	2
T2	16	16.1	3	2	25.08	12.7	1.40E+05	29180.4	9.89	*Daboia siamensis*	E5L0E3	Alpha-fibrinogenase-like	0.19%		2
T3	12	12.1	4	3	43.67	20.5	5.40E+05	29180.4	9.89	*Daboia siamensis*	E5L0E3	Alpha-fibrinogenase-like	0.67%		2
T1	3	3.1	10	8	135.70	42.3	1.73E+06	29507.5	9.78	*Daboia siamensis*	P18965	Factor V activator RVV-V gamma	2.08%	8.11%	3
T2	3	3.1	8	7	124.24	38.4	2.21E+06	29507.5	9.78	*Daboia siamensis*	P18965	Factor V activator RVV-V gamma	2.98%		3
T3	3	3.1	8	6	104.83	32.3	2.48E+06	29507.5	9.78	*Daboia siamensis*	P18965	Factor V activator RVV-V gamma	3.05%		3
T1	3	3.3	5	4	66.46	23.8	6.68E+05	28908.7	9.10	*Daboia russelii*	CL2958.contig11_DrSL	serine beta-fibrinogenase-like protein	0.80%	2.38%	4
T2	3	3.2	4	3	50.61	20.0	6.41E+05	28908.7	9.10	*Daboia russelii*	CL2958.contig11_DrSL	serine beta-fibrinogenase-like protein	0.86%		4
T3	3	3.2	4	3	53.31	20.0	5.82E+05	28908.7	9.10	*Daboia russelii*	CL2958.contig11_DrSL	serine beta-fibrinogenase-like protein	0.72%		4
T1	4	4.1	9	7	120.42	30.8	8.66E+05	29126.9	7.94	*Daboia russelii*	CL2958.contig6_DrSL	Serine protease VLSP-1	1.04%	3.07%	5
T2	5	5.1	6	5	82.01	25.0	7.30E+05	29126.9	7.94	*Daboia russelii*	CL2958.contig6_DrSL	Serine protease VLSP-1	0.98%		5
T3	7	7.1	5	4	68.59	19.6	8.51E+05	29126.9	7.94	*Daboia russelii*	CL2958.contig6_DrSL	Serine protease VLSP-1	1.05%		5
T1	3	3.2	9	6	107.87	30.2	1.82E+06	27523.2	9.03	*Naja naja*	CL31.contig2_Nn	RVV-V gamma-like protein	2.19%	2.19%	6
Snake venom metalloproteinase (SVMP)													5.86%	5.86%	4
T1	1	1.2	6	5	77.37	9.0	3.02E+05	70876.8	5.45	*Macrovipera lebetina*	Q4VM08	Zinc metalloproteinase-disintegrin-like VLAIP-A	0.36%	1.34%	1
T2	1	1.2	8	6	102.45	9.0	4.35E+05	70876.8	5.45	*Macrovipera lebetina*	Q4VM08	Zinc metalloproteinase-disintegrin-like VLAIP-A	0.59%		1
T3	1	1.2	8	6	101.02	9.0	3.21E+05	70876.8	5.45	*Macrovipera lebetina*	Q4VM08	Zinc metalloproteinase-disintegrin-like VLAIP-A	0.40%		1
T1	1	1.1	22	15	266.26	25.6	6.84E+05	71928.8	5.98	*Daboia siamensis*	Q7LZ61	Coagulation factor X-activating enzyme heavy chain	0.82%	2.45%	2
T2	1	1.1	22	16	290.56	27.9	6.62E+05	71928.8	5.98	*Daboia siamensis*	Q7LZ61	Coagulation factor X-activating enzyme heavy chain	0.89%		2
T3	1	1.1	24	16	294.74	25.3	5.98E+05	71928.8	5.98	*Daboia siamensis*	Q7LZ61	Coagulation factor X-activating enzyme heavy chain	0.74%		2
T1	1	1.4	4	3	47.35	7.8	3.65E+05	55887.4	5.44	*Daboia russelii*	CL3662.contig2_DrSL	Zinc metalloproteinase-disintegrin VLAIP-A	0.44%	1.62%	3
T2	1	1.3	5	3	53.22	7.8	4.36E+05	55887.4	5.44	*Daboia russelii*	CL3662.contig2_DrSL	Zinc metalloproteinase-disintegrin VLAIP-A	0.59%		3
T3	1	1.3	6	4	63.68	9.4	4.85E+05	55887.4	5.44	*Daboia russelii*	CL3662.contig2_DrSL	Zinc metalloproteinase-disintegrin VLAIP-A	0.60%		3
T1	1	1.3	4	3	49.70	28.3	3.66E+05	12226.6	9.44	*Naja naja*	Unigene31385_Nn	Zinc metalloproteinase-disintegrin VLAIP-A	0.44%	0.44%	4
Snake venom vascular endothelial growth factor (svEGF)													4.84%	4.84%	1
T1	13	13.1	3	2	36.42	33.0	1.25E+06	13010.7	7.20	*Daboia siamensis*	P0DL42	Snake venom vascular endothelial growth factor toxin VR-1	1.50%	4.84%	1
T2	9	9.1	4	3	53.44	44.0	1.02E+06	13010.7	7.20	*Daboia siamensis*	P0DL42	Snake venom vascular endothelial growth factor toxin VR-1	1.38%		1
T3	14	14.1	3	2	36.50	33.0	1.59E+06	13010.7	7.20	*Daboia siamensis*	P0DL42	Snake venom vascular endothelial growth factor toxin VR-1	1.96%		1
Snake venom nerve growth factor (svNGF)													2.13%	2.13%	1
T2	19	19.1	2	1	21.02	11.9	7.32E+05	13625.1	9.69	*Daboia russelii*	P30894	Venom nerve growth factor	0.98%	2.13%	1
T3	13	13.1	3	2	42.08	21.3	9.29E+05	13625.1	9.69	*Daboia russelii*	P30894	Venom nerve growth factor	1.14%		1
Phosphodiesterase (PDE)													0.31%	0.31%	1
T1	2	2.1	13	12	209.80	22.0	7.28E+04	98156.9	7.36	*Daboia russelii*	CL3655.contig2_DrSL	phosphodiesterase 1	0.09%	0.31%	1
T2	2	2.1	14	14	243.81	24.4	8.16E+04	98156.9	7.36	*Daboia russelii*	CL3655.contig2_DrSL	phosphodiesterase 1	0.11%		1
T3	2	2.1	13	11	194.73	19.5	9.40E+04	98156.9	7.36	*Daboia russelii*	CL3655.contig2_DrSL	phosphodiesterase 1	0.12%		1
Aminopeptidase (non-toxin)													0.15%	0.15%	2
T1	14	14.1	3	2	31.84	5.5	2.62E+04	77077.2	5.92	*Boiga irregularis*	A0A0B8RNS9	Xaa-Pro aminopeptidase 2	0.03%	0.13%	1
T2	14	14.1	2	2	37.04	5.5	3.61E+04	77077.2	5.92	*Boiga irregularis*	A0A0B8RNS9	Xaa-Pro aminopeptidase 2	0.05%		1
T3	17	17.1	1	1	21.27	2.9	3.83E+04	77077.2	5.92	*Boiga irregularis*	A0A0B8RNS9	Xaa-Pro aminopeptidase 2	0.05%		1
T1	14	14.2	3	2	31.38	5.4	1.62E+04	74587.3	5.63	*Daboia russelii*	Unigene32033_DrSL	xaa-Pro aminopeptidase 2-like	0.02%	0.02%	2

Daboia siamensis Taiwan venom summary table

Protein Name	Database Accession	Species	Relative abundance of subtype (% overall)	Protein subtype
Kunitz-type serine protease inhibitor (KSPI)			28.21%	5
Kunitz-type serine protease inhibitor C1	A8Y7N4	*Daboia siamensis*	12.18%	1
Kunitz-type serine protease inhibitor C4	A8Y7N7	*Daboia siamensis*	3.51%	2
Kunitz-type serine protease inhibitor B4	A8Y7P4	*Daboia siamensis*	3.43%	3
Kunitz-type serine protease inhibitor B6	A8Y7P6	*Daboia siamensis*	1.77%	4
Kunitz-type serine protease inhibitor 2	P00990	*Daboia siamensis*	7.31%	5
Phospholipase A2 (PLA2)			24.47%	2
Acidic phospholipase A2 RV-7	P31100	*Daboia siamensis*	10.87%	1
Basic phospholipase A2 RV-4	Q02471	*Daboia siamensis*	13.61%	2
Snake venom C-type lectin (Snaclec)			16.52%	6
Snaclec 4	Q4PRC9	*Daboia siamensis*	1.43%	1
Snaclec 3	Q4PRD0	*Daboia siamensis*	1.03%	2
P68 alpha subunit	K9JBV0	*Daboia siamensis*	5.95%	3
Snaclec coagulation factor X-activating enzyme light chain 1	Q4PRD1	*Daboia siamensis*	4.77%	4
Snaclec coagulation factor X-activating enzyme light chain 2	Q4PRD2	*Daboia siamensis*	2.07%	5
factor X activator light chain 2	K9JDJ1	*Daboia siamensis*	1.28%	6
Snake venom serine protease (SVSP)			17.51%	6
Alpha-fibrinogenase-like	E5L0E3	*Daboia siamensis*	1.01%	1
Beta-fibrinogenase	E0Y419	*Macrovipera lebetina*	0.74%	2
serine beta-fibrinogenase-like protein	CL2958.contig11_DrSL	*Daboia russelii*	2.38%	3
Factor V activator RVV-V gamma	P18965	*Daboia siamensis*	8.11%	4
RVV-V gamma-like protein	CL31.contig2_Nn	*Naja naja*	2.19%	5
Serine protease VLSP-1	CL2958.contig6_DrSL	*Daboia russelii*	3.07%	6
Snake venom metalloproteinase (SVMP)			5.86%	4
Zinc metalloproteinase-disintegrin-like VLAIP-A	Q4VM08	*Macrovipera lebetina*	1.34%	1
Zinc metalloproteinase-disintegrin VLAIP-A	CL3662.contig2_DrSL	*Daboia russelii*	1.62%	2
Zinc metalloproteinase-disintegrin VLAIP-A	Unigene31385_Nn	*Naja naja*	0.44%	3
Coagulation factor X-activating enzyme heavy chain	Q7LZ61	*Daboia siamensis*	2.45%	4
Snake venom vascular endothelial growth factor (svEGF)			4.84%	1
Snake venom vascular endothelial growth factor toxin VR-1	P0DL42	*Daboia siamensis*	4.84%	1
Snake venom nerve growth factor (svNGF)			2.13%	1
Venom nerve growth factor	P30894	*Daboia russelii*	2.13%	1
Phosphodiesterase (PDE)			0.31%	1
phosphodiesterase 1	CL3655.contig2_DrSL	*Daboia russelii*	0.31%	1
Aminopeptidase (non-toxin)			0.15%	2
Xaa-Pro aminopeptidase 2	A0A0B8RNS9	*Boiga irregularis*	0.13%	1
xaa-Pro aminopeptidase 2-like	Unigene32033_DrSL	*Daboia russelii*	0.02%	2
